# Wild bonobos host geographically restricted malaria parasites including a putative new *Laverania* species

**DOI:** 10.1038/s41467-017-01798-5

**Published:** 2017-11-21

**Authors:** Weimin Liu, Scott Sherrill-Mix, Gerald H. Learn, Erik J. Scully, Yingying Li, Alexa N. Avitto, Dorothy E. Loy, Abigail P. Lauder, Sesh A. Sundararaman, Lindsey J. Plenderleith, Jean-Bosco N. Ndjango, Alexander V. Georgiev, Steve Ahuka-Mundeke, Martine Peeters, Paco Bertolani, Jef Dupain, Cintia Garai, John A. Hart, Terese B. Hart, George M. Shaw, Paul M. Sharp, Beatrice H. Hahn

**Affiliations:** 1 Department of Medicine, University of Pennsylvania, Philadelphia, PA 19104 USA; 2 Department of Microbiology, University of Pennsylvania, Philadelphia, PA 19104 USA; 3 Department of Human Evolutionary Biology, Harvard University, Cambridge, MA 02138 USA; 4 Department of Immunology and Infectious Diseases, Harvard T.H. Chan School of Public Health, Boston, MA 02115 USA; 5 Institute of Evolutionary Biology and Centre for Immunity, Infection and Evolution, University of Edinburgh, Edinburgh, EH9 3FL UK; 6 Department of Ecology and Management of Plant and Animal Resources, Faculty of Sciences, University of Kisangani, BP 2012 Kisangani, Democratic Republic of the Congo; 7 School of Biological Sciences, Bangor University, Bangor, LL57 2UW UK; 8 Institut National de Recherche Biomedicale, University of Kinshasa, BP 1197 Kinshasa, Democratic Republic of the Congo; 9 Unité Mixte Internationale 233, Institut de Recherche pour le Développement (IRD), INSERM U1175, University of Montpellier 1, BP 5045, Montpellier, 34394 France; 10 Leverhulme Centre for Human Evolutionary Studies, University of Cambridge, Cambridge, CB2 1QH UK; 11 African Wildlife Foundation Conservation Centre, P.O. Box 310, 00502 Nairobi, Kenya; 12 Lukuru Wildlife Research Foundation, Tshuapa-Lomami-Lualaba Project, BP 2012 Kinshasa, Democratic Republic of the Congo

## Abstract

Malaria parasites, though widespread among wild chimpanzees and gorillas, have not been detected in bonobos. Here, we show that wild-living bonobos are endemically *Plasmodium* infected in the eastern-most part of their range. Testing 1556 faecal samples from 11 field sites, we identify high prevalence *Laverania* infections in the Tshuapa-Lomami-Lualaba (TL2) area, but not at other locations across the Congo. TL2 bonobos harbour *P. gaboni*, formerly only found in chimpanzees, as well as a potential new species, *Plasmodium lomamiensis* sp. nov. Rare co-infections with non-*Laverania* parasites were also observed. Phylogenetic relationships among *Laverania* species are consistent with co-divergence with their gorilla, chimpanzee and bonobo hosts, suggesting a timescale for their evolution. The absence of *Plasmodium* from most field sites could not be explained by parasite seasonality, nor by bonobo population structure, diet or gut microbiota. Thus, the geographic restriction of bonobo *Plasmodium* reflects still unidentified factors that likely influence parasite transmission.

## Abstract (web summary)

Unlike chimpanzees and gorillas, bonobos have not been found infected by malaria parasites in the wild. Here, Liu et al. report more thorough survey and sequencing results showing that bonobos host malaria parasites, including a yet-unknown species, but only in the eastern-most part of their range.

## Introduction

African apes are highly endangered, requiring noninvasive approaches to study infectious agents in wild-living communities. To elucidate the origins and evolution of human malaria parasites, we^1–3^ and others^4–6^ have developed PCR-based methods that permit the faecal-based detection and molecular characterisation of related parasites in wild-living apes. Such studies have shown that chimpanzees (*Pan troglodytes*) and western gorillas (*Gorilla gorilla*) harbour a plethora of *Plasmodium* parasites, which fall into two major groups^7^. One group (subgenus *Plasmodium*) includes several *Plasmodium* species infecting monkeys as well as ape parasites that are closely related to human *P. malariae, P. ovale* and *P. vivax* ^7^. Of these, ape *P. vivax* is known to infect both chimpanzees and gorillas, while contemporary human *P. vivax* represents a lineage that emerged from these parasites as it spread out of Africa^2^. The other group (subgenus *Laverania*) includes ape parasites that are most closely related to human *P. falciparum* ^7^. There are currently six described ape *Laverania* species, which appear to exhibit strict host specificity in wild ape populations^1,3–5^. These include *P. reichenowi* (also termed C1), *P. gaboni* (C2), and *P. billcollinsi* (C3), which infect chimpanzees, as well as *P. praefalciparum* (G1), *P. adleri* (G2), and *P. blacklocki* (G3), which infect western gorillas. Of these, only the gorilla parasite *P. praefalciparum* has crossed the species barrier to humans, resulting in the emergence of *P. falciparum* ^1,7,8^. Although initially based primarily on mitochondrial sequences^1^, this taxonomy of *Laverania* species has subsequently been confirmed by analysis of multiple nuclear gene sequences^3,9^.

*Laverania* infections have been documented at multiple locations throughout the ranges of chimpanzees and western lowland gorillas (*G. g. gorilla*), with estimated prevalence rates in infected communities ranging between 22 and 40%^7^. Similarly, ape *P. vivax* is found in all chimpanzee subspecies as well as western and eastern gorillas (*G. beringei*), although estimated prevalence rates are lower, ranging between 4 and 8%^7^. Studies of Asian primates have shown that the distribution and prevalence of *Plasmodium* infections depends on a number of ecological variables, such as forest cover^10^, population density^11^, vector capacity^12^ and environmental conditions^13^, many of which are interrelated. Although the factors that promote and sustain malaria transmission in wild apes remain largely unknown, it is clear that *Plasmodium* species are not uniformly distributed among them. For example, eastern gorillas harbour ape *P. vivax*, but do not seem to carry *Laverania* parasites^1,2^. More strikingly, bonobos (*Pan paniscus*) appear to be free of all known ape *Plasmodium* species, despite the screening of multiple communities^1,2^.

The seeming absence of *Plasmodium* infections from wild bonobos has remained a mystery. *Anopheles* vectors, including forest species such as *A. moucheti*, *A. marshallii* and *A. vinckei*, which are known to carry ape *Plasmodium* parasites^14,15^ appear to be distributed throughout the bonobo range^16^. Bonobos are also very closely related to chimpanzees, suggesting a similar susceptibility to *Plasmodium* infection. Finally, there is no evidence that bonobos are inherently resistant to *Plasmodium* parasites, since human *P. falciparum* and *P. malariae* have been detected in the blood of several captive individuals^17^. Reasoning that previous studies may have missed infected communities, we conducted a more extensive survey, increasing both the number and geographic diversity of sampled bonobo populations. Here, we show that wild bonobos are, in fact, susceptible to a wide variety of *Plasmodium* parasites, including a previously unknown *Laverania* species that appears specific to bonobos. However, endemic infection was only detected in the eastern-most part of the bonobo range, indicating that most wild-living communities have lost these parasites.

## Results

### Bonobos are naturally *Laverania* infected

**Fig. 1:**
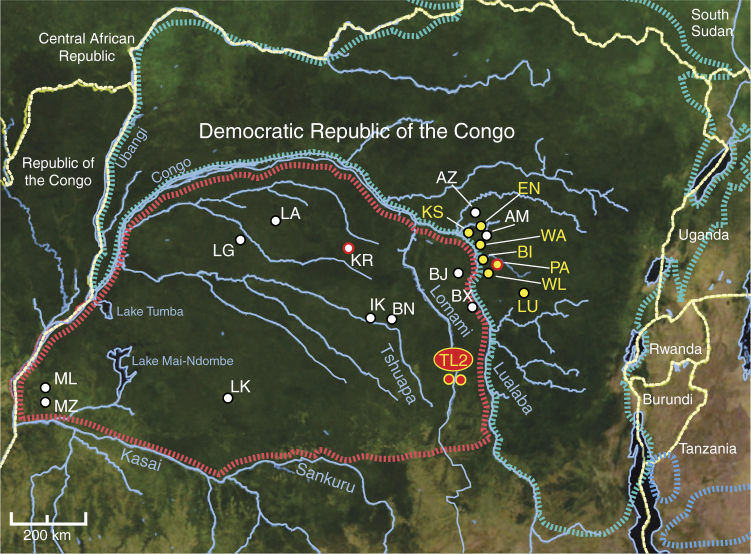
*Plasmodium* infections of wild-living bonobos. Ape study sites are shown in relation to the ranges of the bonobo (*P. paniscus*, dashed red) and the eastern chimpanzee (*P. t. schweinfurthii*, dashed blue), with white dots indicating sites where no *Plasmodium* infection was found (see Table 1 and Supplementary Table 3 for a list of all field sites and their code designation). The Tshuapa–Lomami–Lualaba (TL2) site where bonobos are endemically infected with multiple *Plasmodium* species, including a newly discovered *Laverania* species (B1), is shown in red with two dots indicating sampling on both sides of the Lomami River. Eastern chimpanzee field sites with endemic *P. reichenowi, P. gaboni* and/or *P. billcollinsi* infections are shown in yellow. A red circle highlights one bonobo (KR) and one chimpanzee (PA) field site where B1 parasite sequences were detected in a single faecal sample. Forested areas are shown in dark green, while arid or semiarid areas are depicted in brown. Major lakes and rivers are shown in blue. Dashed yellow lines indicate national boundaries. The scale bar indicates 200 km

Bonobos are found in the rain forests of the Congo Basin in the Democratic Republic of the Congo (DRC). Separated from eastern chimpanzees (*P. t. schweinfurthii*) and eastern lowland gorillas (*G. b. graueri*) by the Congo River, their range extends from the Lualaba River in the east, to the Kasai and Sankuru Rivers in the south, and the Lake Tumba and Lake Mai-Ndombe regions in the west (Fig. 1). Initial studies failed to identify *Plasmodium* infections in wild bonobos, but were conducted at only two locations (LK and KR)^1^. Although subsequent surveys included additional bonobo field sites (ML, LA, IK, BN, BJ, TL2), faecal samples were only tested for *P. vivax*-like parasites^2^. Here, we screened these (*n* = 646) as well as newly collected (*n* = 803) faecal samples from the same (LA, IK) and additional (LG, BX, MZ) study sites for *Laverania* infection (Fig. 1). Using conventional (diagnostic) PCR to amplify a 956 bp mitochondrial cytochrome B (*cytB*) fragment^1^, we failed to detect parasite sequences in 1418 samples from 10 of these 11 locations (Table 1). Surprisingly, however, 16 of 138 faecal specimens from the Tshuapa-Lomami-Lualaba (TL2) project site were *Laverania* positive as determined by direct amplicon sequencing (Table 1).

**Table 1:** Noninvasive screening of wild-living bonobo communities for *Laverania* infections

Field sites^a^	Conventional *cytB* screen			Intensified *cytB* screen^b^			
	Samples tested^c^	Samples positive^d^	Detection rate (%)	Samples tested^c^	Samples positive^d^	Detection rate (%)	Combined detection rate (%)
Balanga (BN)	84	0	0	84	0	0	0
Bananjale (BX)	1	0	0	1	0	0	0
Bayandjo (BJ)	2	0	0	2	0	0	0
Ikela (IK)	465	0	0	432^e^	0	0	0
Kokolopori (KR)	69	0	0	69	1	1.4	1.4^f^
Lingunda-Boyela (LG)	25	0	0	25	0	0	0
Lomako (LA)	307	0	0	289^e^	0	0	0
Lui-kotal (LK)	38	0	0	38	0	0	0
Malebo (ML)	262	0	0	n/a	n/a	n/a	n/a
Manzana (MZ)	165	0	0	165	0	0	0
Tshuapa–Lomami–Lualaba (TL2)	138	16	11.6	122	17	13.9	23.9^f^

*n/a* not available

^a^Field sites are designated by a two- or three-letter code (their location is shown in Fig. 1). Regular *cytB* screening data for KR and LK sites have previously been reported^2^

^b^Samples initially negative by conventional (diagnostic) *cytB* PCR were subsequently retested by intensified PCR, performing 8–10 additional amplification reactions per faecal DNA (see Supplementary Table 1 for details)

^c^The host species origin of all faecal samples was confirmed by mitochondrial DNA (D-loop) analysis. As previously reported, the number of individuals tested at the KR, LK and TL2 sites was 38, 17 and 63, respectively^3^

^d^All amplification products were sequence confirmed to represent *Laverania* parasites

^e^Only a subset of the originally screened IK and LA samples were available for intensified PCR

^f^Of a total of 38 and 63 bonobos at the KR and TL2 sites^1, 2^, 1 and 24 were *Laverania* infected, indicating a prevalence of 2.6% and 38%, respectively

Reasoning that conventional PCR screening may have missed low-level *Laverania* infection, we retested all available *cytB*-negative faecal specimens by subjecting them to an intensified PCR protocol. Since most ape faecal samples contain limited quantities of parasite DNA, we reasoned that testing multiple aliquots of the same DNA preparation would increase the likelihood of parasite detection. To avoid PCR contamination, only initially negative samples were re-tested using the intensified approach. Performing 8 to 10 independent PCR reactions for each DNA sample, we identified 17 additional faecal samples from TL2 to contain *cytB* sequences, resulting in a total of 33 positive specimens from 24 different apes (Table 1). Although in most cases only one or a few replicates yielded an amplification product (Supplementary Table 1), the intensified PCR approach more than doubled the number of positives at the TL2 site, revealing an overall *Laverania* prevalence of 38% (Table 1). However, this was not observed for other bonobo field sites. Intensified PCR of the remaining 1105 samples identified only a single additional positive specimen from the Kokolopori Reserve (KR). Thus, malaria parasites are either absent or below the limits of faecal detection at the vast majority of bonobo field sites.

### A new bonobo-specific *Laverania* species

**Fig. 2:**
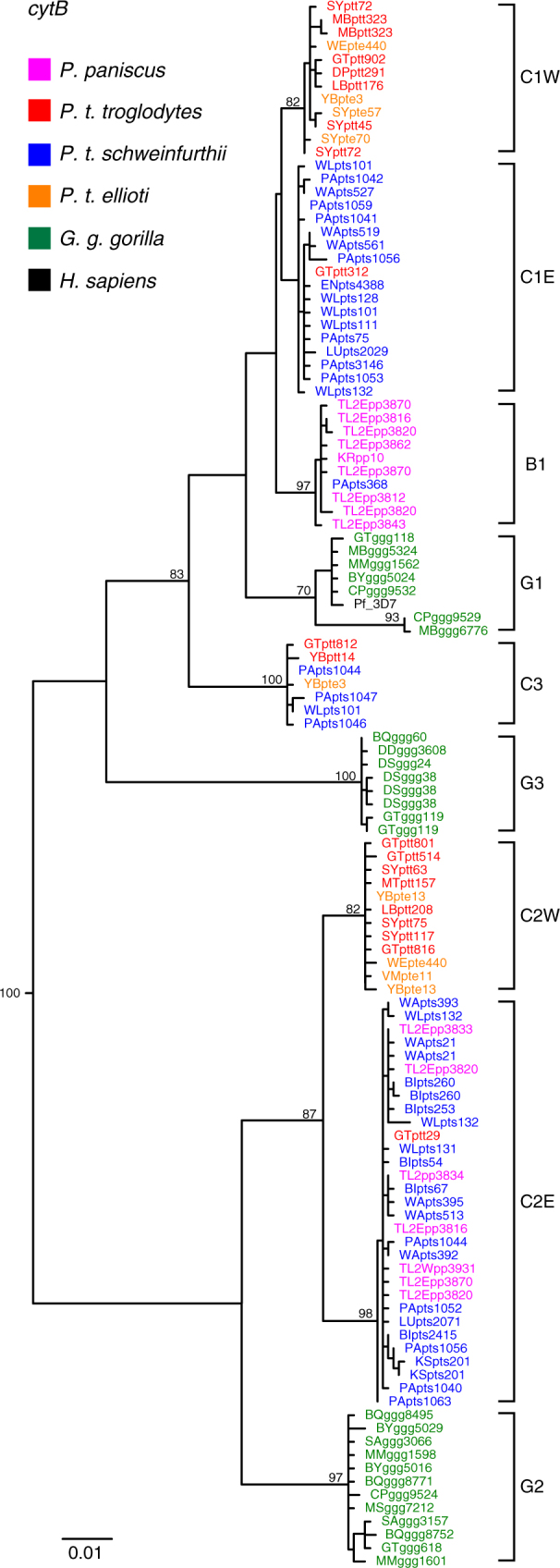
Relationship of bonobo parasites to ape *Laverania* species. A maximum likelihood tree of mitochondrial cytochrome B (*cytB*) sequences (956 bp) depicting the phylogenetic position of newly derived bonobo parasite sequences (magenta) is shown. Only distinct *cytB* haplotypes are depicted (the full set of SGA-derived bonobo parasite sequences is shown in Supplementary Fig. 1). Sequences are colour-coded, with capital letters indicating their field site of origin (see Fig. 1 for location of field sites) and lowercase letters denoting their host species and subspecies origin (ptt: *P. t. troglodytes*, red; pte: *P. t. ellioti*, orange; pts: *P. t. schweinfurthii*, blue; ggg: *G. g. gorilla*, green; pp: *Pan paniscus*, magenta). C1, C2 and C3 represent the chimpanzee parasites *P. reichenowi*, *P. gaboni* and *P. billcollinsi*; G1, G2 and G3 represent the gorilla parasites *P. praefalciparum, P. adleri* and *P. blacklocki* (the *P. falciparum* 3D7 reference sequence is shown in black). *P. reichenowi* (C1) and *P. gaboni* (C2) mitochondrial sequences are known to segregate into two geographically defined subclades according to their collection site in 'western' (W) or 'eastern' (E) Africa^3^. Bonobo parasite sequences (magenta) cluster with *P. gaboni* from eastern chimpanzees (C2E), but also form a new clade, termed B1. The tree was constructed using PhyML^58^ with TIM2+I+G as the evolutionary model. Bootstrap values are shown for major nodes only (the scale bar represents 0.01 substitutions per site)

Having identified *Laverania*-positive bonobo samples, we next sought to molecularly characterise the infecting parasites. Since apes are frequently co-infected with multiple *Plasmodium* species, we used limiting dilution PCR, also called single genome amplification (SGA), to generate mitochondrial *cytB* sequences (956 bp) devoid of *Taq* polymerase-induced artefacts such as *in vitro* recombination^18^. Using this approach, we generated 166 limiting dilution-derived *cytB* sequences from 34 *Laverania-*positive bonobo samples, including a unique haplotype from the single positive KR specimen (Supplementary Table 2). Phylogenetic analysis showed that these bonobo parasites fell into two well-supported clades within the *Laverania* subgenus (Fig. 2). One of these comprised a sublineage of *P. gaboni* (C2E) previously found in eastern chimpanzees (*P. t. schweinfurthii*) in the DRC^3^. Within this sublineage, bonobo and chimpanzee parasite sequences were completely interspersed, indicating that *P. gaboni* productively infects both of these *Pan* species (Fig. 2 and Supplementary Fig. 1). The other clade represented a distinct *Laverania* lineage (B1) that included only bonobo parasites, except for a single *cytB* sequence previously identified^3^ in an eastern chimpanzee sample (PApts368) east of the Congo/Lualaba River (Fig. 1).

To determine whether B1 parasites were more widespread among eastern chimpanzees than previously recognised, we used regular and intensified PCR to screen faecal samples (*n* = 562) from nine study sites located closest to the bonobo range (Fig. 1). Although this analysis yielded twice as many *Laverania* positive samples as conventional PCR (Supplementary Table 3), none of the newly derived *cytB* sequences fell within the B1 clade (Supplementary Table 4). Instead, eastern chimpanzees were exclusively infected with *P. reichenowi* (C1), *P. gaboni* (C2) and *P. billcollinsi* (C3) (Supplementary Fig. 1). These data indicate that TL2 bonobos harbour a form of *P. gaboni* that is highly prevalent in neighbouring eastern chimpanzees as well as a second *Laverania* species that seems unique to bonobos.

**Fig. 3:**
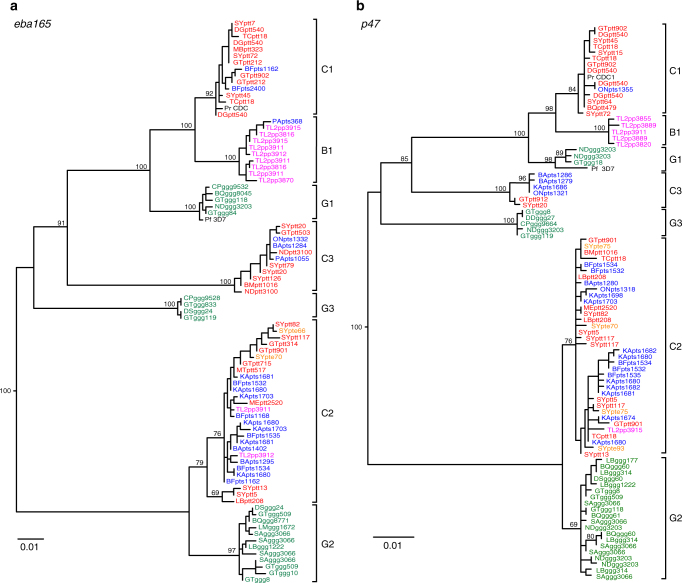
A new *Laverania* species specific for bonobos. **a**, **b** Maximum likelihood phylogenetic trees are shown for nuclear gene fragments of the **a** *erythrocyte-binding antigen 165* (*eba165;* 790 bp) and **b** the gametocyte surface protein *P47* (*p47;* 800 bp) of *Laverania* parasites. Sequences are labelled and coloured as in Fig. 2 (identical sequences from different samples are shown; identical sequences from the same sample are excluded). C1, C2 and C3 represent the chimpanzee parasites *P. reichenowi*, *P. gaboni* and *P. billcollinsi*; G1, G2 and G3 represent the gorilla parasites *P. praefalciparum, P. adleri* and *P. blacklocki* (PrCDC and Pf3D7 reference sequences are shown in black). Bonobo parasite sequences cluster within *P. gaboni* (C2) or form a new distinct clade (B1), indicating a new *Laverania* species (see text for information on the single *eba165* B1 sequence from an eastern chimpanzee). The trees were constructed using PhyML^58^ with TPM3uf+G (**a**) and GTR+G (**b**) as evolutionary models. Bootstrap values are shown for major nodes only (the scale bar represents 0.01 substitutions per site)

To characterise the newly identified bonobo parasites in other regions of their genomes, we used SGA to target additional organelle and nuclear loci for analysis (Supplementary Table 2). These included 3.4 and 3.3 kb mitochondrial DNA (mtDNA) fragments, which together span the entire mitochondrial genome; a 390 bp *caseinolytic protease M* (*clpM)* gene fragment from the apicoplast genome; and three nuclear loci, including portions of genes encoding the *erythrocyte binding antigens 165* (*eba165;* 790 bp) and *175* (*eba175;* 394 bp), and the gametocyte surface protein *P47* (*p47;* 800 bp). Phylogenetic analyses of 134 newly derived parasite sequences yielded very similar results (with respect to the clustering of parasites into major clades) in all genomic regions (Fig. 3 and Supplementary Fig. 2). Except for a single C1 *eba175* sequence indicative of a rare *P. reichenowi* infection (Supplementary Fig. 2d), all other bonobo-derived sequences fell either within *P. gaboni* or the B1 clade (Supplementary Table 2). This new clade was supported by high bootstrap values in all genomic regions analysed, except for the short *eba175* fragment. It also consistently grouped as a sister clade to *P. reichenowi*. These findings, along with the extent of genetic divergence between *P. reichenowi* and the newly identified bonobo parasite clade, argue strongly for the existence of an additional *Laverania* species that is specific for bonobos (Figs. 2 and 3 and Supplementary Figs. 1 and 2). The finding of B1 *cytB* (Fig. 2) and *eba165* (Fig. 3a) parasite sequences in a single chimpanzee faecal sample collected 280 km east of TL2 does not argue against this, since it shows that B1 parasites reached this geographic region, but failed to spread in the resident chimpanzee population (Supplementary Table 4). We propose to name the new bonobo parasite species *Plasmodium lomamiensis* sp. nov. to highlight its discovery in Lomami National Park, using faecal-derived mitochondrial, apicoplast and nuclear parasite sequences as the type material (Supplementary Data 1)^19^. Although classifying ape *Laverania* species solely on the basis of genetic information has been controversial^3,17,20^, there are no obvious alternatives given the endangered status of wild apes, the prevalence of mixed *Laverania* species infections (Supplementary Table 2) and the cryptic nature of these parasites^3,9,21^.

### TL2 bonobos also harbour non-*Laverania* parasites

**Fig. 4:**
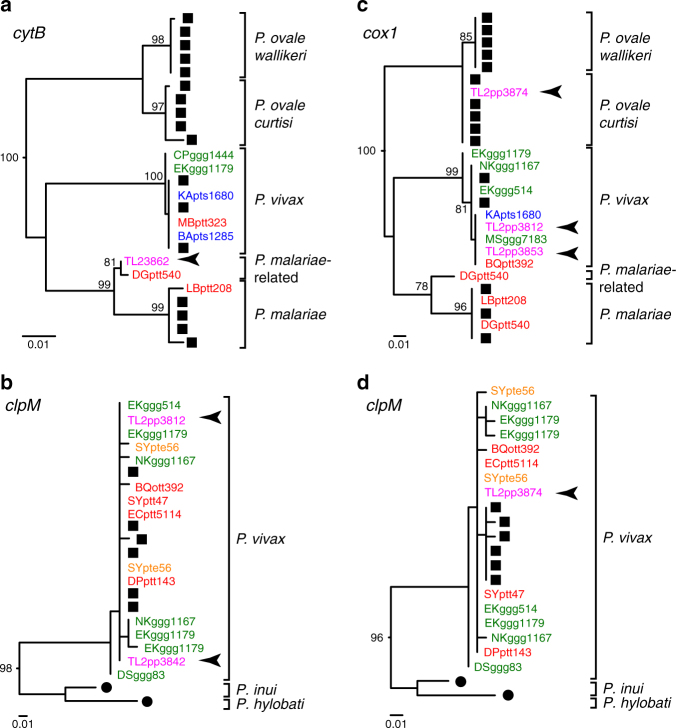
Bonobo infections with non-*Laverania* parasites. Maximum likelihood phylogenetic trees are shown for mitochondrial and apicoplast gene sequences of non-*Laverania* parasites. Ape-derived **a** *cytB* (956 bp), **b** *clpM* (327 bp), **c** *cox1* (296 bp) and **d** *clpM* (574 bp) sequences are labelled and coloured as in Fig. 2 (identical sequences from different samples are shown; identical sequences from the same sample are excluded). Human and monkey parasite reference sequences from the database are labelled by black squares and circles, respectively. Brackets indicate non-*Laverania* species, including *P. malariae, P. vivax, P. ovale curtisi* and *P. ovale wallikeri* (available sequences are too short to differentiate ape- and human-specific lineages) as well as the monkey parasites *P. inui* and *P. hylobati*. Newly identified bonobo parasite sequences are indicated by arrows, all of which are from the TL2 site. One TL2 *cytB* sequence clusters with a previously reported parasite sequence from a chimpanzee sample (DGptt540), forming a well-supported lineage that is only distantly related to human and ape *P. malariae*, and thus likely represents a new *P. malariae*-related species. The trees were constructed using PhyML^58^ with GTR+G (**a**), TRN+I (**b**, **d**) and TIM2+I (**c**) as evolutionary models. Bootstrap values over 70% are shown for major nodes only (the scale bar represents 0.01 substitutions per site)

SGA of bonobo faecal DNA also yielded rare sequences from non-*Laverania* parasites that resulted from primer cross-reactivity (Supplementary Table 2). One such *cytB* sequence clustered with a previously characterised parasite sequence from a chimpanzee sample (DGptt540), forming a well-supported lineage that was only distantly related to human and ape *P. malariae* (Fig. 4a), while two other *clpM* sequences clustered with ape and human *P. vivax* parasites (Fig. 4b). To search for additional non-*Laverania* infections, we used *P. vivax-* and *P. malariae*-specific primers to rescreen bonobo faecal samples from the BX (*n* = 1), KR (*n* = 69), LA (*n* = 199) and TL2 (*n* = 138) field sites using intensified PCR. This analysis confirmed *P. vivax* infection in one bonobo sample, and identified *P. vivax* and *P. ovale curtisi* sequences in two additional samples, all from the TL2 site (Fig. 4c). Further characterisation revealed that the *P. ovale curtisi*-positive sample also contained ape *P. vivax* sequences (Fig. 4d). Thus, of the 24 *Laverania-*positive bonobos at the TL2 site, 3 also harboured *P. malariae-*, *P. vivax-* and/or *P. ovale*-related parasites, while an additional bonobo exhibited a *P. vivax* monoinfection (Supplementary Table 2). Although the recovered sequences were too short to differentiate human- and ape-specific parasite lineages, the results show that bonobos, like chimpanzees and gorillas, are frequently infected with multiple *Laverania* and non-*Laverania* species^1,5,7^. However, unlike chimpanzees and gorillas, bonobos harbour these parasites in only one particular part of their range.

### The Lomami River is not a barrier to malaria transmission

**Fig. 5:**
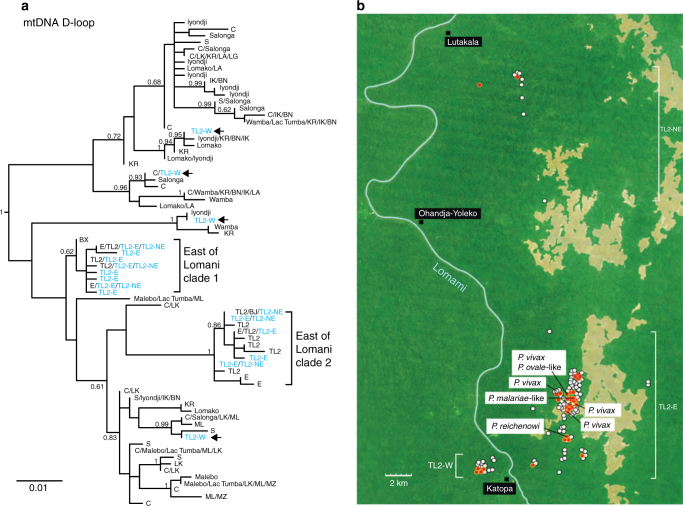
The Lomami River is not a barrier to *Laverania* parasite transmission. **a** Maximum likelihood phylogenetic tree of bonobo mitochondrial (D-loop) sequences. Haplotypes are labelled by field site (see Fig. 1 and refs. ^7,22,23,77,^ for their geographic location and code designation), with those identified at multiple field sites indicated (e.g., C/Wamba/KR/BN/IK/LA). Newly derived haplotypes from the TL2 site are shown in blue (previously reported mtDNA sequences are shown in black)^22,23,77^. Brackets highlight two clades that are exclusively comprised of mtDNA sequences from bonobos sampled east of the Lomami River. TL2 haplotypes that do not fall within these clades (denoted by arrows) were all sampled west of the Lomami River (TL2-W). The tree was constructed using PhyML^58^ with HKY+G as the evolutionary model. Bayesian posterior probability values ≥ 0.6 are shown (the scale bar represents 0.01 substitutions per site). **b** Locations of individual bonobo faecal samples collected at the TL2 site. Sampling locations west (TL2-W) and east (TL2-E and TL2-NE) of the Lomami River were plotted using GPS coordinates, with red and white dots indicating *Laverania* parasite positive and negative specimens, respectively. Samples that contained *P. reichenowi*, *P. malariae*-like*, P. vivax*-like and *P. ovale*-like parasites are also indicated. Forested areas are shown in green, while savannas are depicted in brown. The Lomami River is shown in blue. Local villages are denoted by black squares. The scale bar indicates 2 km

Analysing mtDNA sequences to determine the population structure of wild bonobo populations, two previous studies reported that the Lomami River, but not other tributaries of the Congo River, represents a geographical barrier to bonobo gene flow^22,23^. We thus considered the possibility that bonobos in the western and central regions of the DRC had acquired a malaria protective trait that had not spread to bonobo populations east of the Lomami River. To investigate this, we subjected *Plasmodium*-positive and -negative samples from TL2 to the same host mtDNA analysis (Supplementary Data 2) and compared the resulting haplotypes to all previously reported bonobo mtDNA sequences (Fig. 5a and Supplementary Fig. 3). Phylogenetic analysis showed that most of the newly derived mtDNA sequences from TL2 (blue) fell into two clades that were exclusively comprised of sequences from bonobos sampled east of the Lomami River (Supplementary Fig. 3b)^22,23^. However, 4 new TL2 haplotypes representing 15 faecal samples, including 4 *Laverania*-positive specimens, did not fall within these two 'eastern' clades (arrows in Fig. 5a and Supplementary Fig. 3a). Analysis of their GPS coordinates revealed that they were all collected west (TL2-W) of the Lomami River (Fig. 5b). These results thus confirm and extend previous findings showing that bonobos east of the Lomami River represent a genetically (at least matrilineally) isolated population^22,23^. However, this isolation does not explain the geographic restriction of bonobo malaria, since *Laverania*-positive individuals were found on both sides of the Lomami River. Although it remains unknown how far the *Plasmodium* endemic area extends beyond TL2 in the eastern Congo, it seems clear that the Lomami River itself does not represent a barrier to malaria transmission.

### Climate does not explain the distribution of bonobo malaria

Because climatic factors such as ambient temperature and rainfall are known to influence malaria transmission in humans^24–26^, we asked whether seasonal differences in *Plasmodium* prevalence could explain the lack of parasite detection at the majority of bonobo study sites. Comparison of sample dates across all field sites revealed no obvious association between faecal parasite positivity and the month of specimen collection (Table 2). For example, samples collected in November and December at the TL2 site included a large fraction of malaria-positive specimens, but this was not the case for samples collected during these same months at the IK, KR and LA field sites. To examine the impact of climatic variation on bonobo parasite detection more directly, we used a statistical model shown to be strongly predictive of spatiotemporal variation in *Laverania* infection among wild-living chimpanzees (Erik Scully, unpublished results). This model, which was parameterised using PCR screening data from 2436 chimpanzee faecal samples collected at 55 locations across equatorial Africa^7^, showed that ambient temperature, daily temperature fluctuations and forest cover, but not rainfall, each influenced the probability of *Laverania* detection.

**Table 2:** Seasonal variation in parasite prevalence does not explain the geographic restriction of bonobo malaria

Site	**Sample collection** ^**a**^												***Laverania cytB***		**Model predictions**		
	Jan	Feb	Mar	Apr	May	Jun	Jul	Aug	Sep	Oct	Nov	Dec	Pos	Total	Mean^b^	Significance cutoff^c^	*p*-value^d^
BN							49	35					0	84	28.2	17	<10^–6^
BX										1			0	1	0.4	0	1.0
BJ						2							0	2	0.7	0	1.0
IK			107	22	46	4	85			7	61	121	0	453	147	121	<10^–6^
KR	5								4	9 (1)	13	33	1	64	25.1	15	<10^−6^
LG	11											14	0	25	6.9	2	0.003
LA								12	43	30	73	41	0	199	54.5	39	<10^−6^
LK			4	6	28								0	38	14.2	7	<10^−6^
ML			56	86									0	142	4.1	0	0.176
MZ				5		49	9	16	57	25			0	161	22.6	12	<10^−6^
TL2	8 (4)									8	64 (18)	33 (5)	27	113	39.5	27	0.083

^a^Only samples with known collection dates and GPS coordinates, for which land surface temperature and forest cover data could also be obtained, were included in the climate analysis (sample numbers are thus lower than in Table 1). Numbers in brackets indicate *Laverania cytB-*positive samples

^b^The expected mean number of positive samples predicted by the climate model

^c^The significance cutoff corresponds to the 0.45% (5%, with Bonferroni correction for 11 tests) of the Poisson binomial distribution for the probabilities predicted by the climate model. Values of 0 indicate that a greater sample size is necessary to confidently reject the climate model

^d^A significant Bonferroni adjusted *p*-value (*p *< 0.05) indicates that climate can be rejected as the explanation for rare or absent *Plasmodium* infections at any particular sampling site

Using only specimens with known sampling dates and GPS coordinates for which land surface temperature and forest cover data were also available (Supplementary Table 5), we estimated the probability of *Laverania* infection for each of the 11 bonobo field sites. Assuming similar climatic influences on chimpanzee and bonobo parasite development and transmission, this analysis showed that at seven sites for which a sufficiently large number of samples were available, bonobos were significantly less frequently *Laverania* infected than predicted by the climate model. For the BN, IK, LA, LK and MZ sites, the model predicted a less than one in a million probability that a positive sample would not be detected if bonobos at these sites exhibited similar infection patterns as chimpanzees. Moreover, for the KR site, where only one sample was *Laverania* positive, seasonal variation could not explain this very low detection rate (Table 2). The rate of parasite detection at the TL2 site, where 27 of 113 samples with climate data were positive, was lower than, but not significantly different from, that predicted for a chimpanzee study site with similar ecological conditions. The very small sample sizes at BJ and BX sites lacked statistical power to detect differences, and the low predicted probability of infection at the ML site indicated that more sampling during months of higher infection probabilities would be necessary to confidently reject the climate model. Nevertheless, our sampling density at most sites was sufficient to conclude that the scarcity of infection in bonobos was not caused by biased sampling during seasonal troughs (Table 2). Thus, it appears that seasonal or climatic variation in parasite prevalence can be excluded as an explanation for the observed geographic restriction of bonobo *Plasmodium* infections.

### Bonobo diet is not associated with faecal parasite detection

Wild apes consume a variety of plants, fruits, barks and piths, some of which have been reported to have antimalarial activity^27–29^. We thus asked whether our inability to detect *Plasmodium* infections at most bonobo field sites was due to the presence of certain plants, which upon ingestion would reduce parasite titres below the limits of faecal detection. To examine this possibility, we selected a subset of *Laverania*-positive (*n* = 18) and -negative (*n* = 51) bonobo faecal samples from endemic (TL2) and non-endemic (KR, IK, LG, LK) field sites, and characterised their plant content by targeting two regions of the chloroplast genome for high-throughput sequencing (Supplementary Table 6). These comprised a 500 bp fragment of the *rbcL* gene and a 750 bp fragment of the *matK* gene, both of which have been used as barcodes to identify land plants^30–32^, including in stool samples from endangered species^33^. *Laverania-*positive (*n* = 14) and -negative (*n* = 15) chimpanzee faecal samples were analysed for control (Supplementary Table 6).

**Fig. 6:**
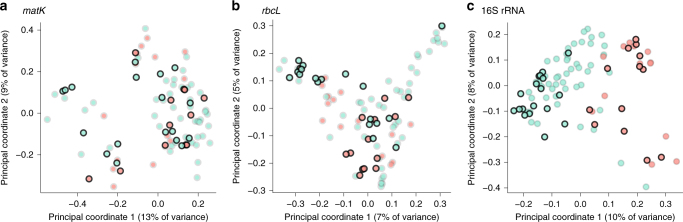
*Laverania* infection of bonobos is not associated with particular faecal plant or microbiome constituents. A principal component analysis of unweighted UniFrac distances was used to visualise compositional differences of **a**,** b** plant (*matK* and *rbcL*) and **c** bacterial (16S rRNA) constituents in *Laverania*-positive (dark border) and -negative (light border) faecal samples from bonobos (blue) and chimpanzees (pink). The sample positions (shown for the first two components) do not indicate separate clustering of *Laverania*-positive and -negative samples

Samples were sequenced to a mean depth of 16,054 *matK* and 21,995 *rbcL* paired-end reads, which were clustered into operational taxonomic units (OTUs) and assigned to taxonomic groups using a custom *matK* and *rbcL* reference database (Supplementary Fig. 4). Using a permutational multivariate analysis of variance (PERMANOVA) to compare unweighted UniFrac distances^34^ as a measure of large-scale differences in plant composition, we found small differences between faecal samples from bonobos and chimpanzees (*matK*: 2.0% of variance, *p* = 0.003; *rbcL*: 2.8% of variance, *p* < 10^−6^), but substantial differences between faecal samples from different study sites (*matK*: 19.8% of variance, *p* < 10^−6^; *rbcL*: 18.6% of variance, *p* < 10^−6^). However, no significant differences were observed between *Laverania*-positive and -negative faecal samples (*matK*: 1.2% of variance, *p* = 0.18; *rbcL*: 0.8% of variance, *p* = 0.71), suggesting that the lack of parasite detection was not associated with the abundance of certain plant phyla in the diet (Fig. 6a, b and Supplementary Fig. 5).

We also compiled a list of 466 African plant species (Supplementary Table 7), which have been reported to have potential antimalarial activity^29,35,36^, and looked for related *matK* and *rbcL* sequences in bonobo faecal samples from endemic and nonendemic field sites. Although a BLAST search identified 65 *matK* and 490 *rbcL* OTUs that shared 95% sequence identity with 3 and 17 of these putative antimalarial species, respectively, none was significantly more abundant at field sites where *Laverania* infections were absent (Supplementary Fig. 6). Similar results were obtained when the remaining plant OTUs were compared between endemic and nonendemic bonobo field sites. Finally, no compositional differences were observed in the plant content of *Laverania-*positive and -negative chimpanzee faecal samples (Fig. 6a, b). Although these analyses provide only a snapshot of bonobo and chimpanzee plant diet, they failed to identify an association between particular plant constituents and parasite detection in faecal samples.

### The faecal microbiome does not predict *Laverania* infection

*Plasmodium* infections have been reported to influence the bacterial communities in the gut, with certain parasites causing intestinal dysbiosis^37^ and certain gut microbiota enhancing the host’s anti-parasite immune responses^38^. To examine potential interactions between the faecal microbiome and *Laverania* infection in bonobos, we used the same samples selected for plant analyses (Supplementary Table 6) for bacterial 16S rRNA sequencing (*Laverania*-positive and -negative chimpanzee samples again served as a control). Samples were sequenced to a mean depth of 65,132 reads, which were clustered into OTUs and assigned to taxonomic groups (Supplementary Fig. 7a). Examining Shannon diversity as a marker of dysbiotic outgrowth or loss of bacterial taxa, we failed to find significant differences in within-sample (alpha) diversity between specimens from *Laverania*-positive and -negative bonobos (or chimpanzees), or between specimens from endemic (TL2) and nonendemic (KR, IK, LG, LK) field sites (Supplementary Fig. 8a). Using unweighted UniFrac distance to compare between-sample (beta) diversity^34^, we found that as previously reported^39^ bonobo and chimpanzee faecal microbiomes differed in their bacterial composition (Fig. 6c and Supplementary Figs. 7b and 8b). Samples from the same field site were also often compositionally more similar to each other than to samples from other field sites (Supplementary Fig. 8b and c). Using PERMANOVA to examine the sources of this variation, we found that ape species accounted for 7.4% (*p* < 10^−6^), study site for 19.3% (*p* < 10^−6^) and *Laverania* positivity for 1.2% of the variance (*p* = 0.043), respectively. Considering only chimpanzee samples, study site accounted for 17.8% (*p* = 0.000018) and *Laverania* positivity for 4.0% of variance (*p* = 0.25). Comparing only samples from TL2 bonobos, differences among three sample locations (Fig. 5b) accounted for 14.6% (*p* < 10^−6^) and *Laverania* infection for 4.5% of variance (*p* = 0.0023). Thus, there was a small but significant compositional difference between the faecal microbiome of *Laverania-* positive and -negative bonobos at TL2 (the lack of significance in chimpanzees may be due to a smaller sample size).

Using Wilcoxon rank sum tests to look for OTUs that were driving these differences, we found one assigned to the family Ruminococcaceae that was significantly depleted, and two others assigned to family Lachnospiraceae and *Prevotella copri* that were significantly enriched in *Laverania*-positive TL2 bonobo samples (Supplementary Fig. 9). However, comparing samples from TL2 to nonendemic sites did not yield significantly higher UniFrac distance values than comparing samples between these nonendemic sites (Supplementary Fig. 8b). Thus, while the abundance of some bacterial taxa differed slightly between *Laverania*-positive and -negative bonobos at TL2, compositional differences between samples from TL2 and nonendemic sites were no greater than expected between any two random sites, thus failing to provide a microbial signature of *Laverania* infection for that site.

## Discussion

A complete account of *Plasmodium* infections in wild African apes, including their host associations, prevalence, geographic distribution and vector preferences, is critical for understanding the origins of human malaria and gauging future zoonotic risks. Previous studies documented numerous *Plasmodium* species in wild chimpanzees and gorillas, but failed to find similar infections in wild bonobos^1,2^. Here, we show that bonobos harbour a multitude of *Plasmodium* species, although endemic infection is limited to only a small part of their range east of the Lomami River. Analyses of climate data and parasite seasonality, as well as host characteristics, including bonobo population structure, plant consumption and faecal microbiome composition, failed to provide an explanation for this geographic restriction. Thus, other factors must be responsible for the uneven distribution of bonobo *Plasmodium* infections, including the possibility of a protective mutation that has not spread east of the Lomami River.

Studies in Asia have shown that both species richness and prevalence of primate malarias are closely linked to the habitat of forest-dwelling *Anopheles*, rather than the distribution of the primates themselves^10,12^. Thus, factors that negatively impact the breeding conditions, development and distribution of transmitting vectors may be responsible, at least in part, for the absence of *Plasmodium* infections at most bonobo sites. Ecological factors may also influence bonobo density or other behaviours that affect vector exposure. For example, captive orangutans, which live in higher group densities than their wild counterparts, also have higher rates of *Plasmodium* infection^11^. Finally, it is conceivable that bonobos at the *Plasmodium-*negative sites carry other infections that induce cross-protective immunity or compete for the same resources^40^. Bonobos are clearly susceptible to a variety of *Plasmodium* species. Thus, examining why neither *Laverania* nor non-*Laverania* infections are sustained throughout much of the bonobo range may identify new drivers of vector dynamics or other transmission risks that could aid malaria eradication efforts in humans.

Although diagnostic PCR of matched blood and stool samples from *Plasmodium-*infected captive macaques indicated faecal detection rates of up to 96%^41^, parasite detection in wild primates is much less sensitive due to widely varying sample quality. While the new bonobo *Laverania* infections were identified by conventional PCR (Table 1), we reasoned that multiple PCR replicates would increase the chances of parasite detection and thus the number of sequences for phylogenetic analyses. This was indeed the case since 18 (of 191) bonobo and 46 (of 517) chimpanzee samples identified as *Plasmodium* negative by conventional PCR were subsequently found parasite positive by intensified PCR (Table 1 and Supplementary Table 3). Using these results (Supplementary Table 1), we estimated the sensitivity of a single PCR replicate to be 16.7% (95% confidence interval (CI): 12.9–20.8), and the sensitivity of 8 and 10 replicates to be 76.8% and 83.9%, respectively (see Methods). Although labour intensive, costly and prone to contamination, intensified PCR is the method of choice for samples with low parasite levels, such as partially degraded specimens from remote field sites, since its increased sensitivity can detect rare, and possibly even new, *Plasmodium* species.

Phylogenetic analyses of over 3000 mitochondrial, apicoplast and nuclear parasite sequences from chimpanzee and gorilla faecal samples have consistently pointed to the existence of six ape *Laverania* species^1,3–6^. Here, we propose a seventh species, *P. lomamiensis*, on the basis of partial organelle and nuclear gene sequences derived from faecal DNA. Unlike other ape parasites, this species seems to be bonobo specific and geographically restricted to the Lomami River basin. Although a traditional full taxonomic description might seem preferable, the highly endangered status of wild bonobos precludes blood collection. Moreover, the cryptic nature of *Laverania* parasites as well as the high frequency of mixed species infections renders a morphological description uninformative. Indeed, *P. gaboni* and *P. reichenowi*, which are morphologically indistinguishable from *P. falciparum*, were only recently shown by full-length genome sequencing to represent distinct, non-recombining species^9,42^, and the same type of analysis uncovered that human *P. ovale* comprises not one but two sympatric parasite species^43,44^. Thus, a true description of cryptic *Plasmodium* species must come from multilocus genetic analysis. Although we used single template amplification to derive high-quality organelle and nuclear sequences of multiple *P. lomamiensis* strains, the number of loci and samples is still limited. As DNA-only species taxonomy is gaining wider acceptance^19^, it will be interesting to determine how much genetic information is necessary to reliably classify *P. lomamiensis* and other putative *Plasmodium* species.

The newly identified bonobo parasites prompt speculation about the causes and timescale of *Laverania* diversification. When the only *Laverania* species characterised were *P. falciparum* and *P. reichenowi*, it was widely assumed that these two species had co-diverged with their hosts^45,46^, placing their common ancestor at the same time as the common ancestor of humans and chimpanzees around 6–7 Mya^47^. This hypothesis was undermined by the finding of additional *Laverania* species, in particular the discovery that *P. falciparum* resulted from the recent host switch of the gorilla parasite *P. praefalciparum* ^1^. However, *P. praefalciparum* and *P. reichenowi* could have co-diverged with the ancestors of gorillas and chimpanzees. The phylogenetic position (Figs. 2 and 3) of the newly described bonobo parasite, *P. lomamiensis* (B1), which is more closely related to *P. reichenowi* (C1) than to *P. praefalciparum* (G1), provides a triad of parasite species with the same relationships as their hosts (Supplementary Fig. 10). It is thus tempting to speculate that this clade arose through host–parasite co-divergence. Under this scenario, the common ancestor of *P. reichenowi* and *P. praefalciparum* would have existed approximately 8–9 Mya^47^, an estimate that is 2 to 4 times older than some have concluded from molecular clock analyses for the equivalent divergence of *P. reichenowi* and *P. falciparum* ^48,49^. *P. reichenowi* and *P. lomamiensis* would have diverged around 2 Mya^47^. Molecular clocks for *Laverania* species may not be very precise: for example, *P. reichenowi* and *P. praefalciparum* are clearly not 4 times more divergent than *P. reichenowi* and *P. lomamiensis* (Figs. 2 and 3). However, given that *P. reichenowi* and *P. gaboni* are about 3 times more divergent than *P. reichenowi* and *P. praefalciparum* ^9^, it is possible that the common ancestor of the entire *Laverania* clade existed around 25–30 Mya.

The co-divergence scenario also predicts that the ancestor of today’s bonobos was infected with the ancestor of *P. lomamiensis*, which was subsequently lost from most bonobo populations. The Congo River, which forms the boundary between the ranges of chimpanzees and bonobos (Fig. 1), is thought to have existed since long before the divergences among African apes^50^; yet, somehow the ancestor of bonobos reached the left bank of the Congo. This may have happened during one of several periods of aridity when river levels might have been low enough to permit the crossing in the northeast of the current bonobo range^50^. Furthermore, mitochondrial DNA analyses suggest that there may have been an early population split between the ancestors of bonobos now found east and west of the Lomami River^51^. Thus, the loss of *P. lomamiensis* from western populations may have occurred early in bonobo history. It should be noted that the infection status of bonobos at sites other than TL2 east of the Lomami (e.g., BJ and BX; Fig. 1) remains unknown, because too few samples have been collected. However, bonobos immediately west of the Lomami at TL2 must have reacquired *P. lomamiensis*, indicating that the river is not a barrier to mosquitoes from the east, and that western bonobos as a whole do not share a genetically based resistance to infection. The co-divergence scenario also implies that a related parasite was lost from the human lineage, which might have been due to an early human population bottleneck, an ancestral hunter–gatherer lifestyle^52^ and/or the loss of the gene that synthesises *N*-glycolylneuraminic acid (Neu5Gc), which may have affected the ability of the parasite to infect human erythrocytes^53^.

The extent of divergence (Figs. 2 and 3) between *P. gaboni* (C2) and *P. adleri* (G2) is similar to that between *P. reichenowi* and *P. praefalciparum*, suggesting that the former pair may also have co-diverged with their hosts. Within the C2/G2 clade there is again no human parasite species, and the only bonobo parasites in this lineage clearly reflect recent transmissions of *P. gaboni* from eastern chimpanzees, rather than co-divergence. In this case, the loss of a putative B2 lineage from bonobos is not surprising given the loss of *P. lomamiensis* from most of the bonobo range. The lack of close relatives of *P. billcollinsi* (C3) and *P. blacklocki* (G3) would also be indicative of past losses of parasite lineages from particular ape hosts. Although the processes that contributed to the emergence of today’s *Laverania* lineages remain unknown, it seems likely that both co-divergence and cross-species transmission events shaped their evolutionary history, as has been observed for many other pathogens.

One characteristic of *Laverania* parasites infecting wild apes is their highly specific host tropism. This species specificity is not shared by non-*Laverania* parasites, such as *P. vivax*, which infects bonobos, humans, chimpanzees and gorillas^2,54^. However, even within the *Laverania* subgenus, host specificity is not absolute. Bonobos at TL2 are commonly infected with the chimpanzee parasite *P. gaboni*, which appears to have crossed the Lualaba River on multiple occasions (Fig. 2). Bonobos are also susceptible to the chimpanzee parasite *P. reichenowi* (Supplementary Fig. 2d), while eastern chimpanzees appear susceptible to the bonobo parasite *P. lomamiensis* (Figs. 2 and 3), although both of these cross-infections appear to reflect rare events that fail to result in onward transmission. Thus, on the one hand, *Laverania* species are extremely host specific, implying strong barriers to cross-species transmission, and on the other hand there is evidence that on occasion these barriers can be overcome. Given the very close genetic relationship of chimpanzees and bonobos, examples of cross-infections are perhaps not surprising. However, the finding that in captive settings bonobos can become infected with human *P. falciparum* ^17^, while chimpanzees can harbour gorilla parasites and vice versa^55^, indicates that *Laverania* host specificity goes beyond incompatibilities of receptor–ligand interactions during erythrocyte invasion^56^. While ape *Laverania* parasites have not yet been detected in humans^8,57^, it seems clear that the mechanisms governing host specificity are complex and that some barriers are more readily surmountable than others. Given the new bonobo data, it will be critical to determine exactly how *P. praefalciparum* was able to jump the species barrier to humans, in order to determine what might enable one of the other ape *Laverania* parasites to do the same.

## Methods

### Ape samples

Faecal samples from wild-living bonobos and eastern chimpanzees were obtained from existing specimen banks, or were newly collected at previously reported^1–3^ as well as new study sites (LG, BX, MZ) in the DRC (Fig. 1). While all available bonobo samples (*n* = 1556) were analysed, eastern chimpanzee specimens (*n* = 580) were selected from 9 field sites most proximal to the bonobo range. All samples were obtained noninvasively from apes in remote forest areas, preserved (1:1 vol/vol) in RNA*later*, transported at ambient temperatures and stored at −80 °C. Faecal DNA was extracted using the QIAamp Stool DNA mini kit (Qiagen) and all specimens were subjected to host mtDNA analysis to confirm their species and subspecies origin^1–3^. The latter analysis also gave an indication of sample quality, which confirmed that samples from *Laverania*-negative field sites were not any more degraded than samples from TL2. For the KR, LK and TL2 field sites, the number of sampled individuals has previously been determined by microsatellite analyses^2^. All samples were obtained with approval from the Ministries of Scientific Research and Technology, the Department of Ecology and Management of Plant and Animal Resources of the University of Kisangani, the Ministries of Health and Environment, and the National Ethics Committee in the DRC, and shipped in compliance with Convention on International Trade in Endangered Species of Wild Fauna and Flora regulations and country-specific import and export permits.

### Conventional and intensified PCR

Bonobo and chimpanzee faecal samples were first screened for *Laverania* parasites by conventional (diagnostic) PCR, targeting a 956 bp mitochondrial *cytB* fragment using primers DW2 (5′-TAATGCCTAGACGTATTCCTGATTATCCAG-3′) and DW4 (5′-TGTTTGCTTGGGAGCTGTAATCATAATGTG-3′) in the first round, and Pfcytb1 (5′-CTCTATTAATTTAGTTAAAGCACA-3′) and PLAS2a (5′-GTGGTAATTGACATCCWATCC-3′) in the second round of PCR as previously described^1^. Since this approach tests only a single aliquot of each faecal DNA, we reasoned that parasites present in low concentrations may have been missed. To increase the sensitivity of parasite detection, we thus tested 8 to 10 aliquots of the same DNA preparation using the same primers and amplification conditions. To guard against false positives, only samples that were negative by conventional PCR were subjected to the intensified PCR screening. Assuming that PCR amplification was 100% specific, that all PCR reactions were independent with a fixed probability of detecting a positive sample, and that samples found negative in all PCR replicates could still be *Plasmodium* positive, we expected that the number of positive PCR reactions for a faecal sample would follow a zero-truncated binomial distribution and that the likelihood of the data would be the product over all samples with a positive reaction for this intensified PCR approach. Therefore, we used numerical optimisation to find the maximum likelihood estimate of sensitivity for these data and determined 95% CIs using likelihood ratios. Using intensified PCR data from both bonobo and chimpanzee samples (Supplementary Table 1), we estimated the sensitivity of a single PCR replicate to be 16.7% (95% CI: 12.9–20.8), and thus the sensitivity of 8 and 10 independent PCR reactions to be 76.8% and 83.9%, respectively. Separate analyses of chimpanzee and bonobo samples yielded similar sensitivity estimates for single PCR replicates: 17.5% (95% CI: 13.0–22.5) for eastern chimpanzees and 14.6% (95% CI: 8.47–22.3) for bonobos. Intensified PCR was also used to screen 199 bonobo faecal samples from the TL2, KR and LA field sites for non-*Laverania* infections using parasite-specific primer sets. *P. vivax* primers targeted a 296 bp *cox1* fragment using Pv2768p (5′-GTATGGATCGAATCTTACTTATTC-3′) and Pv3287n (5′-AATACCAGATACTAAAAGACCAACAATGATA-3′) in the first round, and Pv2856p (5′-CTTATTACAAATTGCAATCATAAAACTTTAGGT-3′) and Pv3185n (5′-TCCTCCAAATTCTGCTGCTG TAGATAAAATG-3′) in the second round of PCR as previously described^8^. *P. malariae* specific primers targeted a 600 bp mitochondrial *cytB* fragment using Pm4659p (5′-ATTTATTATCTTCAATTCCAGCACTT-3′) and Pm5501n (5′-GCATGTTAACTCGATAAATACTAA-3′) in the first round, and Pm4740p (5′-ATTACATTTTATACTTCCATTTGTTGC-3′) and Pm5369n (5′-TTCAGAAATATCGTCTTATCGTAGC-3′) in the second round of PCR. *P. vivax*-specific primers detected both *P. vivax* and *P. ovale*, while *P. malariae*-specific primers amplified only positive control samples (one *P. malariae-*positive sample was detected due to the cross-reactivity of regular *cytB* primers; Fig. 4a). All amplicons were sequenced directly without interim cloning.

### Single genome amplification

To derive *Plasmodium* sequences devoid of PCR-induced errors, all PCR-positive bonobo and chimpanzee faecal samples were subjected to SGA as previously described^1,2,18^. According to the Poisson distribution, the DNA dilution that yields PCR products in no more than 30% of wells contains one amplifiable template per positive reaction more than 80% of the time. Faecal DNA was thus end point diluted in 96-well plates, and the dilution that yielded <30%-positive wells was used to generate single template-derived sequences. For *Laverania-*positive bonobo samples, mitochondrial (*cytB*; 3.4 and 3.3 kb mitochondrial half genomes), apicoplast (*clpM*) and nuclear (*eba165, eba175* and *p47*) gene regions were amplified using previously reported primer sets and amplification conditions (Supplementary Table 2)^1–3,8,56^. For *Laverania*-positive chimpanzee samples, only the 956 bp mitochondrial *cytB* fragment was amplified (Supplementary Table 4). One bonobo faecal sample (TL2.3874) positive for *P. ovale curtisi* by conventional PCR also yielded a *P. vivax-*specific 574 bp apicoplast *clpM* fragment when subjected to SGA analysis.

### Phylogenetic analyses

Sequences were aligned using CLUSTAL W (version 2.1), visually inspected and regions that could not be unambiguously aligned were removed from subsequent analyses. Maximum likelihood phylogenetic trees and bootstrap support were estimated using PhyML (version 3.0)^58^, which infers evolutionary model parameters and phylograms concurrently. Evolutionary models were selected using jModelTest (version 2.1.4)^59^. Bayesian posterior probabilities were determined using MrBayes (version 3.2.4)^60^ using two simultaneous independent analyses with a 25% burn-in. Convergence was determined when the average deviation of split frequencies was <0.01.

### Climate model of ape *Laverania* infection

To evaluate whether the absence or low prevalence of *Laverania* infection at most bonobo sampling sites could be explained by seasonal variation in parasite transmission, we developed a logistic generalised linear mixed model to infer the probability of parasite detection for each sample relative to the climatic variables observed at the time of specimen collection. Briefly, this model, which incorporates mean ambient temperature (AT), daily temperature variation (TV) and percent forest cover (FC), was parameterised using 2436 chimpanzee faecal samples from 55 sampling sites across equatorial Africa^7^ and found to be strongly predictive of *Laverania* infection in wild chimpanzees (Erik Scully, unpublished results). Assuming similar relationships between climatic variables and infection probability in chimpanzees and bonobos, and including only samples for which climate data were available, we inferred the predicted probability of *Laverania* infection for each bonobo sample using the equation:

$${\rm Predicted\,Probability} = \frac{1}{{1 + e^{ - \left( {{\rm intercept} + 0.355 \times {\rm AT} - 0.164 \times {\rm AT}^2 + 0.032 \times {\rm FC} + 0.208 \times {\rm TV}} \right)}}}$$

where intercept is −2.538 for samples screened using conventional PCR (i.e., one replicate) and −1.374 for those screened using intensive PCR (i.e., 8–10 replicates), and AT, TV and FC are each corrected by subtracting the means of the chimpanzee data set (23.4 for AT, 9.1 for TV and 77.2 for FC). For each bonobo sample, we used MODerate Resolution Imaging Spectroradiometer (MODIS) and daytime and night-time land surface temperature (LST) data sets^61,62^ in one-day temporal resolution (MOD11A1) after applying the minimum/maximum air temperature transformations as previously described^24^ to derive (1) the mean ambient air temperature and (2) the mean daily air temperature fluctuation. Each of the temperature variables was calculated as the average of LST measurements taken during the period 30 days prior to sample collection. Forest cover data were extracted from high-resolution global maps as previously described^63^. For each sampling site, the mean and ranges of these ecological variables are summarised in Supplementary Table 5. Assuming that each specimen is independent and has a probability of detected infection as assigned by the climate model, the number of positives observed at a given site will be a sum of Bernoulli variables with varying probabilities and thus should follow the Poisson binomial distribution^64^. We calculated the cumulative probability of seeing less than or equal the observed number of positive samples^64^ given the set of climate estimates for each site to generate *p*-values and used Bonferroni correction to account for multiple comparisons. A low *p*-value indicates that climatic variation is very unlikely to account for the observed scarcity of infection.

### Characterisation of faecal plant composition

Chloroplast ribulose bisphosphate carboxylase large chain (*rbcL*) and maturase K (*matK*) gene regions are widely used as bar codes for land plants^30–32^ and were thus selected to characterise plant components in *Laverania*-positive and negative bonobo (*n* = 78) and chimpanzee (*n* = 20) faecal samples (Supplementary Table 6). Faecal DNA was extracted using the PowerSoil-htp 96 Well Soil DNA Isolation Kit (MO BIO Laboratories, Carlsbad, CA, USA). We modified *rbcL* primers previously reported to have high plant discriminatory ability^32^ for MiSeq sequencing by adding an Illumina adapter (underlined). These included rbcLbF (5′-AGACCTWTTTGAAGAAGGTTCWGT-3′) and rbcLbR (5′-TCGGTYAGAGCRGGCATRTGCCA-3′) for the first round of PCR, and R1_rbcL634F (5′-TCGTCGGCAGCGTCAGATGTGTATAAGAGACAGATGCGTTGGAGAGACCGTTTC-3′) and R2_rbcLbR (5′-GTCTCGTGGGCTCGGAGATGTGTATAAGAGACAGTCGGTYAGAGCRGGCATRTGCCA-3′) for the second round of PCR. We also modified *matK* primers recently improved to achieve high PCR success rates^31^ in a similar fashion, using matK390F (5′-CGATCTATTCATTCAATATTTC-3′) and matK1326R (5′-TCTAGCACACGAAAGTCGAAGT-3′) in the first round of PCR, and R1_matK472F (5′-TCGTCGGCAGCGTCAGATGTGTATAAGAGACAGCCCRTYCATCTGGAAATCTTGGTTC-3′) and R2_matK1248R (5′-GTCTCGTGGGCTCGGAGATGTGTATAAGAGACAGGCTRTRATAATGAGAAA GATTTCTGC-3′) in the second round of PCR.

Amplification for both *rbcL* and *matK* gene regions was performed using 2.5 μl of sample DNA in a 25 μl reaction volume containing 0.5 μl dNTPs (10 mM of each dNTP), 10 pmol of each first round primer, 2.5 μl PCR buffer, 0.1 μl BSA solution (50 μg/ml), and 0.25 μl Expand Long Template enzyme mix (Expand Long Template PCR System) for the first round of PCR. Cycling conditions included an initial denature step of 2 min at 94 °C, followed by 15 cycles of denaturation (94 °C, 10 s), annealing (45 °C, 30 s) and elongation (68 °C, 1 min), followed by 25 cycles of denaturation (94 °C, 10 s), annealing (48 °C, 30 s) and elongation (68 °C, 1 min; with 10 s increments for each successive cycle), followed by a final elongation step of 10 min at 68 °C. For the second round PCR, 2 μl of the first round product was used in 25 μl reaction volume. Cycling conditions included an initial denature step of 2 min at 94 °C, followed by 25 cycles of denaturation (94 °C, 10 s), annealing (52 °C, 30 s) and elongation (68 °C, 1 min), followed by a final elongation step of 10 min at 68** °**C. For each faecal sample, *rbcL* and *matK* gene regions were amplified in duplicate, the products were pooled, purified using QIAquick Gel Extraction Kit and sequenced using the Illumina Miseq v2 (500 cycle).

Sequence reads were separated by barcode, quality filtered for an expected number of errors <1 and an exact match to primer sequences, and the 5′ and 3′ reads of each pair were concatenated after trimming off primer sequences. OTUs were formed using Swarm^65^, and OTUs containing only a single read discarded. Representative sequences of each OTU were aligned using MAFFT^66^ and a phylogenetic tree was inferred using FastTree^67^. To create a database for taxonomic assignment, all reads matching the search terms '*matK*' or '*rbcL'* were downloaded from the European Nucleotide Archive and indexed in a BLAST database. This database was searched using a representative sequence from each OTU, and taxonomy was assigned as the most specific taxonomic rank shared by all BLAST hits with a total bit score within 98% of the best hit. Samples with <5000 reads were removed from the analysis.

### Characterisation of faecal bacterial constituents

The same faecal DNA samples used for plant analyses were also subjected to bacterial 16S rRNA gene sequencing (Supplementary Table 6). The 16S rRNA gene amplification was performed as previously described^68^, using 5 μl of faecal DNA, the AccuPrime Taq DNA Polymerase High Fidelity System (Thermo Fisher), and V1V2 region primers containing Illumina adapters, barcode and linker regions. Each faecal sample was amplified in four independent reactions, with the products pooled and purified using AMPure XP beads (Beckman Coulter) before sequencing using Illumina MiSeq v2 (500 cycle). Sequences were separated by barcode, and paired reads were merged using bbmerge^69^. Reads were clustered into OTUs using a cutoff of 97% identity and taxonomically assigned using QIIME v1.9.1 and the Greengenes database^70,71^. OTUs formed from single reads were discarded. Samples with <15,000 sequences per sample were removed from the analysis.

### Statistical analyses

All analyses were performed in R v3.3.3^72^. Within-sample (alpha) diversity was calculated using the Shannon diversity index^73^. Between-sample (beta) diversity of *matK* and *rbcL* data was calculated using unweighted UniFrac distances after rarefaction to 5000 reads per sample^34^. Between-sample diversity of 16S rRNA data was also calculated using unweighted UniFrac, but after rarefaction to 15,000 reads per sample^34^. We opted to use unweighted distance values because they permit the examination of rare taxa that might be related to the phenotype examined; however, weighted UniFrac as well as weighted and unweighted Bray–Curtis dissimilarity values gave comparable results (*matk*: all Mantel tests *r* > 0.34, *p* < 10^−6^; *rbcl*: all Mantel tests *r* > 0.68, *p* < 10^−6^; 16S: all Mantel tests *r* > 0.77, *p* < 10^−6^). Unweighted UniFrac distances were also used for principal coordinates analysis^74^, *t*-distributed stochastic neighbour embedding^75^ and permutational analysis of variance (http://CRAN.R-project.org/package=vegan)^76^. Analyses of 16S rRNA data revealed that TL2 bonobo samples formed three distinct clusters (Supplementary Fig. 8c), corresponding to three different sampling locations west (TL2-W) and east (TL2-E and TL2-NE) of the Lomami River (Fig. 5b). To control for site-specific differences in faecal plant composition, we measured depletion of *matK* and *rbcL* OTUs between samples from TL2-E, TL2-NE and TL2-W and three non-endemic LK, KR and IK field sites using Wilcoxon rank sum tests. The *p*-values from the nine pairwise comparisons were combined using Fisher’s method with the test statistic and degrees of freedom divided by 3 to control for correlation between tests. Changes in bacterial OTU proportions between TL2 *Laverania-*positive and -negative samples were measured using Wilcoxon rank sum tests.

### Nomenclatural acts

This published work and the nomenclatural acts it contains have been registered in ZooBank, the proposed online registration system for the International Code of Zoological Nomenclature (ICZN). The ZooBank LSIDs (Life Science Identifiers) can be resolved and the associated information viewed through any standard web browser by appending the LSID to the prefix 'http://zoobank.org/'. The LSID for this publication is: urn:lsid:zoobank.org:act:BF143A7B-DA74-469D-B44C-40B5EF82B2DB.

### Code availability

Analysis code is archived on Zenodo (https://zenodo.org/record/886174) at DOI: (http://doi.org/10.5281/zenodo.886174).

### Data availability

Newly derived *Laverania* and non-*Laverania* parasite sequences as well as bonobo mtDNA haplotypes have been deposited in GenBank under accession numbers KY790455-KY790593 (also see Supplementary Data 1 and 2). High-throughput plant and microbiome sequences are archived in the NCBI Sequence Read Archive (SRA) under BioProject PRJNA389566.

## Electronic supplementary material

Supplementary Information

Description of Additional Supplementary Files

Supplementary Dataset 1

Supplementary Dataset 2
