# Supplementary material for: Wild bonobos host geographically restricted malaria parasites including a putative new Laverania species
Source: Nat Commun. 2017 Nov 21;8:1635. doi: 10.1038/s41467-017-01798-5 (PMC5696340; doi:10.1038/s41467-017-01798-5)
Supplement: Supplementary file 1 — Supplementary Information [file 41467_2017_1798_MOESM1_ESM.pdf]

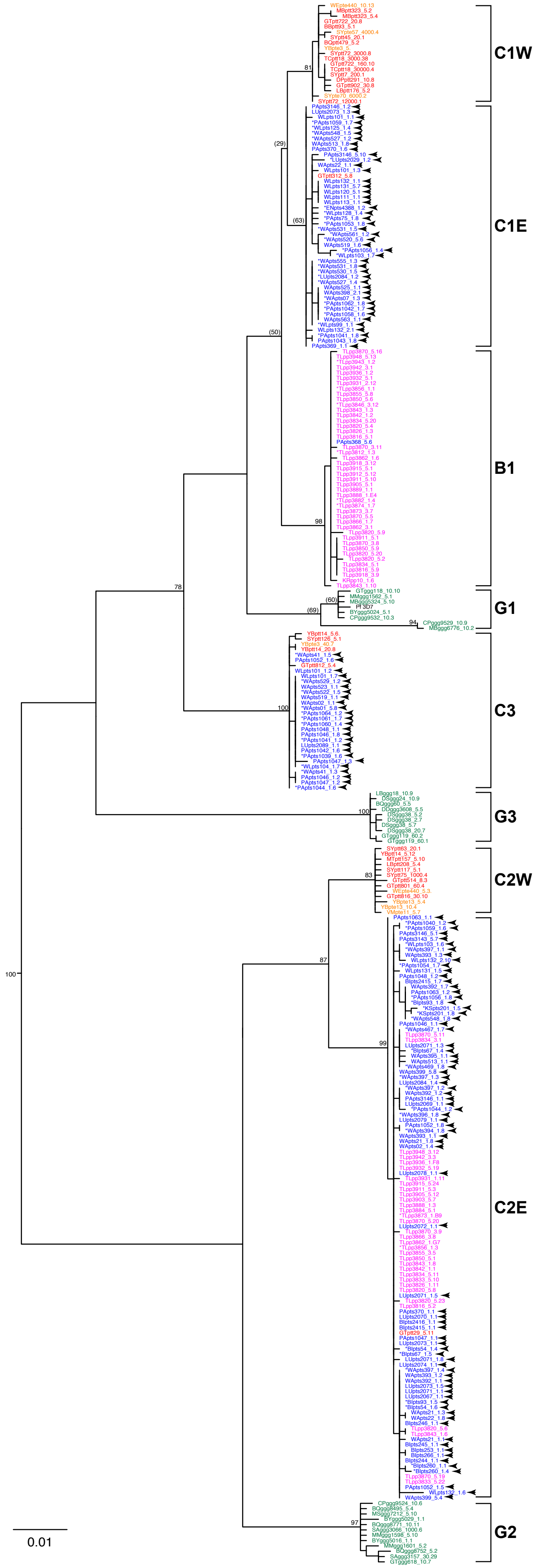
C1W
C1E
B1
G1
C3
G3
C2W
C2E
G2
81
(29)
(63)
(50)
98
78
(60)
(69)
94
100
100
83
87
99
97
100
0.01

Supplementary Figure 1 Evolutionary relationships of *Laverania* mitochondrial sequences. A maximum likelihood tree of cytochrome B (*cytB*) sequences (956 bp) from chimpanzee, gorilla, and bonobo *Laverania* parasites is shown. Sequences are colour-coded, with capital letters indicating their field site of origin (see Fig. 1 and ref. 1 for their location) and lower case letters denoting their host species and subspecies origin (pp: *Pan paniscus*, magenta; ptt: *P. t. troglodytes,* red; pte: *P. t. ellioti,* orange; pts: *P. t. schweinfurthii,* blue; ggg: *G. g. gorilla,* green). Asterisks indicate sequences derived by intensified PCR, all of which were confirmed to be single template derived (e.g., PApts1059_1.7 represents an intensified PCR derived sequence amplified from the undiluted PApts1059 faecal DNA and identified at position 7 in a plate of multiple PCR replicates). The remaining faecal derived ape parasite sequences were generated by single genome amplification (SGA) (e.g., GTptt312_5.8 represents an SGA derived sequence amplified from a 1:5 dilution of GTptt312 faecal DNA and identified at position 8 in a plate of multiple PCR reactions). Brackets indicate six previously defined *Laverania* species, with C1, C2 and C3 denoting the chimpanzee parasites *Plasmodium reichenowi*, *P. gaboni*, and *P. billcollinsi*, and G1, G2 and G3 the gorilla parasites *P. praefalciparum*, *P. adleri*, and *P. blacklocki*, respectively. Sequences from *P. reichenowi* and *P. gaboni* segregate into geographic ("western", W; "eastern", E) subclades. Newly derived bonobo parasite sequences (n=77) fall into two *Laverania* lineages, including *P. gaboni* from eastern chimpanzees (C2E) and a new distinct clade (B1) that appears to be host-specific (see text for a description of the single B1 *cytB* sequence from an eastern chimpanzee sample PApts368). Identical sequences from different samples are shown (identical sequences from the same sample are excluded). The human *P. falciparum* reference sequence 3D7 is shown in black. Arrows indicate parasite sequences newly generated (n=147) from faecal DNA of eastern chimpanzees collected at field sites close to the bonobo range (Fig. 1), all of which cluster within *P. reichenowi* (C1E), *P. gaboni* (C2E) or *P. billcollinsi* (C3). The tree was constructed using PhyML[2] with HKY+I+G as the evolutionary model. Bootstrap support values ≥ 70% are shown for major nodes only (the scale bar represents 0.01 substitutions per site).

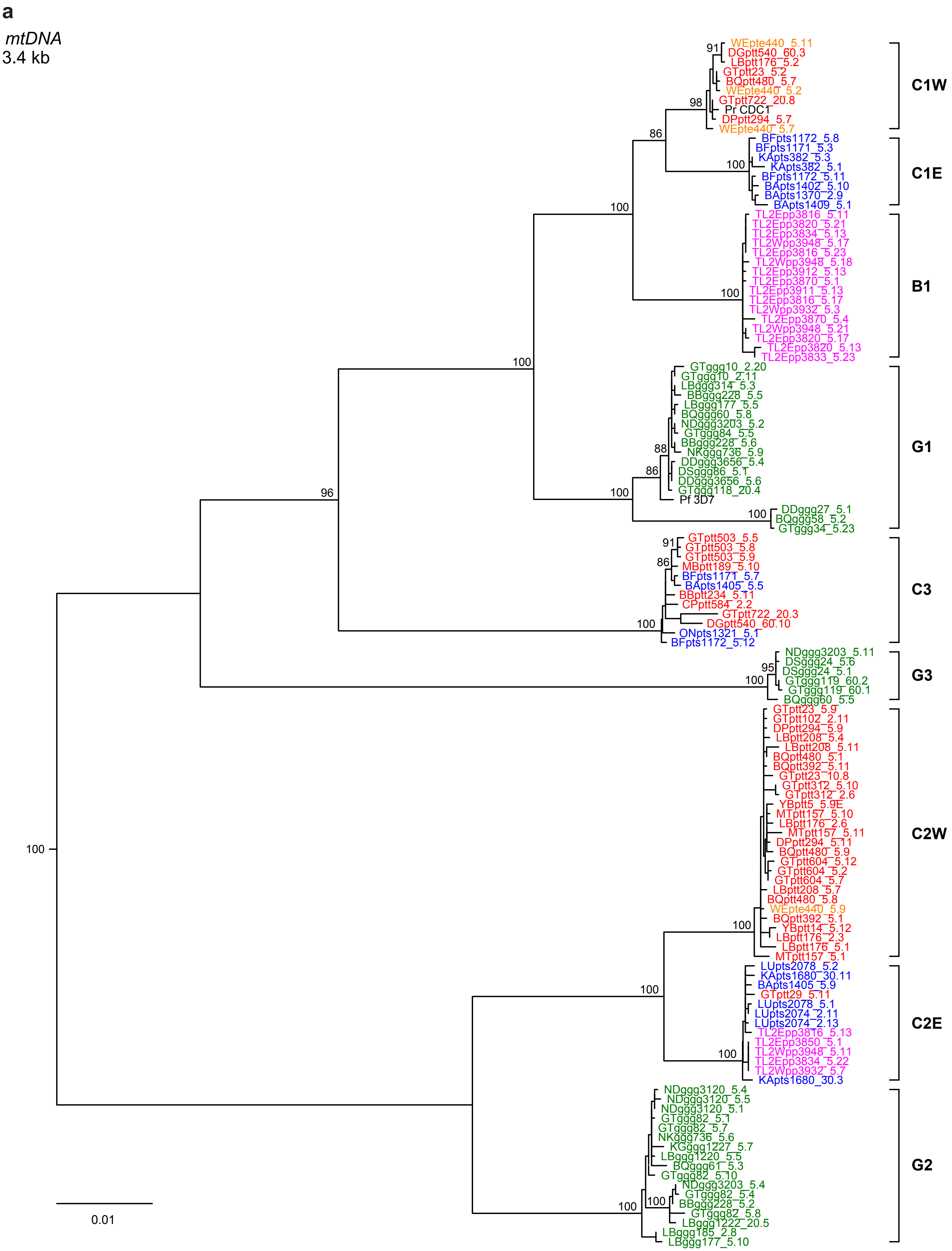
a
mtDNA
3.4 kb
WEpte440_5.11
DGptt540_60.3
LBptt176_5.2
GTptt23_5.2
BQptt480_5.7
WEpte440_5.2
GTptt722_20.8
Pr CDC1
DPptt294_5.7
WEpte440_5.7
BFpts1172_5.8
BFpts1171_5.3
KApts382_5.3
KApts382_5.1
BFpts1172_5.11
BApts1402_5.10
BApts1370_2.9
BApts1409_5.1
TL2Epp3816_5.11
TL2Epp3820_5.21
TL2Epp3834_5.13
TL2Wpp3948_5.17
TL2Epp3816_5.23
TL2Wpp3948_5.18
TL2Epp3912_5.13
TL2Epp3870_5.1
TL2Epp3911_5.13
TL2Epp3816_5.17
TL2Wpp3932_5.3
TL2Epp3870_5.4
TL2Wpp3948_5.21
TL2Epp3820_5.17
TL2Epp3820_5.13
TL2Epp3833_5.23
GTggg10_2.20
GTggg10_2.11
LBggg314_5.3
BBggg228_5.5
LBggg177_5.5
BQggg60_5.8
NDggg3203_5.2
GTggg84_5.5
BBggg228_5.6
NKggg736_5.9
DDggg3656_5.4
DSggg86_5.1
DDggg3656_5.6
GTggg118_20.4
Pf 3D7
DDggg27_5.1
BQggg58_5.2
GTggg34_5.23
GTptt503_5.5
GTptt503_5.8
GTptt503_5.9
MBptt189_5.10
BFpts1171_5.7
BApts1405_5.5
BBptt234_5.11
CPptt584_2.2
GTptt722_20.3
DGptt540_60.10
ONpts1321_5.1
BFpts1172_5.12
NDggg3203_5.11
DSggg24_5.6
DSggg24_5.1
GTggg119_60.2
GTggg119_60.1
BQggg60_5.5
GTptt23_5.9
GTptt102_2.11
DPptt294_5.9
LBptt208_5.4
LBptt208_5.11
BQptt480_5.1
BQptt392_5.11
GTptt23_10.8
GTptt312_5.10
GTptt312_2.6
YBptt5_5.9E
MTptt157_5.10
LBptt176_2.6
MTptt157_5.11
DPptt294_5.11
BQptt480_5.9
GTptt604_5.12
GTptt604_5.2
GTptt604_5.7
LBptt208_5.7
BQptt480_5.8
WEpte440_5.9
BQptt392_5.1
YBptt14_5.12
LBptt176_2.3
LBptt176_5.1
MTptt157_5.1
LUpts2078_5.2
KApts1680_30.11
BApts1405_5.9
GTptt29_5.11
LUpts2078_5.1
LUpts2074_2.11
LUpts2074_2.13
TL2Epp3816_5.13
TL2Epp3850_5.1
TL2Wpp3948_5.11
TL2Epp3834_5.22
TL2Wpp3932_5.7
KApts1680_30.3
NDggg3120_5.4
NDggg3120_5.5
NDggg3120_5.1
GTggg82_5.1
GTggg82_5.7
NKggg736_5.6
KGggg1227_5.7
LBggg1220_5.5
BQggg61_5.3
GTggg82_5.10
NDggg3203_5.4
GTggg82_5.4
BBggg228_5.2
GTggg82_5.8
LBggg1222_20.5
LBggg185_2.8
LBggg177_5.10
91
98
86
100
100
100
96
88
86
100
100
91
86
100
95
100
100
100
100
100
100
100
100
0.01
C1W
C1E
B1
G1
C3
G3
C2W
C2E
G2

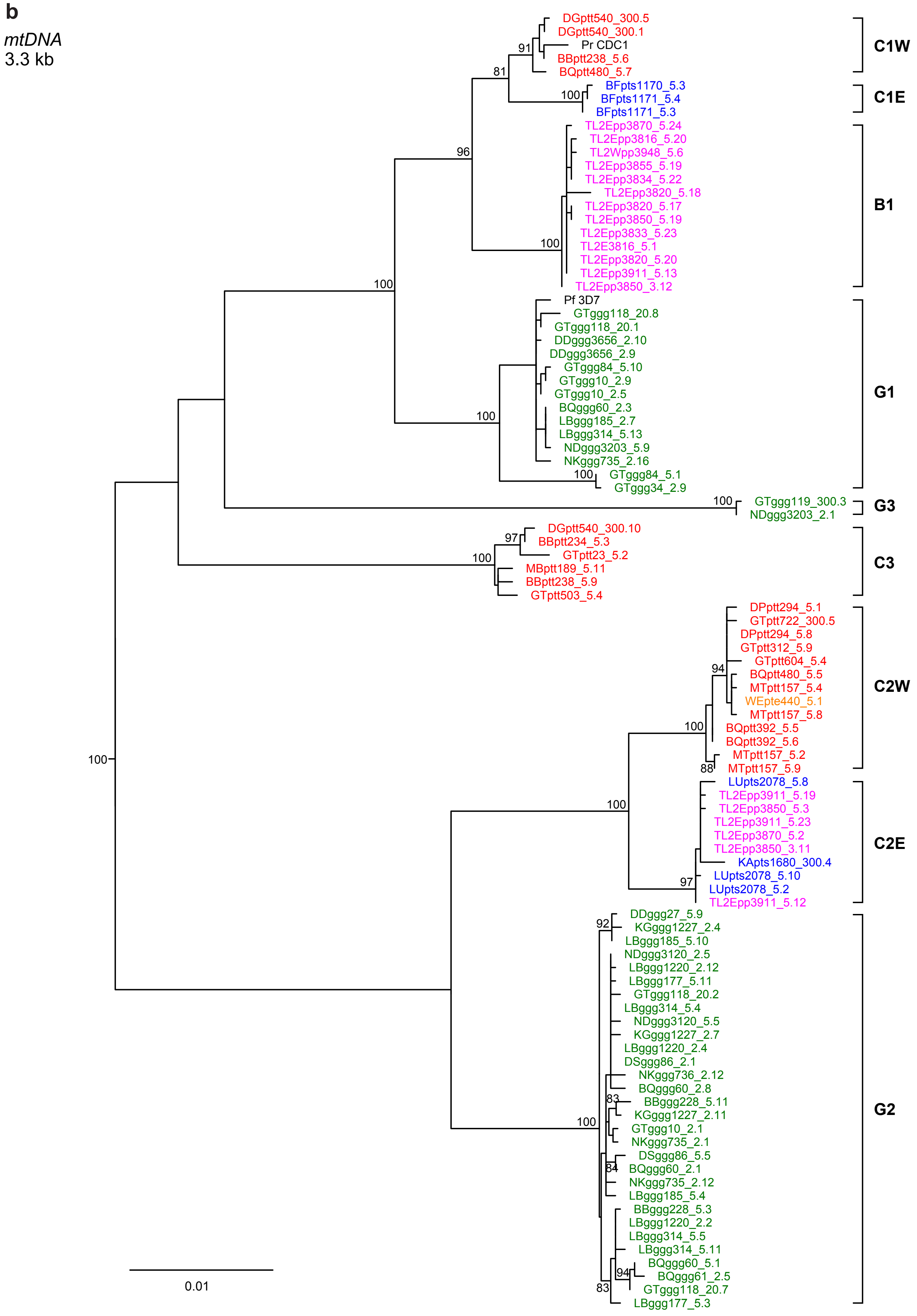
b
mtDNA
3.3 kb
DGptt540_300.5
DGptt540_300.1
Pr CDC1
BBptt238_5.6
BQptt480_5.7
BFpts1170_5.3
BFpts1171_5.4
BFpts1171_5.3
TL2Epp3870_5.24
TL2Epp3816_5.20
TL2Wpp3948_5.6
TL2Epp3855_5.19
TL2Epp3834_5.22
TL2Epp3820_5.18
TL2Epp3820_5.17
TL2Epp3850_5.19
TL2Epp3833_5.23
TL2E3816_5.1
TL2Epp3820_5.20
TL2Epp3911_5.13
TL2Epp3850_3.12
Pf 3D7
GTggg118_20.8
GTggg118_20.1
DDggg3656_2.10
DDggg3656_2.9
GTggg84_5.10
GTggg10_2.9
GTggg10_2.5
BQggg60_2.3
LBggg185_2.7
LBggg314_5.13
NDggg3203_5.9
NKggg735_2.16
GTggg84_5.1
GTggg34_2.9
GTggg119_300.3
NDggg3203_2.1
DGptt540_300.10
BBptt234_5.3
GTptt23_5.2
MBptt189_5.11
BBptt238_5.9
GTptt503_5.4
DPptt294_5.1
GTptt722_300.5
DPptt294_5.8
GTptt312_5.9
GTptt604_5.4
BQptt480_5.5
MTptt157_5.4
WEpte440_5.1
MTptt157_5.8
BQptt392_5.5
BQptt392_5.6
MTptt157_5.2
MTptt157_5.9
LUpts2078_5.8
TL2Epp3911_5.19
TL2Epp3850_5.3
TL2Epp3911_5.23
TL2Epp3870_5.2
TL2Epp3850_3.11
KApts1680_300.4
LUpts2078_5.10
LUpts2078_5.2
TL2Epp3911_5.12
DDggg27_5.9
KGggg1227_2.4
LBggg185_5.10
NDggg3120_2.5
LBggg1220_2.12
LBggg177_5.11
GTggg118_20.2
LBggg314_5.4
NDggg3120_5.5
KGggg1227_2.7
LBggg1220_2.4
DSggg86_2.1
NKggg736_2.12
BQggg60_2.8
BBggg228_5.11
KGggg1227_2.11
GTggg10_2.1
NKggg735_2.1
DSggg86_5.5
BQggg60_2.1
NKggg735_2.12
LBggg185_5.4
BBggg228_5.3
LBggg1220_2.2
LBggg314_5.5
LBggg314_5.11
BQggg60_5.1
BQggg61_2.5
GTggg118_20.7
LBggg177_5.3
91
81
100
96
100
100
100
100
100
97
100
94
100
88
100
97
92
83
100
84
94
83
100
C1W
C1E
B1
G1
G3
C3
C2W
C2E
G2
0.01

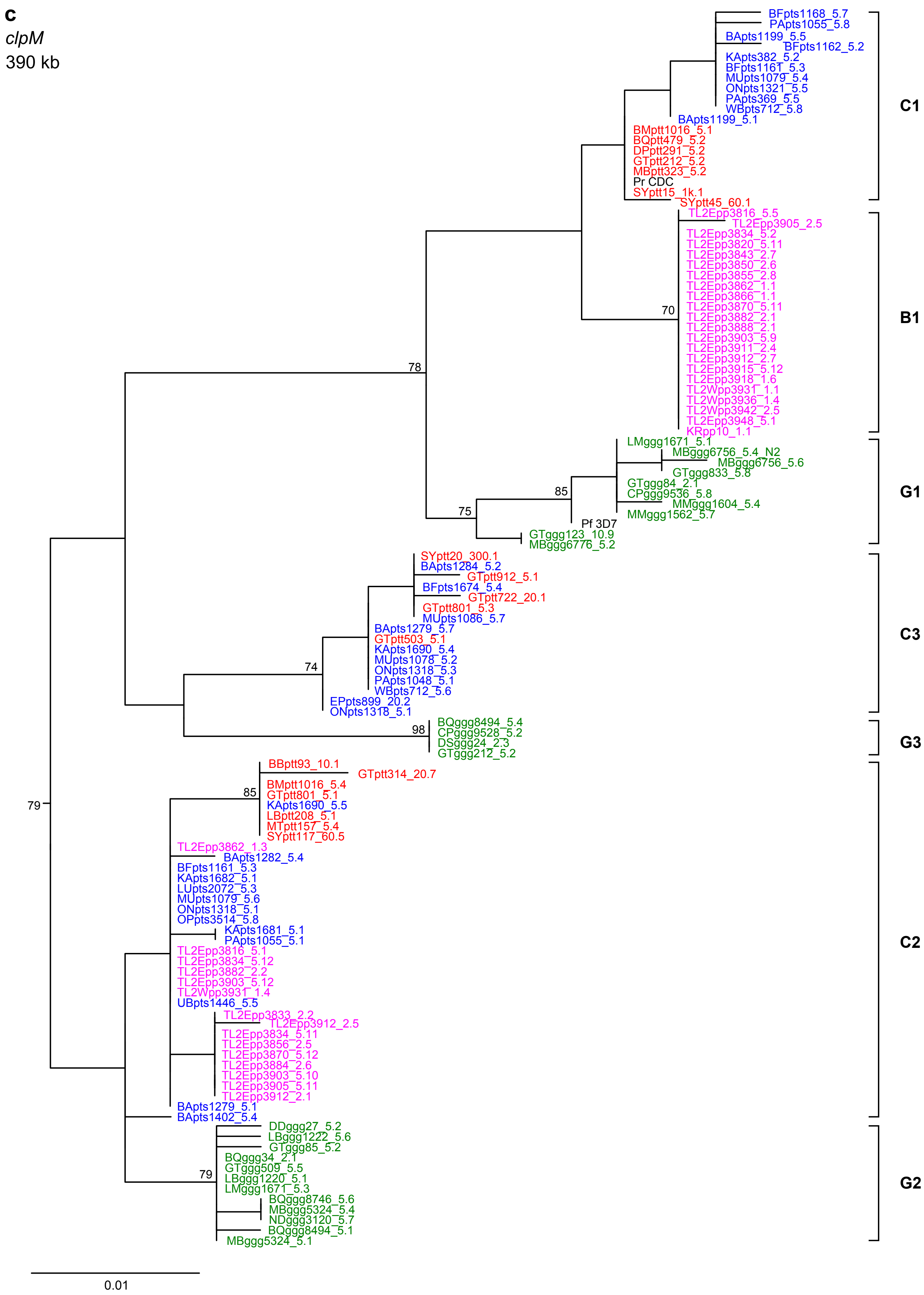
c
clpM
390 kb
BFpts1168_5.7
PApts1055_5.8
BApts1199_5.5
BFpts1162_5.2
KApts382_5.2
BFpts1161_5.3
MUpts1079_5.4
ONpts1321_5.5
PApts369_5.5
WBpts712_5.8
BApts1199_5.1
BMptt1016_5.1
BQptt479_5.2
DPptt291_5.2
GTptt212_5.2
MBptt323_5.2
Pr CDC
SYptt15_1k.1
SYptt45_60.1
TL2Epp3816_5.5
TL2Epp3905_2.5
TL2Epp3834_5.2
TL2Epp3820_5.11
TL2Epp3843_2.7
TL2Epp3850_2.6
TL2Epp3855_2.8
TL2Epp3862_1.1
TL2Epp3866_1.1
TL2Epp3870_5.11
TL2Epp3882_2.1
TL2Epp3888_2.1
TL2Epp3903_5.9
TL2Epp3911_2.4
TL2Epp3912_2.7
TL2Epp3915_5.12
TL2Epp3918_1.6
TL2Wpp3931_1.1
TL2Wpp3936_1.4
TL2Wpp3942_2.5
TL2Epp3948_5.1
KRpp10_1.1
LMggg1671_5.1
MBggg6756_5.4_N2
MBggg6756_5.6
GTggg833_5.8
GTggg84_2.1
CPggg9536_5.8
MMggg1604_5.4
MMggg1562_5.7
Pf 3D7
GTggg123_10.9
MBggg6776_5.2
SYptt20_300.1
BApts1284_5.2
GTptt912_5.1
BFpts1674_5.4
GTptt722_20.1
GTptt801_5.3
MUpts1086_5.7
BApts1279_5.7
GTptt503_5.1
KApts1690_5.4
MUpts1078_5.2
ONpts1318_5.3
PApts1048_5.1
WBpts712_5.6
EPpts899_20.2
ONpts1318_5.1
BQggg8494_5.4
CPggg9528_5.2
DSggg24_2.3
GTggg212_5.2
BBptt93_10.1
GTptt314_20.7
BMptt1016_5.4
GTptt801_5.1
KApts1690_5.5
LBptt208_5.1
MTptt157_5.4
SYptt117_60.5
TL2Epp3862_1.3
BApts1282_5.4
BFpts1161_5.3
KApts1682_5.1
LUpts2072_5.3
MUpts1079_5.6
ONpts1318_5.1
OPpts3514_5.8
KApts1681_5.1
PApts1055_5.1
TL2Epp3816_5.1
TL2Epp3834_5.12
TL2Epp3882_2.2
TL2Epp3903_5.12
TL2Wpp3931_1.4
UBpts1446_5.5
TL2Epp3833_2.2
TL2Epp3912_2.5
TL2Epp3834_5.11
TL2Epp3856_2.5
TL2Epp3870_5.12
TL2Epp3884_2.6
TL2Epp3903_5.10
TL2Epp3905_5.11
TL2Epp3912_2.1
BApts1279_5.1
BApts1402_5.4
DDggg27_5.2
LBggg1222_5.6
GTggg85_5.2
BQggg34_2.1
GTggg509_5.5
LBggg1220_5.1
LMggg1671_5.3
BQggg8746_5.6
MBggg5324_5.4
NDggg3120_5.7
BQggg8494_5.1
MBggg5324_5.1
78
70
85
75
74
98
85
79
79
C1
B1
G1
C3
G3
C2
G2
0.01

d

eba175

394 kb

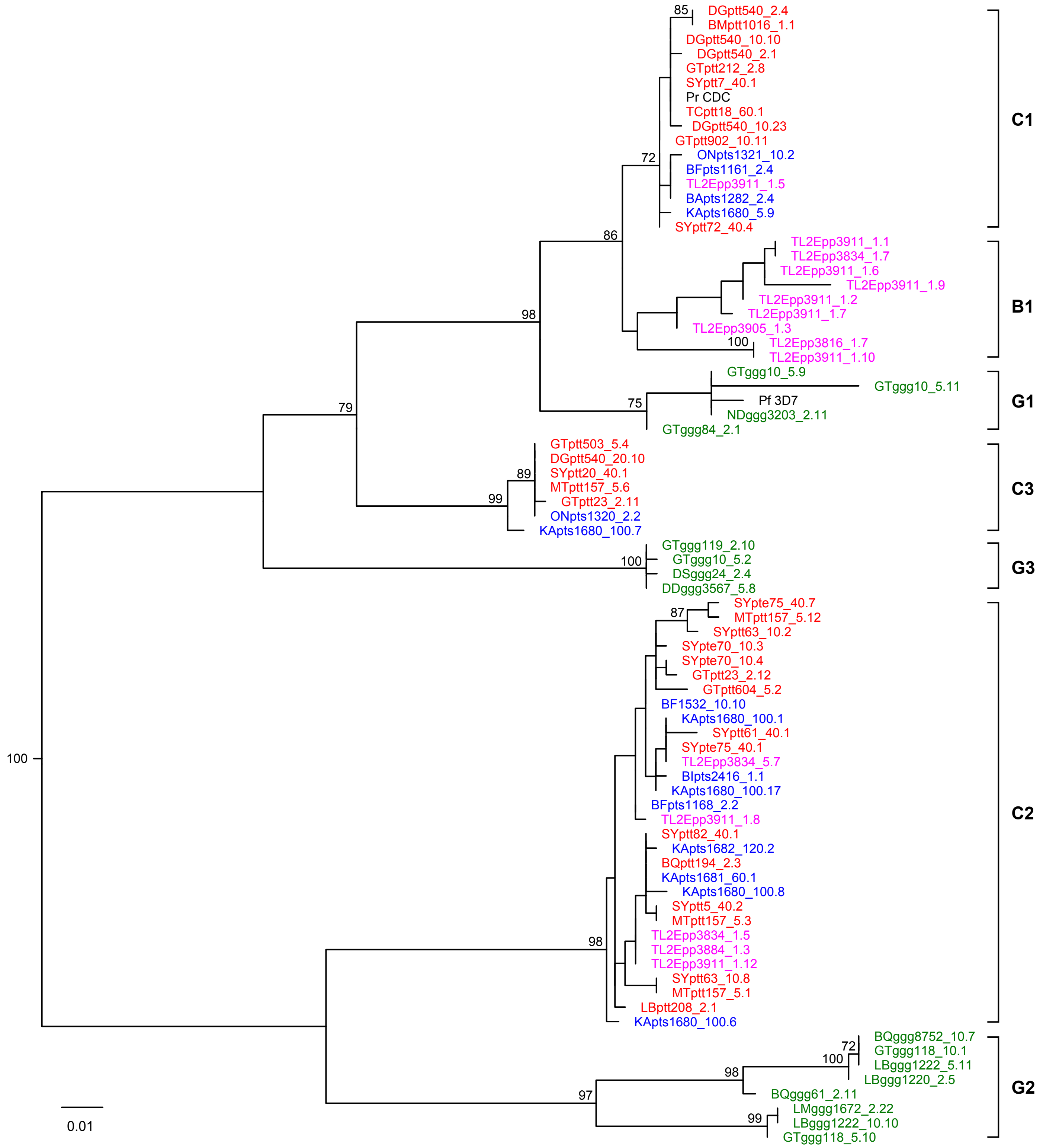

Supplementary Figure 2 Wild-living bonobos harbour two *Laverania* species. Maximum likelihood trees depicting the phylogenetic relationships of (**a, b**) mitochondrial (3.4 and 3.3 kb half genomes), (**c**) apicoplast (*clpM* gene; 390 bp), and (**d**) nuclear (erythrocyte binding antigen 175; 394 bp) gene sequences of *Laverania* parasites are shown. Sequences are colour-coded to indicate the host species (pp: *Pan paniscus*, magenta; ptt: *P. t. troglodytes,* red; pte: *P. t. ellioti,* orange; pts: *P. t. schweinfurthii,* blue; ggg: *G. g. gorilla,* green) and are otherwise labeled as in Supplementary Fig. 1. Identical sequences from different samples are shown (identical sequences from the same sample are excluded). Brackets identify six previously defined *Laverania* species, with C1, C2 and C3 denoting the chimpanzee parasites *Plasmodium reichenowi*, *P. gaboni*, and *P. billcollinsi*, and G1, G2 and G3 the gorilla parasites *P. praefalciparum*, *P. adleri*, and *P. blacklocki*, respectively (mitochondrial sequences from *P. reichenowi* and *P. gaboni* segregate into "western" (W) and "eastern" (E) subclades). In all genomic regions, the newly derived bonobo parasite sequences either cluster with *P. gaboni* from eastern chimpanzees or form a new clade (B1) that appears to be host-specific. Reference sequences for *P. falciparum* (Pf 3D7) and *P. reichenowi* (Pr CDC1) are shown in black. The trees were constructed using PhyML[2] with TIM2+I+G (**a**), TIM3+I+G (**b**) HKY+G (**c**) and TPM3uf+G (**d**) as evolutionary models. Bootstrap values ≥ 70% are shown for major nodes only (the scale bar represents 0.01 substitutions per site).

a

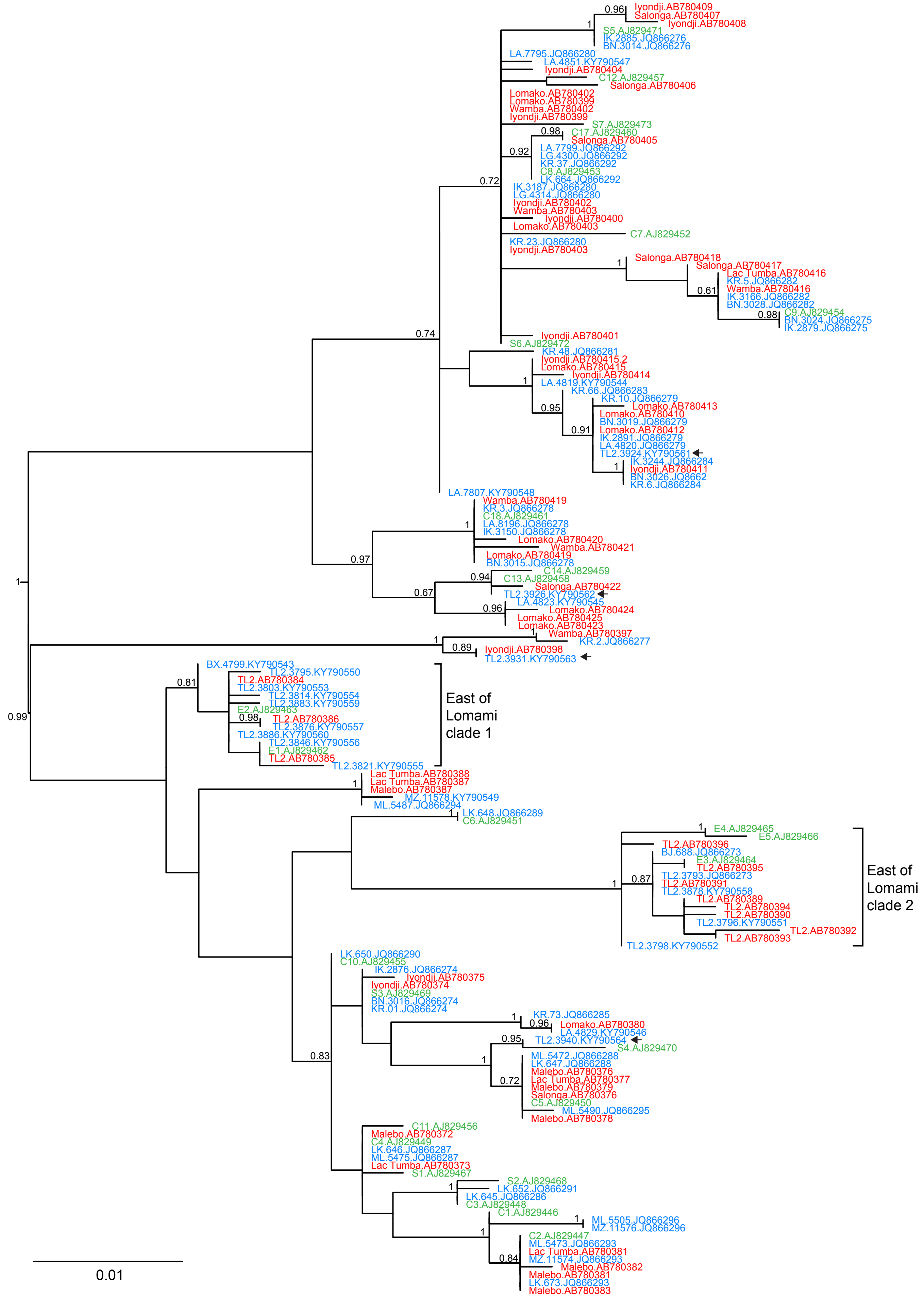
Iyondji.AB780409
Salonga.AB780407
Iyondji.AB780408
S5.AJ829471
IK.2885.JQ866276
BN.3014.JQ866276
LA.7795.JQ866280
LA.4851.KY790547
Iyondji.AB780404
C12.AJ829457
Salonga.AB780406
Lomako.AB780402
Lomako.AB780399
Wamba.AB780402
Iyondji.AB780399
S7.AJ829473
C17.AJ829460
Salonga.AB780405
LA.7799.JQ866292
LG.4300.JQ866292
KR.37.JQ866292
C8.AJ829453
LK.664.JQ866292
IK.3187.JQ866280
LG.4314.JQ866280
Iyondji.AB780402
Wamba.AB780403
Iyondji.AB780400
Lomako.AB780403
C7.AJ829452
KR.23.JQ866280
Iyondji.AB780403
Salonga.AB780418
Salonga.AB780417
Lac Tumba.AB780416
KR.5.JQ866282
Wamba.AB780416
IK.3166.JQ866282
BN.3028.JQ866282
C9.AJ829454
BN.3024.JQ866275
IK.2879.JQ866275
Iyondji.AB780401
S6.AJ829472
KR.48.JQ866281
Iyondji.AB780415.2
Lomako.AB780415
Iyondji.AB780414
LA.4819.KY790544
KR.66.JQ866283
KR.10.JQ866279
Lomako.AB780413
Lomako.AB780410
BN.3019.JQ866279
Lomako.AB780412
IK.2891.JQ866279
LA.4820.JQ866279
TL2.3924.KY790561
IK.3244.JQ866284
Iyondji.AB780411
BN.3026.JQ8662
KR.6.JQ866284
LA.7807.KY790548
Wamba.AB780419
KR.3.JQ866278
C18.AJ829461
LA.8196.JQ866278
IK.3150.JQ866278
Lomako.AB780420
Wamba.AB780421
Lomako.AB780419
BN.3015.JQ866278
C14.AJ829459
C13.AJ829458
Salonga.AB780422
TL2.3926.KY790562
LA.4823.KY790545
Lomako.AB780424
Lomako.AB780425
Lomako.AB780423
Wamba.AB780397
KR.2.JQ866277
Iyondji.AB780398
TL2.3931.KY790563
BX.4799.KY790543
TL2.3795.KY790550
TL2.AB780384
TL2.3803.KY790553
TL2.3814.KY790554
TL2.3883.KY790559
E2.AJ829463
TL2.AB780386
TL2.3876.KY790557
TL2.3886.KY790560
TL2.3846.KY790556
E1.AJ829462
TL2.AB780385
TL2.3821.KY790555
East of Lomami clade 1
Lac Tumba.AB780388
Lac Tumba.AB780387
Malebo.AB780387
MZ.11578.KY790549
ML.5487.JQ866294
LK.648.JQ866289
C6.AJ829451
E4.AJ829465
E5.AJ829466
TL2.AB780396
BJ.688.JQ866273
E3.AJ829464
TL2.AB780395
TL2.3793.JQ866273
TL2.AB780391
TL2.3878.KY790558
TL2.AB780389
TL2.AB780394
TL2.AB780390
TL2.3796.KY790551
TL2.AB780392
TL2.AB780393
TL2.3798.KY790552
East of Lomami clade 2
LK.650.JQ866290
C10.AJ829455
IK.2876.JQ866274
Iyondji.AB780375
Iyondji.AB780374
S3.AJ829469
BN.3016.JQ866274
KR.01.JQ866274
KR.73.JQ866285
Lomako.AB780380
LA.4829.KY790546
TL2.3940.KY790564
S4.AJ829470
ML.5472.JQ866288
LK.647.JQ866288
Malebo.AB780376
Lac Tumba.AB780377
Malebo.AB780379
Salonga.AB780376
C5.AJ829450
ML.5490.JQ866295
Malebo.AB780378
C11.AJ829456
Malebo.AB780372
C4.AJ829449
LK.646.JQ866287
ML.5475.JQ866287
Lac Tumba.AB780373
S1.AJ829467
S2.AJ829468
LK.652.JQ866291
LK.645.JQ866286
C3.AJ829448
C1.AJ829446
ML.5505.JQ866296
MZ.11576.JQ866296
C2.AJ829447
ML.5473.JQ866293
Lac Tumba.AB780381
MZ.11574.JQ866293
Malebo.AB780382
Malebo.AB780381
LK.673.JQ866293
Malebo.AB780383
0.01

b

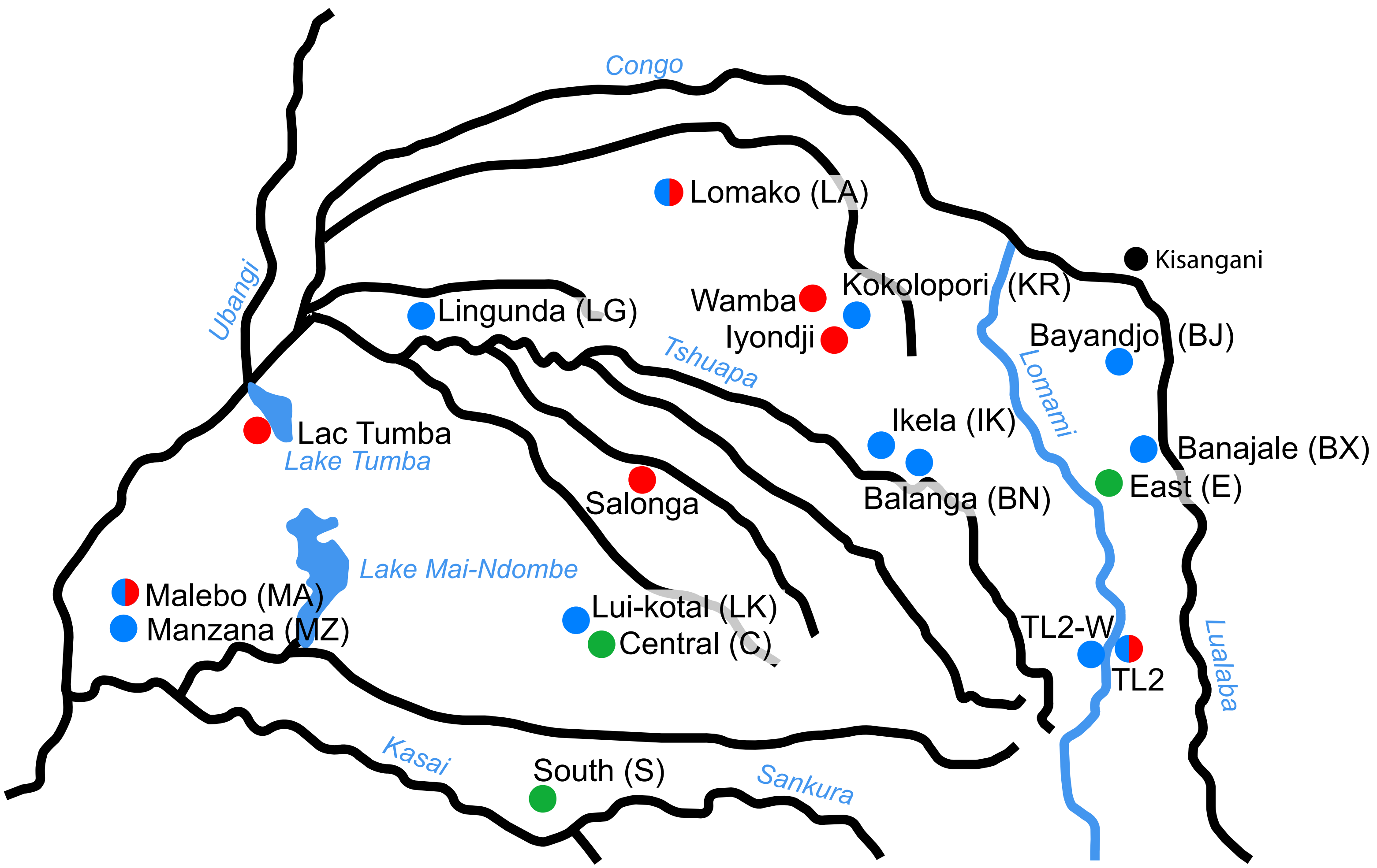

Congo
Ubangi
Lomako (LA)
Kisangani
Wamba
Kokolopori (KR)
Lingunda (LG)
Iyondji
Bayandjo (BJ)
Tshuapa
Lomami
Lac Tumba
Lake Tumba
Ikela (IK)
Banajale (BX)
East (E)
Salonga
Balanga (BN)
Lake Mai-Ndombe
Malebo (MA)
Manzana (MZ)
Lui-kotal (LK)
Central (C)
TL2-W
TL2
Lualaba
Kasai
South (S)
Sankura

Supplementary Figure 3 The Lomami River represents a barrier to bonobo gene flow. (**a**) A maximum likelihood tree of previously reported[3-5] and newly generated (n=165) bonobo mitochondrial haplotypes with known sampling location is shown. Sequences are labeled by field site and GenBank accession code, with colours indicating results from three different groups: Sequences from Eriksson and colleagues are shown in green[3]; sequences from Kawamoto and colleagues are shown in red[4]; and sequencing from Li and colleagues[5] and the current study are shown in blue. Identical sequences from different collection sites are shown (identical sequences from the same location are excluded). All haplotypes from bonobos sampled east of the Lomami River (brackets) fall into two separate clades, confirming limited gene flow across this riverine barrier. The four TL2 haplotypes that do not fall within these two clades were all sampled west of the Lomami River and are indicated by an arrow. The tree was constructed using PhyML[2] with HKY+G as the evolutionary model. Bayesian posterior probability values ≥ 0.6 are shown (the scale bar represents 0.01 substitutions per site). (**b**) Geographic locations of faecal collection sites from which the bonobo mitochondrial haplotypes shown in (**a**) were derived. The full names of the field sites are listed, with haplotype abbreviations shown in parentheses. Bonobo sampling sites are colour coded to match the haplotypes shown in (**a**), with sites/haplotypes described by Eriksson and colleagues shown in green[3]; by Kawamoto and colleagues shown in red[4]; and by Li and colleagues and the current study shown in blue[5]. Field sites where bonobos were independently sampled by two groups of investigators (Lomako, Malebo, TL2) are also denoted. The Lomami River is highlighted in blue; other rivers are shown in black.

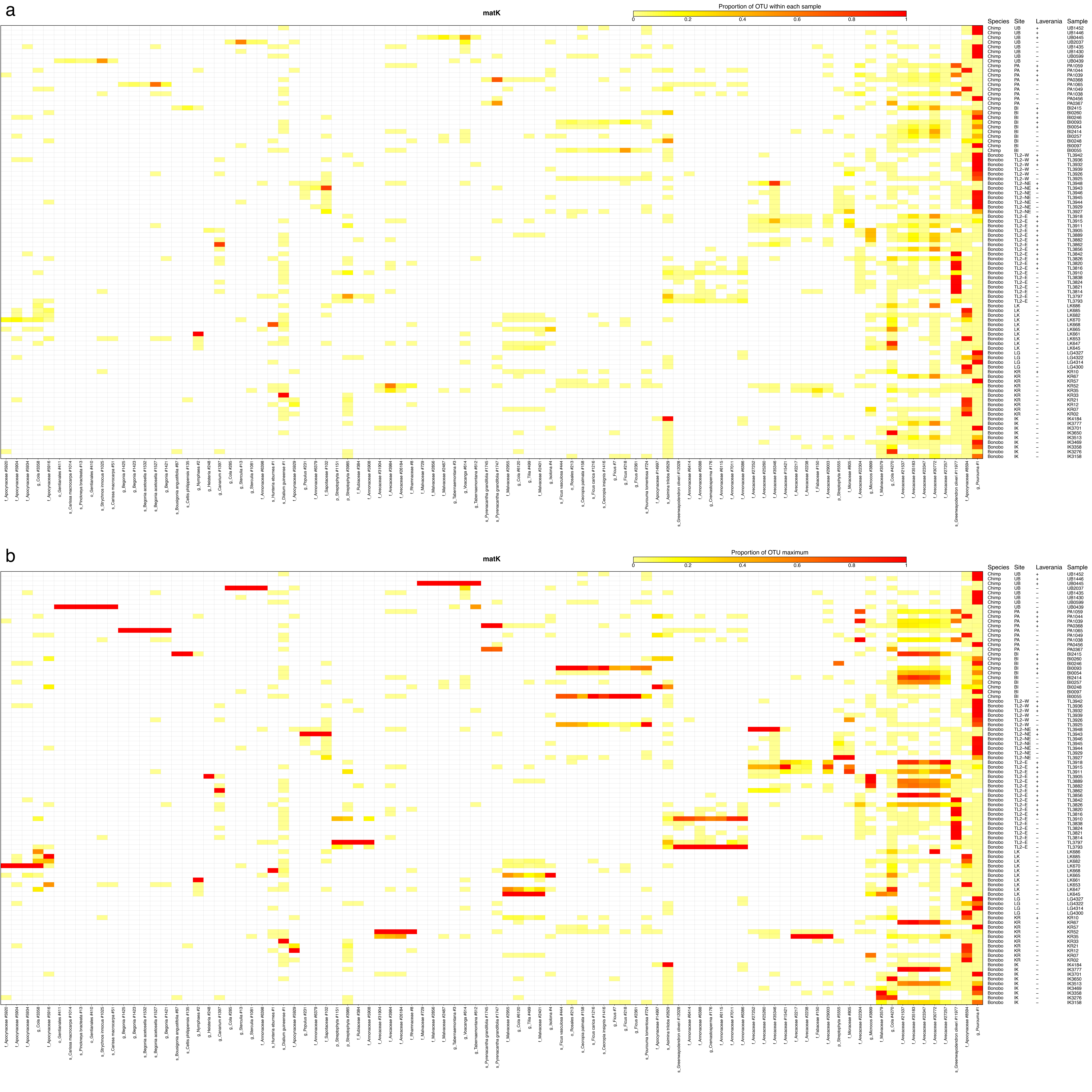

a
b
matK
Proportion of OTU within each sample
Proportion of OTU maximum
Species
Site
Laverania
Sample

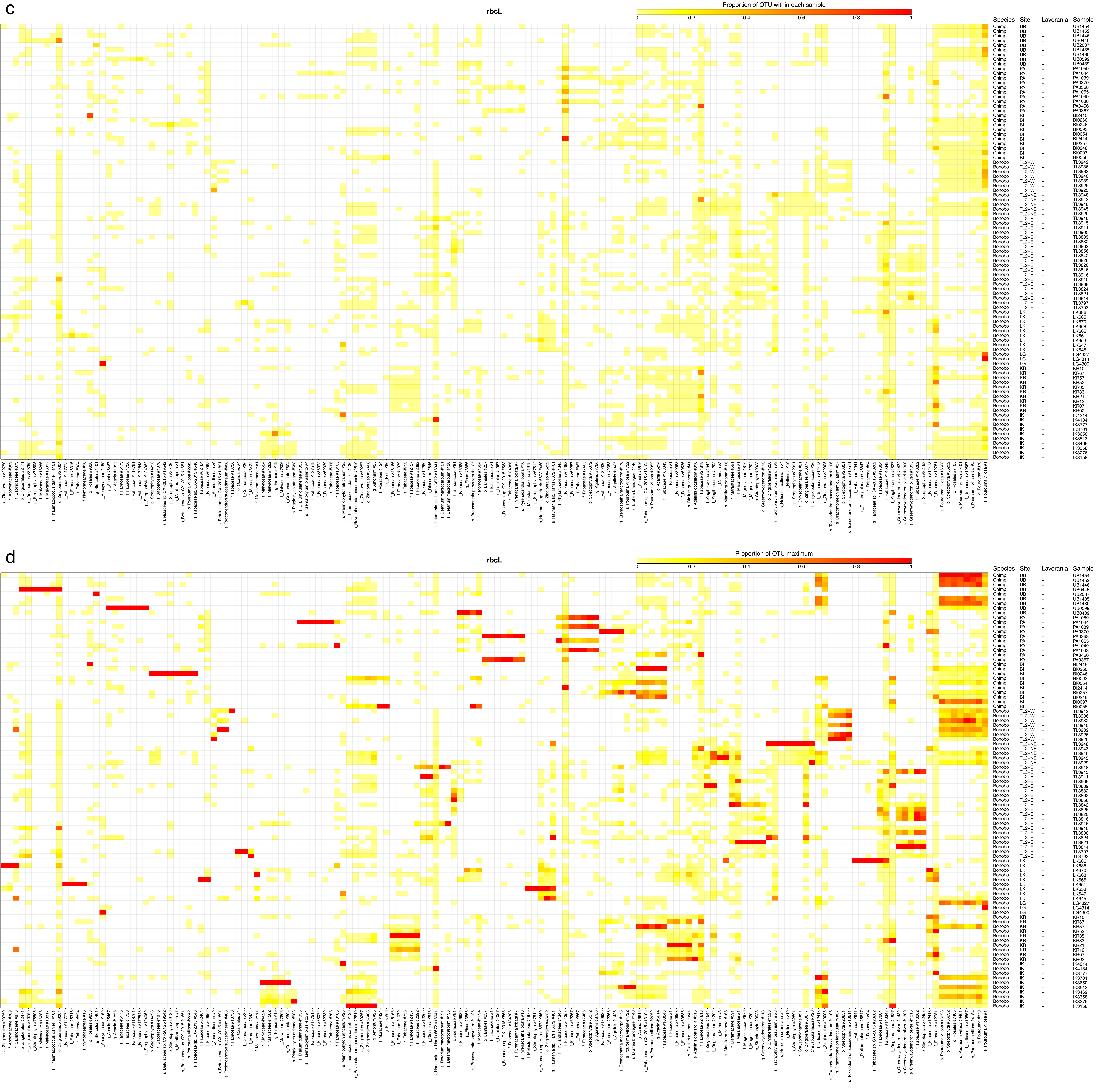
c
rbcL
Proportion of OTU within each sample
0
0.2
0.4
0.6
0.8
1
Species
Site
Laverania
Sample
d
rbcL
Proportion of OTU maximum
0
0.2
0.4
0.6
0.8
1
Species
Site
Laverania
Sample

Supplementary Figure 4 Relative abundance of major plant phyla in *Laverania* positive and negative ape faecal samples. (**a**) Heatmap of operational taxonomic units (OTUs) as determined by *matK* gene sequencing. Each grid entry represents the relative abundance of an OTU (column) within each sample (row). Only OTUs with an abundance of greater than 2% in at least one sample are shown. OTUs are ordered by co-occurrence to emphasise patterns in the data. Samples are labeled by ape species (chimpanzee and bonobo), field site (see Fig. 5b for the location of TL2-W, TL2-E and TL2-NE samples), as well as with a + and – prefix to indicate *Laverania* positive and negative status, respectively (see Supplementary Table 6 for a description of all samples). OTUs are labeled according to their most specific assigned taxonomic rank, with letters indicating order (o), family (f), genus (g), and species (s), followed by an arbitrary ID number. Plant taxa that could not be classified are labeled "Unknown". **(b)** Heatmap of OTUs as in (**a**) but with OTU abundance measured relative to the maximum proportion within that OTU (red cells indicate samples with the highest proportional abundance of the corresponding OTU). (**c**) Heatmap of OTUs as in (**a**) but for *rbcL* gene sequences. (**d**) Heatmap of OTUs as in (**b**) but for *rbcL* gene sequences.

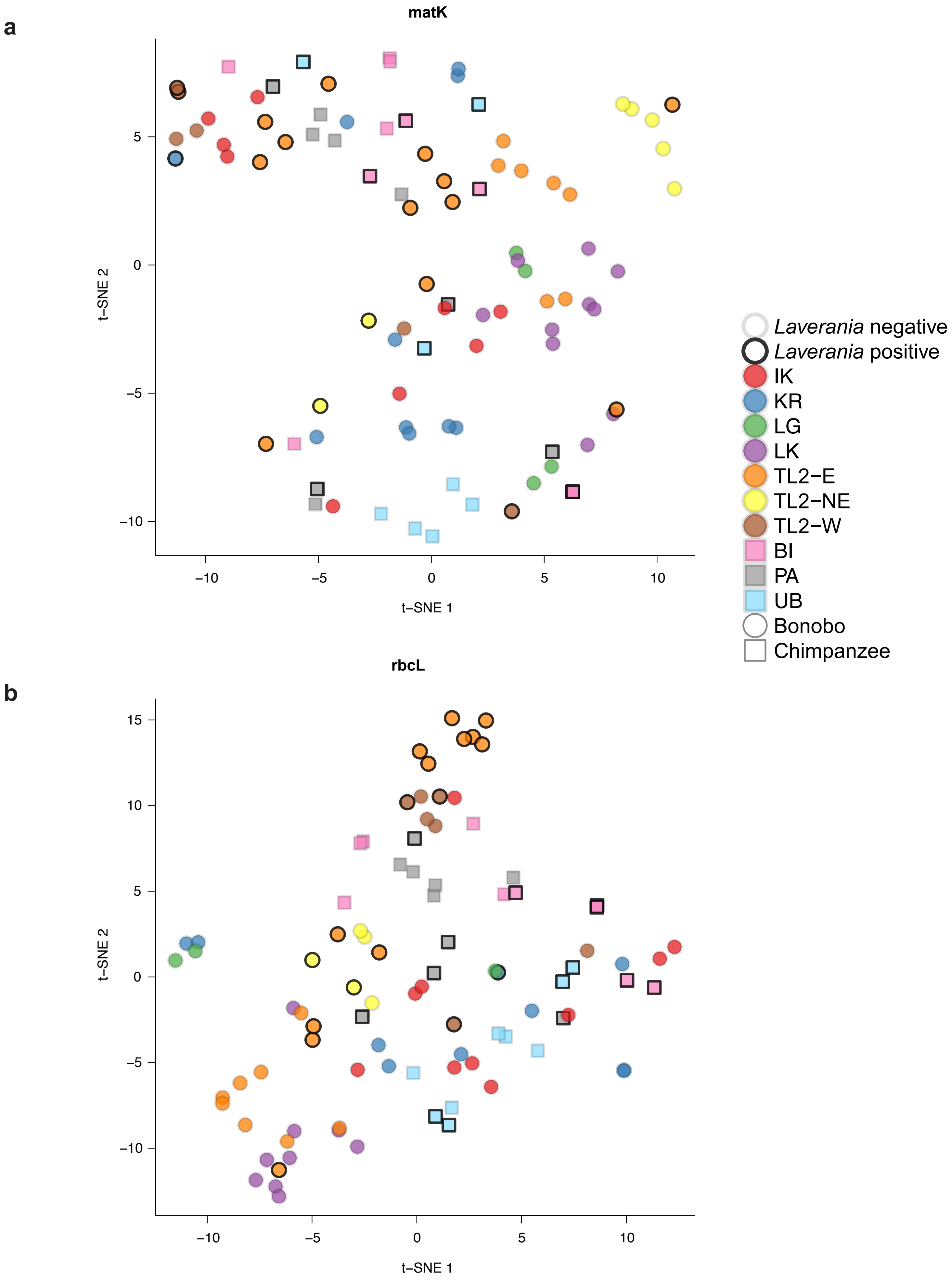
a
matK
b
rbcL
t−SNE 1
t−SNE 2
Laverania negative
Laverania positive
IK
KR
LG
LK
TL2−E
TL2−NE
TL2−W
BI
PA
UB
Bonobo
Chimpanzee

Supplementary Figure 5 Plant composition in *Laverania* positive and negative bonobo and chimpanzee faecal samples across study sites. A two-dimensional representation of unweighted Unifrac distances generated using t-distributed stochastic neighbor embedding (t-SNE) is shown for (**a**) chloroplast *matK* and (**b**) *rbcL* sequences, comparing faecal plant composition of bonobos (circles) and chimpanzees (squares) from various study sites (indicated by colour). *Laverania* positive faecal samples are highlighted by a dark outline. Note that during dimensional reduction, t-SNE attempts to preserve local differences so that similar samples cluster together, but the distance between samples with large differences is not necessarily preserved.

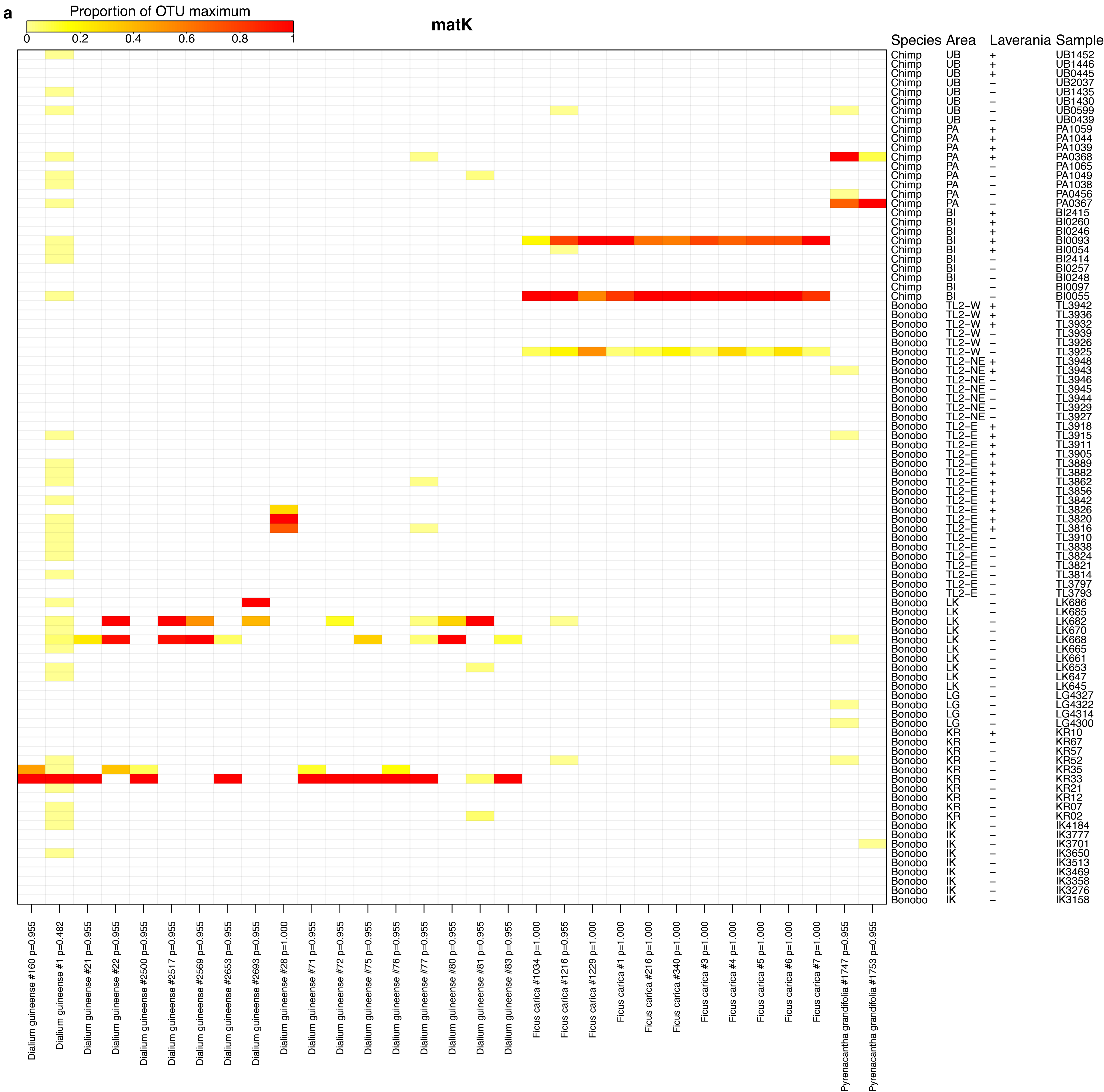
a
Proportion of OTU maximum
0 0.2 0.4 0.6 0.8 1
matK
Species Area Laverania Sample
Chimp UB + UB1452
Chimp UB + UB1446
Chimp UB + UB0445
Chimp UB – UB2037
Chimp UB – UB1435
Chimp UB – UB1430
Chimp UB – UB0599
Chimp UB – UB0439
Chimp PA + PA1059
Chimp PA + PA1044
Chimp PA + PA1039
Chimp PA + PA0368
Chimp PA – PA1065
Chimp PA – PA1049
Chimp PA – PA1038
Chimp PA – PA0456
Chimp PA – PA0367
Chimp BI + BI2415
Chimp BI + BI0260
Chimp BI + BI0246
Chimp BI + BI0093
Chimp BI + BI0054
Chimp BI – BI2414
Chimp BI – BI0257
Chimp BI – BI0248
Chimp BI – BI0097
Chimp BI – BI0055
Bonobo TL2–W + TL3942
Bonobo TL2–W + TL3936
Bonobo TL2–W + TL3932
Bonobo TL2–W – TL3939
Bonobo TL2–W – TL3926
Bonobo TL2–W – TL3925
Bonobo TL2–NE + TL3948
Bonobo TL2–NE + TL3943
Bonobo TL2–NE – TL3946
Bonobo TL2–NE – TL3945
Bonobo TL2–NE – TL3944
Bonobo TL2–NE – TL3929
Bonobo TL2–NE – TL3927
Bonobo TL2–E + TL3918
Bonobo TL2–E + TL3915
Bonobo TL2–E + TL3911
Bonobo TL2–E + TL3905
Bonobo TL2–E + TL3889
Bonobo TL2–E + TL3882
Bonobo TL2–E + TL3862
Bonobo TL2–E + TL3856
Bonobo TL2–E + TL3842
Bonobo TL2–E + TL3826
Bonobo TL2–E + TL3820
Bonobo TL2–E + TL3816
Bonobo TL2–E – TL3910
Bonobo TL2–E – TL3838
Bonobo TL2–E – TL3824
Bonobo TL2–E – TL3821
Bonobo TL2–E – TL3814
Bonobo TL2–E – TL3797
Bonobo TL2–E – TL3793
Bonobo LK – LK686
Bonobo LK – LK685
Bonobo LK – LK682
Bonobo LK – LK670
Bonobo LK – LK668
Bonobo LK – LK665
Bonobo LK – LK661
Bonobo LK – LK653
Bonobo LK – LK647
Bonobo LK – LK645
Bonobo LG – LG4327
Bonobo LG – LG4322
Bonobo LG – LG4314
Bonobo LG – LG4300
Bonobo KR + KR10
Bonobo KR – KR67
Bonobo KR – KR57
Bonobo KR – KR52
Bonobo KR – KR35
Bonobo KR – KR33
Bonobo KR – KR21
Bonobo KR – KR12
Bonobo KR – KR07
Bonobo KR – KR02
Bonobo IK – IK4184
Bonobo IK – IK3777
Bonobo IK – IK3701
Bonobo IK – IK3650
Bonobo IK – IK3513
Bonobo IK – IK3469
Bonobo IK – IK3358
Bonobo IK – IK3276
Bonobo IK – IK3158
Dialium guineense #160 p=0.955
Dialium guineense #1 p=0.482
Dialium guineense #21 p=0.955
Dialium guineense #22 p=0.955
Dialium guineense #2500 p=0.955
Dialium guineense #2517 p=0.955
Dialium guineense #2569 p=0.955
Dialium guineense #2653 p=0.955
Dialium guineense #2693 p=0.955
Dialium guineense #28 p=1.000
Dialium guineense #71 p=0.955
Dialium guineense #72 p=0.955
Dialium guineense #75 p=0.955
Dialium guineense #76 p=0.955
Dialium guineense #77 p=0.955
Dialium guineense #80 p=0.955
Dialium guineense #81 p=0.955
Dialium guineense #83 p=0.955
Ficus carica #1034 p=1.000
Ficus carica #1216 p=0.955
Ficus carica #1229 p=1.000
Ficus carica #1 p=1.000
Ficus carica #216 p=1.000
Ficus carica #340 p=1.000
Ficus carica #3 p=1.000
Ficus carica #4 p=1.000
Ficus carica #5 p=1.000
Ficus carica #6 p=1.000
Ficus carica #7 p=1.000
Pyrenacantha grandifolia #1747 p=0.955
Pyrenacantha grandifolia #1753 p=0.955

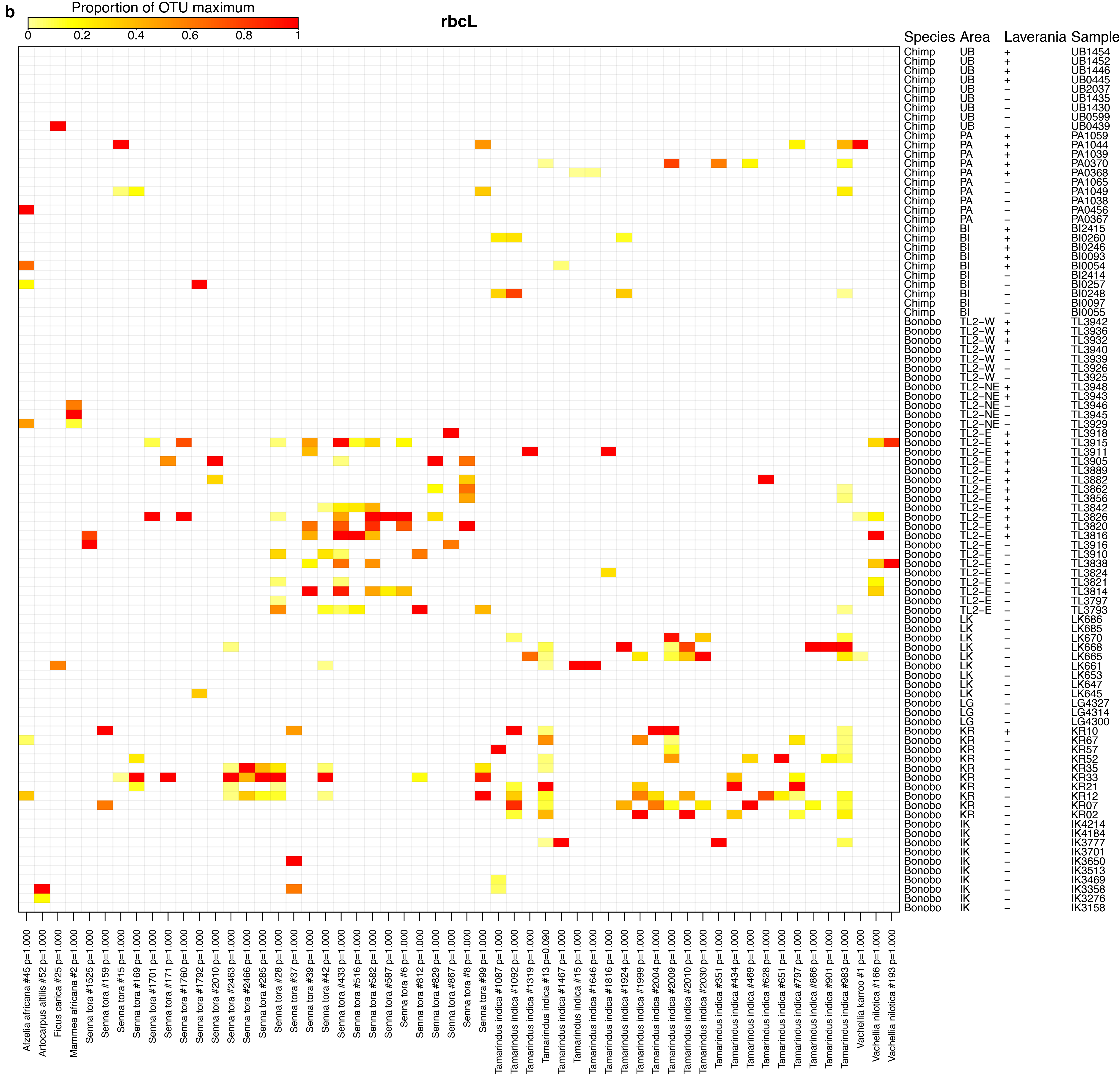
b
Proportion of OTU maximum
rbcL
Species Area Laverania Sample

Supplementary Figure 6 Relative abundance of potentially antimalarial plants in ape faecal samples. (**a**) A heatmap of operational taxonomic units (OTUs) as determined by faecal *matK* sequencing is shown. Each grid entry represents the abundance of an OTU (column) within a sample (row) relative to the maximum proportional abundance for that OTU. Only OTUs that matched a putative antimalarial plant (Supplementary Table 7) with >95% sequence identity and were found in more than one sample are shown. Samples are labeled by ape species (chimpanzee and bonobo), field site (see Fig. 5b for the location of TL2-W, TL2-E and TL2-NE samples), as well as with a + and – prefix to indicate *Laverania* positive and negative status. OTUs are labeled according to their assigned taxonomy followed by an arbitrary ID number and the false discovery rate (FDR) corrected p-value for a comparison between the endemic and non-endemic bonobo field sites. **(b)** Heatmap of OTUs as in (**a**) but for *rbcL* sequences.

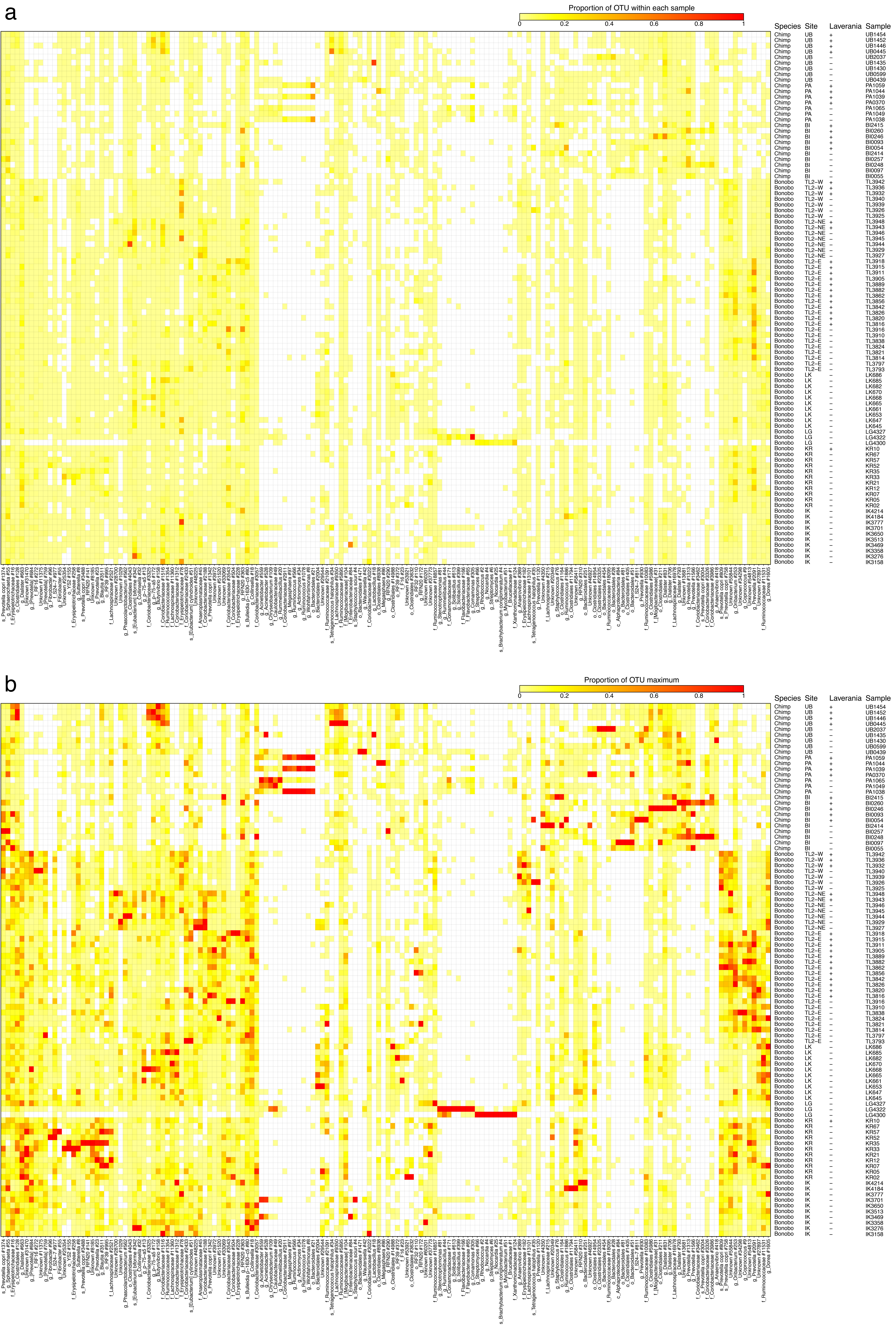

a
Proportion of OTU within each sample
0
0.2
0.4
0.6
0.8
1
Species
Site
Laverania
Sample
b
Proportion of OTU maximum
0
0.2
0.4
0.6
0.8
1
Species
Site
Laverania
Sample

Supplementary Figure 7 Relative abundance of major bacterial phyla in *Laverania* positive and negative ape faecal samples. (**a**) A heatmap of operational taxonomic units (OTUs) as determined by 16S rRNA gene sequencing is shown. Each grid entry represents the relative abundance of an OTU (column) within each sample (row). Only OTUs with an abundance of greater than 2% in at least one sample are shown. OTUs are ordered by co-occurrence to emphasise patterns in the data. Samples are labeled by ape species (chimpanzee and bonobo), field site (see Fig. 5b for the location of TL2-W, TL2-E and TL2-NE samples), as well as with a + and – prefix to indicate *Laverania* positive and negative status, respectively (see Supplementary Table 6 for a description of all samples). OTUs are labeled according to the most specific taxonomic rank assigned, with letters indicating order (o), family (f), genus (g), and species (s), followed by an arbitrary ID number. Bacterial taxa that could not be classified are labeled "Unknown". **(b)** Heatmap of OTUs as in (**a**) but with taxon abundance measured relative to the maximum proportion observed within that OTU (red cells indicate samples with the highest proportional abundance of the corresponding OTU).

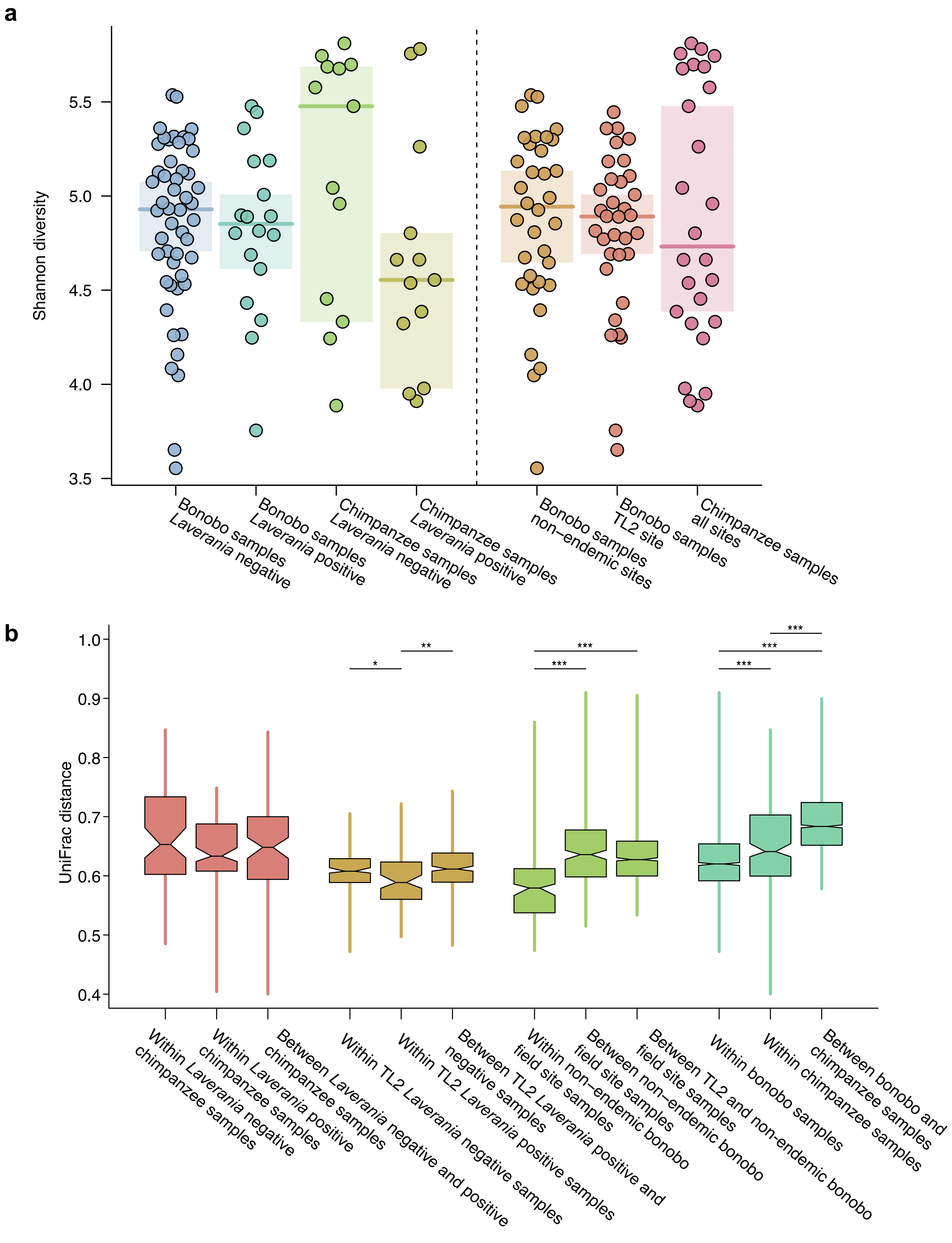
a
Shannon diversity
3.5
4.0
4.5
5.0
5.5
Bonobo samples Laverania negative
Bonobo samples Laverania positive
Chimpanzee samples Laverania negative
Chimpanzee samples Laverania positive
Bonobo samples non−endemic sites
Bonobo samples TL2 site
Chimpanzee samples all sites
b
UniFrac distance
0.4
0.5
0.6
0.7
0.8
0.9
1.0
*
**
***
Within Laverania negative chimpanzee samples
Within Laverania positive chimpanzee samples
Between Laverania negative and positive chimpanzee samples
Within TL2 Laverania negative samples
Within TL2 Laverania positive samples
Between TL2 Laverania positive and negative samples
Within non−endemic bonobo field site samples
Between non−endemic bonobo field site samples
Between TL2 and non−endemic bonobo field site samples
Within bonobo samples
Within chimpanzee samples
Between bonobo and chimpanzee samples

c

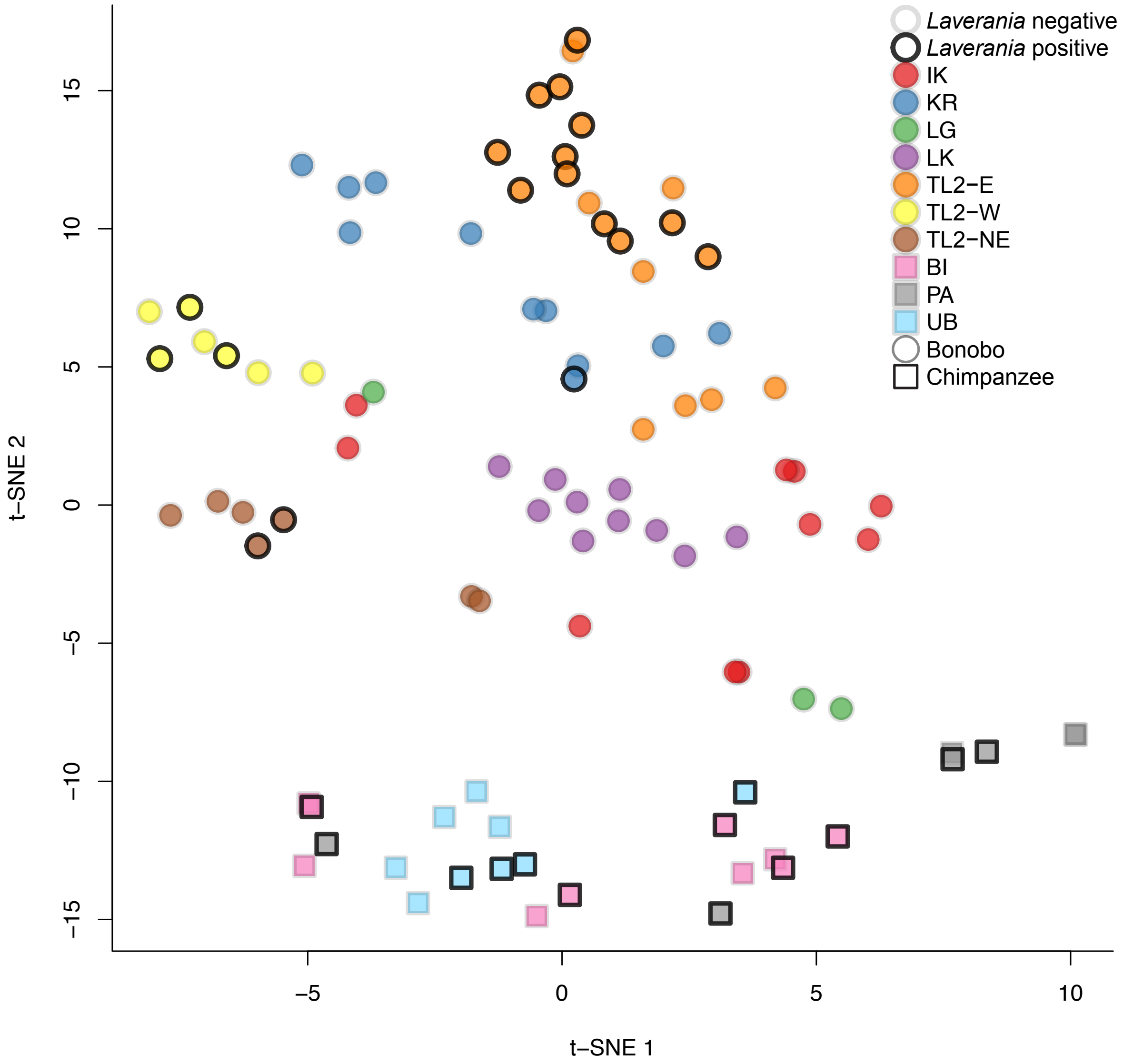

Laverania negative
Laverania positive
IK
KR
LG
LK
TL2−E
TL2−W
TL2−NE
BI
PA
UB
Bonobo
Chimpanzee
t−SNE 2
t−SNE 1
15
10
5
0
−5
−10
−15
−5
0
5
10

Supplementary Figure 8 Compositional analysis of the faecal microbiome in *Laverania* positive and negative bonobos and chimpanzees. (**a**) Analysis of alpha diversity. A comparison of the Shannon diversity for *Laverania* positive and negative faecal samples from bonobos and chimpanzees (left of dashed line), as well as *Laverania* endemic (TL2) and non-endemic (IK, KR, LG, LK) bonobo field sites, and all chimpanzee sites (right of dashed line) is shown. Each point indicates a faecal sample. Horizontal lines indicate the median within each grouping and shaded regions the 95% confidence interval of the median. Note that each sample is shown once to the left and again to the right of the dashed line (see Supplementary Table 6 for a description of all samples). There were no significant differences between the Shannon diversity medians for any of the groups shown. (**b**) Analysis of beta diversity. The distribution of rarefied unweighted UniFrac distances within and between faecal microbiomes of various groupings of apes is shown (“within non-endemic field sites" shows only comparisons between samples from the same field site, while "between non-endemic field sites" shows comparisons between samples from different non-endemic field sites). Boxes indicate the interquartile range of UniFrac distances for that grouping, and whiskers extend to minimum and maximum values. Horizontal lines indicate the median and notches show the 95% confidence interval of the median. Asterisks indicate Wilcoxon rank-sum test p-values <0.01 (*), $p<0.0001$ (**) and $p<0.000001$ (***). For comparisons of within- to between-group beta diversity (comparisons between the first and second box in each subset to the third box), a one-sided Wilcoxon test was used to search for larger between-group than within-group diversity. (**c**) A two-dimensional representation of the unweighted UniFrac distances generated using a t-distributed stochastic neighbor embedding (t-SNE), comparing faecal bacteriomes of bonobos (circles) and chimpanzees (squares) from various study sites (indicated by colour). Faecal samples that tested positive for *Laverania* DNA are circled. The sampling location of groups of TL2 samples labeled TL2-E, TL2-NE and TL2-W are shown in Fig. 5b (sample GPS coordinates are listed in Supplementary Table 6).

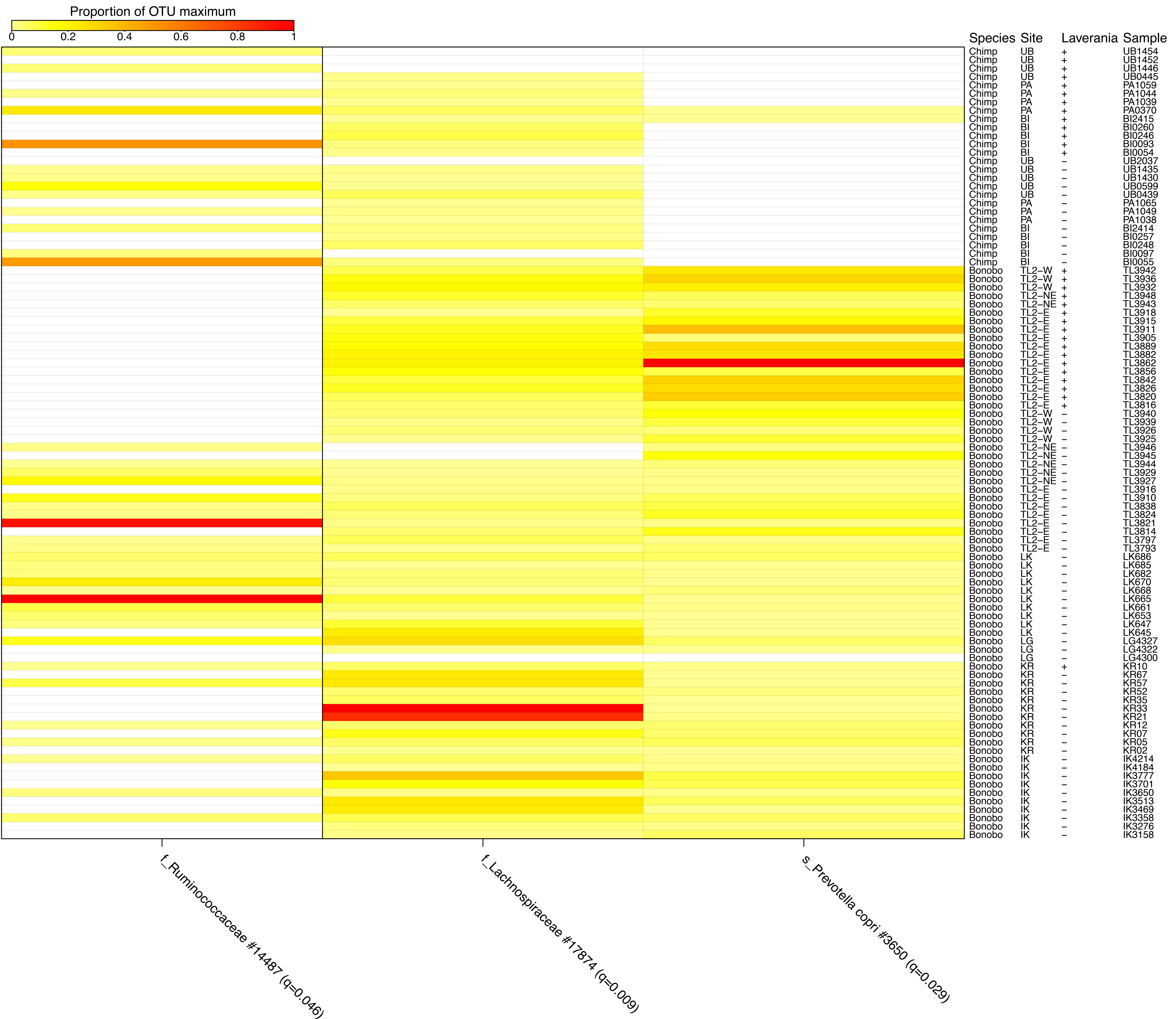
Proportion of OTU maximum
0
0.2
0.4
0.6
0.8
1
Species Site Laverania Sample
Chimp UB + UB1454
Chimp UB + UB1452
Chimp UB + UB1446
Chimp UB + UB0445
Chimp PA + PA1059
Chimp PA + PA1044
Chimp PA + PA1039
Chimp PA + PA0370
Chimp BI + BI2415
Chimp BI + BI0260
Chimp BI + BI0246
Chimp BI + BI0093
Chimp BI + BI0054
Chimp UB – UB2037
Chimp UB – UB1435
Chimp UB – UB1430
Chimp UB – UB0599
Chimp UB – UB0439
Chimp PA – PA1065
Chimp PA – PA1049
Chimp PA – PA1038
Chimp BI – BI2414
Chimp BI – BI0257
Chimp BI – BI0248
Chimp BI – BI0097
Chimp BI – BI0055
Bonobo TL2–W + TL3942
Bonobo TL2–W + TL3936
Bonobo TL2–W + TL3932
Bonobo TL2–NE + TL3948
Bonobo TL2–NE + TL3943
Bonobo TL2–E + TL3918
Bonobo TL2–E + TL3915
Bonobo TL2–E + TL3911
Bonobo TL2–E + TL3905
Bonobo TL2–E + TL3889
Bonobo TL2–E + TL3882
Bonobo TL2–E + TL3862
Bonobo TL2–E + TL3856
Bonobo TL2–E + TL3842
Bonobo TL2–E + TL3826
Bonobo TL2–E + TL3820
Bonobo TL2–E + TL3816
Bonobo TL2–W – TL3940
Bonobo TL2–W – TL3939
Bonobo TL2–W – TL3926
Bonobo TL2–W – TL3925
Bonobo TL2–NE – TL3946
Bonobo TL2–NE – TL3945
Bonobo TL2–NE – TL3944
Bonobo TL2–NE – TL3929
Bonobo TL2–NE – TL3927
Bonobo TL2–E – TL3916
Bonobo TL2–E – TL3910
Bonobo TL2–E – TL3838
Bonobo TL2–E – TL3824
Bonobo TL2–E – TL3821
Bonobo TL2–E – TL3814
Bonobo TL2–E – TL3797
Bonobo TL2–E – TL3793
Bonobo LK – LK686
Bonobo LK – LK685
Bonobo LK – LK682
Bonobo LK – LK670
Bonobo LK – LK668
Bonobo LK – LK665
Bonobo LK – LK661
Bonobo LK – LK653
Bonobo LK – LK647
Bonobo LK – LK645
Bonobo LG – LG4327
Bonobo LG – LG4322
Bonobo LG – LG4300
Bonobo KR + KR10
Bonobo KR – KR67
Bonobo KR – KR57
Bonobo KR – KR52
Bonobo KR – KR35
Bonobo KR – KR33
Bonobo KR – KR21
Bonobo KR – KR12
Bonobo KR – KR07
Bonobo KR – KR05
Bonobo KR – KR02
Bonobo IK – IK4214
Bonobo IK – IK4184
Bonobo IK – IK3777
Bonobo IK – IK3701
Bonobo IK – IK3650
Bonobo IK – IK3513
Bonobo IK – IK3469
Bonobo IK – IK3358
Bonobo IK – IK3276
Bonobo IK – IK3158
f_Ruminococcaceae #14487 (q=0.046)
f_Lachnospiraceae #17874 (q=0.009)
s_Prevotella copri #3650 (q=0.029)

Supplementary Figure 9 Enrichment and depletion of bacterial taxa in *Laverania* positive faecal samples from TL2 bonobos. The abundances of all OTUs with a Benjamini-Hochberg corrected p-value less than 0.05 for a Wilcoxon rank sum test between *Laverania* positive and negative faecal samples of TL2 bonobos are shown. Each grid entry represents the abundance of an OTU (column) within a sample (row) measured relative to the maximum proportional abundance observed within that OTU. Wilcoxon rank sum tests were performed only for samples from TL2, but all other bonobo and chimpanzee samples are shown for comparison. Samples are labeled by ape species (chimpanzee and bonobo), field site (see Fig. 5b for the location of TL2-W, TL2-E and TL2-NE samples), as well as with a + and – prefix to indicate *Laverania* positive and negative status (see Supplementary Table 6 for a description of all samples). OTU names are assigned as their most specific taxonomic classification, with a letter representing taxonomic rank (f, family; s, species) and an arbitrary ID number. The false discovery rate (FDR)-corrected p-value for each OTU is shown in parenthesis.

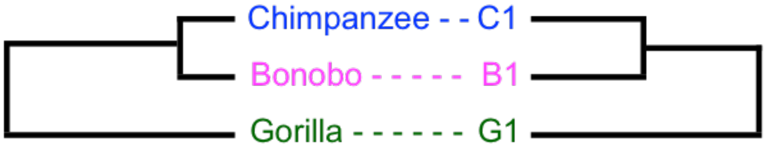

Supplementary Figure 10 Concordant phylogenetic relationships among apes and their parasites. Matching topologies are shown for chimpanzees, bonobos and gorillas on the left and their respective *Laverania* parasites *P. reichenowi* (C1), *P. lomamiensis* (B1), and *P. praefalciparum* (G1) on the right. The phylogenetic relationships are consistent with long-term co-evolution and co-divergence of this *Laverania* parasite clade with their hosts. Under this scenario, the last common ancestor of C1 and B1 existed at about the same time as the last common ancestor of chimpanzees and bonobos (around 2 Myr ago), and the last common ancestor of C1/B1 and G1 existed at about the same time as the last common ancestor of chimpanzees and gorillas (around 8-9 Myr ago; dates were taken from ref. 6). The branch lengths in the host tree reflect the time depth of their divergence; the branch lengths in the parasite tree are similar to those seen in Figs. 2 and 3. The difference in relative branch lengths indicates either that parasite molecular clock rates have varied, or that the perceived rate of divergence varies with time depth[8]. It should be emphasised that the co-divergence of parasites and their hosts does not extend to the chimpanzee subspecies level. Although C1 mitochondrial sequences segregate into two geographic clades (Fig. 2, Supplementary Figs. 1, 2a and 2b) -- one (C1E) found almost exclusively in eastern chimpanzees (*P. t. schweinfurthii*) and the other (C1W) identified in central and Nigerian/Cameroonian chimpanzees (*P. t. troglodytes* and *P. t. ellioti*) -- no such subdivision is evident in phylogenies from nuclear genes (Fig. 3; see also ref. 7). Thus, the geographic subclades comprise a single *Laverania* species, with no basis for a further subdivision of C1.

Supplementary Table 1. Intensified PCR of bonobo and chimpanzee faecal samples

Sample[a]	Host	Date (m/d/y)	Intensified *cytB* PCR		
			Number of replicates	Number of positives	Distinguishable *cytB* haplotypes
TL2.3888 (ID04)	*P. paniscus*	12/12/12	8	1	1
TL2.3843 (ID05)	*P. paniscus*	11/26/12	8	1	1
TL2.3812 (ID06)	*P. paniscus*	11/19/12	8	3	1
TL2.3846 (ID15)	*P. paniscus*	11/26/12	10	3	1
TL2.3882 (ID15)	*P. paniscus*	12/12/12	8	2	1
TL2.3826 (ID18)	*P. paniscus*	11/19/12	8	1	1
TL2.3866 (ID18)	*P. paniscus*	11/28/12	8	1	1
TL2.3874 (ID18)	*P. paniscus*	11/28/12	8	1	1
TL2.3873 (ID20)	*P. paniscus*	11/28/12	8	1	1
TL2.3842 (ID27)	*P. paniscus*	11/26/12	8	5	2
TL2.3856 (ID43)	*P. paniscus*	11/28/12	8	2	2
TL2.3862 (ID46)	*P. paniscus*	11/28/12	10	2	1
TL2.3943 (ID49)	*P. paniscus*	02/13/13	8	1	1
TL2.3918 (ID50)	*P. paniscus*	01/11/13	8	2	1
TL2.3931 (ID60)	*P. paniscus*	02/15/13	10	1	1
TL2.3936 (ID61)	*P. paniscus*	02/15/13	8	1	1
TL2.3942 (ID63)	*P. paniscus*	02/15/13	8	1	1
KRpp10 (ID28)	*P. paniscus*	10/21/06	8	1	1
BIpts54	*P. t. schweinfurthii*	03/15/03	8	2	2
BIpts67	*P. t. schweinfurthii*	n/a	8	4	2
BIpts93	*P. t. schweinfurthii*	03/15/03	8	3	2
BIpts260	*P. t. schweinfurthii*	07/25/05	8	2	2
ENpts4388	*P. t. schweinfurthii*	03/18/16	8	1	1
KSpts201	*P. t. schweinfurthii*	12/15/04	8	2	2
LUpts2029	*P. t. schweinfurthii*	06/30/07	8	1	1
LUpts2084	*P. t. schweinfurthii*	08/20/07	8	2	2
PApts75	*P. t. schweinfurthii*	05/27/03	8	1	1
PApts1039	*P. t. schweinfurthii*	09/12/06	8	1	1
PApts1040	*P. t. schweinfurthii*	09/12/06	8	1	1
PApts1041	*P. t. schweinfurthii*	09/12/06	8	2	2
PApts1042	*P. t. schweinfurthii*	09/12/06	8	2	2
PApts1044	*P. t. schweinfurthii*	09/12/06	8	2	2
PApts1053	*P. t. schweinfurthii*	09/15/06	8	1	1
PApts1054	*P. t. schweinfurthii*	09/15/06	8	4	1
PApts1056	*P. t. schweinfurthii*	09/15/06	8	2	2
PApts1058	*P. t. schweinfurthii*	12/02/06	8	1	1
PApts1059	*P. t. schweinfurthii*	12/02/06	8	2	2
PApts1060	*P. t. schweinfurthii*	12/02/06	8	1	1
PApts1061	*P. t. schweinfurthii*	12/02/06	8	1	1
PApts1062	*P. t. schweinfurthii*	12/02/06	8	1	1
PApts1064	*P. t. schweinfurthii*	12/06/06	8	1	1
WApts01	*P. t. schweinfurthii*	02/22/03	8	6	1
WApts07	*P. t. schweinfurthii*	n/a	8	1	1
WApts41	*P. t. schweinfurthii*	03/03	8	2	2
WApts394	*P. t. schweinfurthii*	12/24/05	8	2	1
WApts396	*P. t. schweinfurthii*	12/24/05	8	1	1
WApts397	*P. t. schweinfurthii*	12/04/05	8	6	4
WApts467	*P. t. schweinfurthii*	03/03/06	8	1	1
WApts469	*P. t. schweinfurthii*	03/04/06	8	1	1
WApts520	*P. t. schweinfurthii*	03/19/06	8	1	1
WApts522	*P. t. schweinfurthii*	03/20/06	8	1	1
WApts527	*P. t. schweinfurthii*	03/21/06	8	2	2
WApts529	*P. t. schweinfurthii*	03/24/06	8	1	1
WApts530	*P. t. schweinfurthii*	03/24/06	8	1	1
WApts531	*P. t. schweinfurthii*	03/24/06	8	2	2
WApts548	*P. t. schweinfurthii*	03/29/06	8	2	2
WApts555	*P. t. schweinfurthii*	03/30/06	8	1	1
WApts561	*P. t. schweinfurthii*	03/30/06	8	1	1
WLpts99	*P. t. schweinfurthii*	02/27/04	8	1	1
WLpts103	*P. t. schweinfurthii*	03/01/04	8	2	2
WLpts104	*P. t. schweinfurthii*	03/03/04	8	1	1
WLpts120	*P. t. schweinfurthii*	04/26/04	8	4	1
WLpts125	*P. t. schweinfurthii*	04/29/04	8	1	1
WLpts128	*P. t. schweinfurthii*	04/29/04	8	1	1

[a]Only samples that were found negative by conventional *cytB* PCR were subjected to intensified PCR, which entailed testing 8 to 10 aliquots of the same DNA preparation.

Supplementary Table 2. *Plasmodium* species detected in bonobo faecal samples

Sample[a]	Date (m/d/y)	Collection location	*cytB*[b] No. (hap)[d]	*cytB*[b] *P. spp.*[e]	3.4kb[b] No. (hap)[d]	3.4kb[b] *P. spp.*[e]	3.3kb[b] No. (hap)[d]	3.3kb[b] *P. spp.*[e]	*eba165*[b] No. (hap)[d]	*eba165*[b] *P. spp.*[e]	*eba*175[b] No. (hap)[d]	*eba*175[b] *P. spp.*[e]	*p47*[b] No. (hap)[d]	*p47*[b] *P. spp.*[e]	*clpM*[b] No. (hap)[d]	*clpM*[b] *P. spp.*[e]	int PCR *cox1*[c] No (hap)[d]	int PCR *cox1*[c] *P. spp.*[e]
TL2.3888 (ID04)*	12/12/12	TL2-E	5 (2)	B1, C2											1 (1)	B1		
TL2.3843 (ID05)*	11/26/12	TL2-E	7 (4)	B1, C2											1 (1)	B1		
TL2.3812 (ID06)*	11/19/12	TL2-E	3 (1)	B1											1 (1)	Pv-like	1 (1)	Pv-like
TL2.3905 (ID06)	11/26/12	TL2-E	3 (2)	B1, C2							1 (1)	B1			1 (1)	C2		
TL2.3911 (ID07)	01/08/13	TL2-E	9 (3)	B1, C2	1 (1)	B1	4 (4)	B1, C2	4 (4)	B1, C2	13 (9)	C1, B1, C2	1 (1)	B1	2 (2)	B1, C2		
TL2.3912 (ID07)	01/08/13	TL2-E	2 (1)	B1	1 (1)	B1			2 (2)	B1, C2					3 (3)	B1, C2		
TL2.3870 (ID13)	11/28/12	TL2-E	14 (8)	B1, C2	2 (2)	B1	2 (2)	B1, C2	1 (1)	B1					2 (2)	B1, C2		
TL2.3846 (ID15)*	11/26/12	TL2-E	3 (1)	B1														
TL2.3882 (ID15)*	12/12/12	TL2-E	2 (1)	B1											3 (2)	B1, C2		
TL2.3826 (ID18)*	11/19/12	TL2-E	3 (2)	B1, C2														
TL2.3866 (ID18)*	11/28/12	TL2-E	3 (2)	B1, C2											1 (1)	B1		
TL2.3874 (ID18)*	11/28/12	TL2-E	1 (1)	B1											1 (1)[f]	Pv-like	1 (1)	Po-like
TL2.3853 (ID20)	11/21/12	TL2-E															1 (1)	Pv-like
TL2.3873 (ID20)*	11/28/12	TL2-E	2 (2)	B1, C2														
TL2.3816 (ID24)	11/19/12	TL2-E	10 (3)	B1, C2	4 (4)	B1, C2	3 (2)	B1	2 (2)	B1	2 (1)	B1			3 (2)	B1, C2		
TL2.3834 (ID24)	11/26/12	TL2-E	8 (4)	B1, C2	3 (2)	B1, C2	1 (1)	B1			3 (3)	B1, C2			3 (3)	B1, C2		
TL2.3850 (ID24)	12/12/12	TL2-E	7 (3)	B1, C2	2 (1)	C2	5 (4)	B1, C2							1 (1)	B1		
TL2.3948 (ID25)	02/13/13	TL2-NE	7 (2)	B1, C2	7 (4)	B1, C2	1 (1)	B1							5 (1)	B1		
TL2.3842 (ID27)*	11/26/12	TL2-E	9 (2)	B1, C2											1 (1)	Pv-like		
TL2.3889 (ID33)	12/12/12	TL2-E	1 (1)	B1									2 (2)	B1				
TL2.3884 (ID37)	11/26/12	TL2-E	5 (1)	C2							1 (1)	C2			1 (1)	C2		
TL2.3833 (ID39)	11/19/12	TL2-E	2 (2)	C2	1 (1)	B1	1 (1)	B1							1 (1)	C2		
TL2.3915 (ID41)	01/08/13	TL2-E	4 (2)	B1, C2					2 (2)	B1			1 (1)	C2	1 (1)	C2		
TL2.3856 (ID43)*	11/28/12	TL2-E	2 (2)	B1, C2											1 (1)	C2		
TL2.3820 (ID45)	11/19/12	TL2-E	12 (7)	B1, C2	6 (3)	B1	5 (3)	B1					1 (1)	B1	1 (1)	B1		
TL2.3903 (ID45)	12/13/12	TL2-E	2 (1)	C2											3 (3)	B1, C2		
TL2.3862 (ID46)*	11/28/12	TL2-E	10 (4)	B1, C2, Pm-related											2 (2)	B1, C2		
TL2.3855 (ID47)	11/28/12	TL2-E	6 (2)	B1, C2			1 (1)	B1					1 (1)	B1	1 (1)	B1		
TL2.3943 (ID49)*	02/13/13	TL2-NE	1 (1)	B1														
TL2.3918 (ID50)*	01/11/13	TL2-E	4 (2)	B1											1 (1)	B1		
TL2.3931 (ID60)*	02/15/13	TL2-W	3 (2)	B1, C2											3 (2)	B1, C2		
TL2.3932 (ID60)	02/15/13	TL2-W	5 (2)	B1, C2	2 (2)	B1, C2												
TL2.3936 (ID61)*	02/15/13	TL2-W	2 (2)	B1, C2											1 (1)	B1		
TL2.3942 (ID63)*	02/15/13	TL2-W	6 (2)	B1, C2											1 (1)	B1		
KRpp10 (ID28)*	10/21/06	KR	3 (1)	B1											2 (1)	B1		

[a]Samples are labeled to indicate their field site of origin (TL2 and KR; see Figs. 1 and 5b for location) followed by an individual (ID) number as determined by microsatellite analysis at 8 polymorphic loci[9]. Asterisks indicate *Laverania* positive samples that were identified by intensified PCR.
[b]Single genome amplified loci of *Plasmodium* mitochondrial (*cytB,* 3.4 kb, 3.3 kb), nuclear (*eba165, eba175* and *p47*) and apicoplast (*clpM*) genes. For samples TL2.3812, TL2.3846, TL2.3856, TL2.3873, TL2.3874, TL2.3882 and TL2.3943, *cytB* sequences were derived from the intensified PCR screen. All of the respective sequences lacked double peaks, indicating that they were single template-derived.
[c]int PCR, intensified PCR targeting a 296 bp mitochondrial *cox1* fragment using *P. vivax*-specific primers.
[d]No., number of single template derived sequences (SGA or intensified PCR), with brackets indicating the number of distinguishable haplotypes (hap). See Supplementary Data 1 for GenBank accession numbers.
[e]*Plasmodium* species present in the faecal sample: B1, *P. lomamiensis*; C1, *P. reichenowi*; C2, *P. gaboni*; Pm-related, *P. malariae*-related; Po-like, *P. ovale*-like; Pv-like, *P. vivax*-like.
[f]Obtained using *P. vivax*-specific *clpM* primers as previously described[9].

Supplementary Table 3. Faecal based screening of eastern chimpanzees for *Laverania* infections

Field Sites[b]	Conventional *cytB* screen			Intensified *cytB* screen[a]			Combined detection rate (%)
	Samples tested[c]	Samples positive[d]	Detection rate (%)	Samples tested[c]	Samples positive[d]	Detection rate (%)	
Amunyala (AM)	37	0	0	23[e]	0	0	0
Azunu (AZ)	31	0	0	31	0	0	0
Babingi (BI)	96	7	7.3	89	4	4.5	11.5
Engali (EN)	26	0	0.0	26	1	3.8	3.8
Kisangani (KS)	11	0	0.0	11	1	9.1	9.1
Lubutu (LU)	131	10	7.6	117[e]	2	1.7	9.4
Parisi (PA)	77	10	13.0	67	15	22.4	32.5
Wanie-rukula (WA)	134	13	9.7	121	17	14.0	22.4
Walengola (WL)	37	5	13.5	32	6	18.8	29.7

[a]Samples initially negative in the conventional (diagnostic) *cytB* screen were subsequently tested by intensified PCR, performing 8 to 10 independent amplification reactions per faecal DNA (see Supplementary Table 1 for details).
[b]Field sites are designated by a two-letter code (their location is shown in Fig. 1).
[c]The host species origin of all faecal samples was confirmed by mitochondrial DNA (D loop) analysis; for most of these sites, regular *cytB* screening results have previously been reported[10].
[d]All amplification products were sequence confirmed to represent *Laverania* parasites (see Supplementary Table 4).
[e]Only a subset of the originally screened AM and LU samples was available for intensified PCR.

Supplementary Table 4. *Laverania* species in eastern chimpanzee faecal samples collected most proximal to the bonobo range

No.	Sample code[a]	Collection date[b]	*P. t. schweinfurthii* mtDNA haplotype[c]	*cytB* PCR[d] No. (hap)[e]	*cytB* PCR[d] *P. spp.*[f]
1	BIpts54	03/15/03	DQ370342	2 (2)*	C2
2	BIpts67	n/a	DQ370345	4 (2)*	C2
3	BIpts93	03/15/03	DQ370346	2 (2)*	C2
4	BIpts244	05/16/05	DQ370333	1 (1)	C2
5	BIpts245	05/17/05	DQ370333	1 (1)	C2
6	BIpts246	05/19/05	DQ370333	1 (1)	C2
7	BIpts253	07/10/05	JQ866111	1 (1)	C2
8	BIpts260	07/25/05	JQ866111	2 (2)*	C2
9	BIpts266	07/27/05	JQ866111	1 (1)	C2
10	BIpts2415	04/21/07	EU527448	2 (2)	C2
11	BIpts2416	04/21/07	EU527448	1 (1)	C2
12	ENpts4388	03/18/16	JQ866157	1 (1)*	C1
13	KSpts201	12/15/04	JQ866156	2 (2)*	C2
14	LUpts2029	06/30/07	JQ866180	1 (1)*	C1
15	LUpts2067	08/09/07	JQ866096	1 (1)	C2
16	LUpts2069	08/09/07	DQ370340	1 (1)	C2
17	LUpts2070	08/09/07	DQ370342	1 (1)	C2
18	LUpts2071	08/13/07	DQ370342	7 (4)	C2
19	LUpts2072	08/13/07	DQ370342	1 (1)	C2
20	LUpts2073	08/13/07	JQ866096	7 (3)	C1, C2
21	LUpts2074	08/15/07	JQ866096	1 (1)	C2
22	LUpts2078	08/15/07	DQ370342	1 (1)	C2
23	LUpts2079	08/15/07	JQ866096	1 (1)	C2
24	LUpts2084	08/20/07	DQ370332	2 (2)*	C1, C2
25	LUpts2089	08/20/07	DQ370332	1 (1)	C3
26	PApts75	05/27/03	DQ370340	1 (1)*	C1
27	PApts369	01/04/06	JQ866214	1 (1)	C1
28	PApts370	01/04/06	JQ866214	4 (2)	C1, C2
29	PApts1039	09/12/06	JQ866206	1 (1)*	C3
30	PApts1040	09/12/06	JQ866206	1 (1)*	C2
31	PApts1041	09/12/06	JQ866206	2 (2)*	C1, C3
32	PApts1042	09/12/06	JQ866206	2 (2)*	C1, C3
33	PApts1043	09/12/06	JQ866206	1 (1)	C1
34	PApts1044	09/12/06	JQ866205	2 (2)*	C2, C3
35	PApts1046	09/15/06	JQ866205	3 (3)	C2, C3
36	PApts1047	09/15/06	JQ866205	3 (3)	C2, C3
37	PApts1048	09/15/06	JQ866205	2 (2)	C3
38	PApts1052	09/15/06	JQ866205	4 (3)	C2, C3
39	PApts1053	09/15/06	JQ866205	1 (1)*	C1
40	PApts1054	09/15/06	JQ866205	3 (1)*	C2
41	PApts1056	09/15/06	JQ866205	2 (2)*	C1, C2
42	PApts1058	12/02/06	JQ866206	1 (1)*	C1
43	PApts1059	12/02/06	JQ866206	2 (2)*	C1, C2
44	PApts1060	12/02/06	JQ866206	1 (1)*	C3
45	PApts1061	12/02/06	JQ866206	1 (1)*	C3
46	PApts1062	12/02/06	JQ866206	1 (1)*	C1
47	PApts1063	12/02/06	JQ866206	2 (2)	C2
48	PApts1064	12/06/06	JQ866206	1 (1)*	C3
49	PApts3143	12/12/10	JQ866211	1 (1)	C2
50	PApts3146	12/13/10	JQ866211	4 (4)	C1, C2
51	WApts1	02/22/03	DQ370332	5 (1)*	C3
52	WApts2	02/22/03	DQ370332	5 (2)	C2, C3
53	WApts7	n/a	DQ370334	1 (1)*	C1
54	WApts21	n/a	DQ370342	3 (3)	C2
55	WApts22	n/a	DQ370342	2 (2)	C1, C2
56	WApts41	03/03	EU527448	2 (2)*	C3
57	WApts392	12/24/05	JQ866254	6 (3)	C2
58	WApts393	12/24/05	JQ866254	6 (3)	C2
59	WApts394	12/24/05	JQ866247	1 (1)*	C2
60	WApts395	12/24/05	JQ866247	1 (1)	C2

61	WApts396	12/24/05	JQ866247	1 (1)*	C2
62	WApts397	12/04/05	JQ866254	5 (4)*	C2
63	WApts398	12/04/05	JQ866247	1 (1)	C1
64	WApts399	12/04/05	JQ866247	5 (2)	C2
65	WApts467	03/03/06	UE527409	1 (1)*	C2
66	WApts469	03/04/06	UE527409	1 (1)*	C2
67	WApts513	03/17/06	DQ370336	2 (2)	C1, C2
68	WApts519	03/19/06	DQ370342	2 (2)	C1, C3
69	WApts520	03/19/06	DQ370342	1 (1)*	C1
70	WApts522	03/20/06	DQ370342	1 (1)*	C3
71	WApts523	03/20/06	DQ370342	1 (1)	C3
72	WApts525	03/21/06	DQ370342	2 (1)	C1
73	WApts527	03/21/06	DQ370342	2 (2)*	C1
74	WApts529	03/24/06	DQ370342	1 (1)*	C3
75	WApts530	03/24/06	DQ370342	1 (1)*	C1
76	WApts531	03/24/06	DQ370342	2 (2)*	C1
77	WApts548	03/29/06	JQ866255	2 (2)*	C1, C2
78	WApts555	03/30/06	JQ866257	1 (1)*	C1
79	WApts561	03/30/06	JQ866255	1 (1)*	C1
80	WApts563	03/30/06	JQ866257	1 (1)	C1
81	WLpts99	02/27/04	JQ866203	1 (1)*	C1
82	WLpts101	03/01/04	EU527447	5 (4)	C1, C3
83	WLpts103	03/01/04	EU527450	2 (2)*	C1, C2
84	WLpts104	03/03/04	EU527451	1 (1)*	C3
85	WLpts111	04/16/04	EU527455	2 (1)	C1
86	WLpts113	04/22/04	EU527455	5 (1)	C1
87	WLpts120	04/26/04	EU527455	5 (1)	C1
88	WLpts125	04/29/04	EU527455	1 (1)*	C1
89	WLpts128	04/29/04	EU527455	1 (1)*	C1
90	WLpts131	04/29/04	EU527455	5 (2)	C1, C2
91	WLpts132	04/29/04	EU527455	5 (4)	C1, C2

[a]Samples are labeled to indicate their field site (Fig. 1) and chimpanzee subspecies of origin (pts, *P. t. schweinfurthii*), followed by a number.
[b]Collection dates are listed by month, day and year (m/d/y); n/a, not available.
[c]Faecal samples were subjected to mitochondrial DNA analysis to confirm their host species and subspecies origin.
[d]*cytB* sequences were derived by single genome amplification or intensified PCR. Only single template derived sequences lacking double peaks in sequence chromatograms were included; see Supplementary Table 1 for additional detail.
[e]No., number of SGA or intensified PCR derived sequences (indicated by asterisks), with brackets indicating the number of distinguishable haplotypes (hap); see Supplementary Data 1 for GenBank accession numbers.
[f]Ape *Laverania* species present in the sample: C1, *P. reichenowi*; C2, *P. gaboni*; C3, *P. billcollinsi.*

Supplementary Table 5. Ecological variables at bonobo sampling sites

Site[a]	Ambient temperature[b]			Daily temperature variation[b]			Forest cover[c]	Rainfall[d] (mm/day)		
	Min	Max	Mean	Min	Max	Mean		Min	Max	Mean
BN	24.5°C	25.9°C	25.2°C	8.1°C	8.6°C	8.4°C	100%	3.0	5.7	4.1
BX	25.1°C	25.1°C	25.1°C	8.9°C	8.9°C	8.9°C	100%	7.1	7.1	7.1
BJ	24.1°C	24.1°C	24.1°C	8.7°C	8.7°C	8.7°C	98%	3.2	3.2	3.2
IK	23.9°C	26.3°C	25.0°C	6.8°C	9.2°C	8.4°C	100%	3.4	6.8	5.1
KR	23.3°C	24.5°C	24.2°C	8.8°C	9.5°C	9.2°C	100%	1.3	7.0	4.4
LG	23.5°C	25.4°C	24.2°C	6.3°C	9.3°C	8.5°C	90%	5.8	5.8	5.8
LA	23.9°C	26.3°C	25.3°C	7.1°C	9.3°C	8.3°C	93%	5.7	8.8	6.7
LK	24.8°C	25.9°C	25.3°C	9.2°C	9.6°C	9.3°C	100%	3.2	5.7	3.8
ML	25.0°C	26.3°C	25.7°C	9.0°C	9.8°C	9.2°C	47%	2.4	6.9	4.3
MZ	24.6°C	25.7°C	24.9°C	8.4°C	10.0°C	9.5°C	55%	0.7	7.0	3.0
TL2	22.2°C	25.0°C	23.8°C	8.3°C	10.3°C	8.7°C	100%	4.5	8.6	7.4

[a]Field sites are designated by a two-letter code (their location is shown in Fig. 1).
[b]Mean ambient temperature and daily temperature fluctuation were derived from MODIS LST datasets[11,12] after applying minimum and maximum air temperature transformations[13]. Each metric was calculated as the average of temperature estimates recorded during the 30 days prior to sample collection.
[c]Forest cover was derived from high resolution maps of global forest cover[14].
[d]Rainfall measurements were derived from the Global Precipitation Climatology Project (GPCP V2.3)[15] at monthly temporal resolution. We used rainfall estimates corresponding to the month during which each sample was collected.

Supplementary Table 6. Analysis of plant and microbiome constituents in *Laverania* positive and negative ape faecal samples

		Collection date		mtDNA	GPS Coordinates		*matK*	*rbcL*	16S rRNA	*Laverania*
**No.**	**Sample code**	**mm/dd/yy**	**Species**	**Haplotype[a]**	**Latitude**	**Longitude**	**Filtered reads[b]**	**Filtered reads[b]**	**Filtered reads[b]**	***cytB***
1	**TL2.3793**	10/09/12	*P. p.*	JQ866273	S2.71434°	E025.13563°	7947	15360	20998	neg
2	**TL2.3797**	10/09/12	*P. p.*	JQ866273	S2.71464°	E025.13543°	5722	20919	94860	neg
3	**TL2.3814**	11/19/12	*P. p.*	KY790554	S2.73634°	E025.11536°	23741	23378	119333	neg
4	**TL2.3816**	11/19/12	*P. p.*	KY790552	S2.73634°	E025.11536°	22541	18222	76728	**pos**
5	**TL2.3820**	11/19/12	*P. p.*	KY790554	S2.69626°	E025.13756°	15980	12506	62538	**pos**
6	**TL2.3821**	11/19/12	*P. p.*	KY790555	S2.69672°	E025.13752°	10483	18662	45145	neg
7	**TL2.3824**	11/19/12	*P. p.*	KY790552	S2.69673°	E025.13754°	14012	24745	54876	neg
8	**TL2.3826**	11/19/12	*P. p.*	KY790552	S2.69610°	E025.13711°	27873	17856	44839	**pos**
9	**TL2.3838**	11/26/12	*P. p.*	KY790555	S2.69751°	E025.13761°	17244	20513	79467	neg
10	**TL2.3842**	11/26/12	*P. p.*	KY790555	S2.69776°	E025.13740°	16785	25140	73647	**pos**
11	**TL2.3856**	11/28/12	*P. p.*	KY790554	S2.69353°	E025.13725°	17809	26893	51001	**pos**
12	**TL2.3862**	11/28/12	*P. p.*	KY790554	S2.69321°	E025.13744°	16211	29616	47867	**pos**
13	**TL2.3882**	12/12/12	*P. p.*	KY790556	S2.67151°	E025.14455°	8264	18803	85344	**pos**
14	**TL2.3889**	12/12/12	*P. p.*	KY790553	S2.67129°	E025.14424°	17953	15526	78724	**pos**
15	**TL2.3905**	11/26/12	*P. p.*	JQ866273	S2.69218°	E025.13.760°	9894	19656	61606	**pos**
16	**TL2.3910**	10/09/12	*P. p.*	JQ866273	S2.71429°	E025.13545°	8892	20340	52017	neg
17	**TL2.3911**	01/08/13	*P. p.*	JQ866273	S 2.71764°	E25.13718°	6004	20126	47547	**pos**
18	**TL2.3915**	01/08/13	*P. p.*	KY790554	S 2.71777°	E25.13698°	12350	24011	98946	**pos**
19	**TL2.3916**	01/11/13	*P. p.*	KY790550	S 2.73126°	E25.14773°	4	14324	75531	neg
20	**TL2.3918**	01/11/13	*P. p.*	KY790557	S 2.73117°	E25.14776°	5461	6455	97977	**pos**
21	**TL2.3925**	02/14/13	*P. p.*	KY790561	S2.73794°	E25.08592°	17959	13637	60840	neg
22	**TL2.3926**	02/14/13	*P. p.*	KY790562	S2.73790°	E25.08533°	14300	17784	52793	neg
23	**TL2.3927**	02/13/13	*P. p.*	JQ866273	S2.46740°	E25.10224°	7431	4185	31964	neg
24	**TL2.3929**	02/13/13	*P. p.*	KY790558	S2.46748°	E25.10211°	9298	8936	43821	neg
25	**TL2.3932**	02/15/13	*P. p.*	KY790563	S2.73758°	E25.08219°	103020	16768	85037	**pos**
26	**TL2.3936**	02/15/13	*P. p.*	KY790563	S2.73733°	E25.08259°	8064	5543	58927	**pos**
27	**TL2.3939**	02/15/13	*P. p.*	KY790563	S2.73741°	E25.08270°	8503	21353	60304	neg
28	**TL2.3940**	02/15/13	*P. p.*	KY790564	S2.73773°	E25.08260°	4968	15543	62308	neg
29	**TL2.3942**	02/15/13	*P. p.*	KY790564	S2.73769°	E25.08246°	38565	13599	61839	**pos**
30	**TL2.3943**	02/13/13	*P. p.*	KY790557	S2.46743°	E25.10205°	27889	14550	72265	**pos**

31	**TL2.3944**	02/13/13	*P. p.*	KY790556	S2.46740°	E25.10197°	27335	2675	76918	neg
32	**TL2.3945**	02/13/13	*P. p.*	KY790560	S2.46741°	E25.10191°	29458	11265	42629	neg
33	**TL2.3946**	02/13/13	*P. p.*	KY790560	S2.46726°	E25.10183°	19068	8706	42537	neg
34	**TL2.3948**	02/13/13	*P. p.*	KY790555	S2.47118°	E25.08098°	28899	9829	46888	**pos**
35	**KR02**	09/28/06	*P. p.*	JQ866277	n/a	n/a	14646	21960	83123	neg
36	**KR05**	09/29/06	*P. p.*	JQ866282	n/a	n/a	4771	2036	81899	neg
37	**KR07**	10/21/06	*P. p.*	JQ866278	n/a	n/a	26741	33636	115307	neg
38	**KR10**	10/21/06	*P. p.*	JQ866279	n/a	n/a	21173	20332	59075	**pos**
39	**KR12**	10/28/06	*P. p.*	JQ866278	n/a	n/a	9687	25001	89109	neg
40	**KR21**	11/07/06	*P. p.*	JQ866274	n/a	n/a	15947	22351	54062	neg
41	**KR33**	12/06/06	*P. p.*	JQ866274	n/a	n/a	17148	20767	50734	neg
42	**KR35**	12/07/06	*P. p.*	JQ866282	n/a	n/a	23884	25620	76542	neg
43	**KR52**	12/19/06	*P. p.*	JQ866280	n/a	n/a	27954	12586	64347	neg
44	**KR57**	12/19/06	*P. p.*	JQ866278	n/a	n/a	7079	12881	24998	neg
45	**KR67**	01/04/07	*P. p.*	JQ866279	n/a	n/a	9515	39674	126801	neg
46	**IK3158**	03/21/11	*P. p.*	JQ866275	S01°11.200'	E023°44.790'	13214	28956	44740	neg
47	**IK3276**	03/21/11	*P. p.*	JQ866282	S01°06'41.2"	E023°36'55.4"	31517	26904	50873	neg
48	**IK3358**	04/11/11	*P. p.*	JQ866275	S01°07'47"	E023°37'25"	13822	33260	42949	neg
49	**IK3469**	07/07/11	*P. p.*	JQ866278	S01°07.724	E023°41.576	22060	26309	114142	neg
50	**IK3513**	07/09/11	*P. p.*	JQ866279	S01°07'03.4"	E023°38'34.4	23806	34224	74192	neg
51	**IK3650**	12/14/11	*P. p.*	JQ866276	S01°07'38.3"	E023°37'30.6"	23584	20059	43577	neg
52	**IK3701**	12/20/11	*P. p.*	JQ866274	S01°08.677'	E023°41.501'	33665	37953	127273	neg
53	**IK3777**	01/05/12	*P. p.*	JQ866280	S01°07.390"	E023°37.308	11927	35568	36793	neg
54	**IK4184**	10/18/14	*P. p.*	JQ866282	S01°07'43.7"	E023°39'49.9"	13565	18817	38340	neg
55	**IK4214**	11/27/14	*P. p.*	JQ866282	S01°09'19.7"	E023°37'10.2"	394	12082	116207	neg
56	**LG4300**	12/14/15	*P. p.*	JQ866292	N 00°33'21.4""	E20°45'39.2"	15492	19342	28653	neg
57	**LG4314**	12/15/15	*P. p.*	JQ866280	N 00°33'21.4""	E20°45'39.2"	14440	41723	2035	neg
58	**LG4322**	12/20/15	*P. p.*	JQ866292	N 00°34'50.7"	E20°47'51.2"	16035	233	74266	neg
59	**LG4327**	01/08/16	*P. p.*	JQ866280	N 00°34'50.7"	E20°47'51.2"	22440	40332	61003	neg
60	**LK645**	03/24/06	*P. p.*	JQ866286	n/a	n/a	16112	24076	85344	neg
61	**LK647**	03/28/06	*P. p.*	JQ866288	n/a	n/a	24322	17476	101494	neg
62	**LK653**	04/06/06	*P. p.*	JQ866287	n/a	n/a	23559	29513	96049	neg
63	**LK661**	05/05/06	*P. p.*	JQ866290	n/a	n/a	12913	39493	109686	neg
64	**LK665**	05/11/06	*P. p.*	JQ866291	n/a	n/a	30148	31452	47672	neg
65	**LK668**	05/11/06	*P. p.*	JQ866292	n/a	n/a	17831	28287	36821	neg
66	**LK670**	05/11/06	*P. p.*	JQ866286	n/a	n/a	23314	23285	104503	neg
67	**LK682**	05/19/06	*P. p.*	JQ866289	n/a	n/a	17084	3631	72794	neg

68	**LK685**	05/19/06	*P. p.*	JQ866287	n/a	n/a	6461	5684	54426	neg
69	**LK686**	05/24/06	*P. p.*	JQ866289	n/a	n/a	6786	6640	72085	neg
70	**BI0054**	03/15/03	*P. t. s.*	DQ370342	n/a	n/a	20681	47084	68351	**pos**
71	**BI0055**	03/15/03	*P. t. s.*	DQ370346	n/a	n/a	16785	8170	63608	neg
72	**BI0093**	03/15/03	*P. t. s.*	DQ370346	n/a	n/a	6138	24965	69915	**pos**
73	**BI0097**	04/25/03	*P. t. s.*	DQ370347	n/a	n/a	18241	19367	42972	neg
74	**BI0246**	05/19/05	*P. t. s.*	DQ370333	n/a	n/a	14555	43796	17592	**pos**
75	**BI0248**	07/08/05	*P. t. s.*	JQ866111	n/a	n/a	22069	27501	47217	neg
76	**BI0257**	07/14/05	*P. t. s.*	EU527455	n/a	n/a	25117	24624	80433	neg
77	**BI0260**	07/25/05	*P. t. s.*	JQ866111	n/a	n/a	11974	37047	62062	**pos**
78	**BI2414**	04/21/07	*P. t. s.*	JQ866093	n/a	n/a	22075	34955	79300	neg
79	**BI2415**	04/21/07	*P. t. s.*	EU527448	n/a	n/a	10745	42345	44487	**pos**
80	**UB0439**	01/15/06	*P. t. s.*	JQ866237	n/a	n/a	14645	23095	86804	neg
81	**UB0445**	01/18/06	*P. t. s.*	JQ866239	n/a	n/a	6465	13292	83467	**pos**
82	**UB0599**	02/12/06	*P. t. s.*	JQ866242	n/a	n/a	18716	5956	129171	neg
83	**UB1430**	01/09/07	*P. t. s.*	JQ866226	N03° 38'36	E022° 26'03	20021	50333	56542	neg
84	**UB1435**	02/14/07	*P. t. s.*	JQ866224	N03° 38'36	E022° 26'03	28602	17081	119248	neg
85	**UB1446**	02/22/07	*P. t. s.*	JQ866224	N03° 24'15	E022° 10'17	10862	52110	47583	**pos**
86	**UB1452**	02/22/07	*P. t. s.*	JQ866238	n/a	n/a	16446	13066	35664	**pos**
87	**UB1454**	02/22/07	*P. t. s.*	JQ866224	N03° 24'15	E022° 10'17	4718	14353	55044	**pos**
88	**UB2037**	04/05/07	*P. t. s.*	JQ866230	N03°38'36	E022°26'03	15020	23895	56189	neg
89	**PA0367**	12/15/05	*P. t. s.*	JQ866213	n/a	n/a	8961	12530	4527	neg
90	**PA0368**	12/15/05	*P. t. s.*	JQ866213	n/a	n/a	6949	32878	5549	**pos**
91	**PA0370**	01/04/06	*P. t. s.*	JQ866214	n/a	n/a	2121	19918	87786	**pos**
92	**PA0456**	01/30/06	*P. t. s.*	JQ866215	n/a	n/a	6184	10997	19	neg
93	**PA1038**	09/12/06	*P. t. s.*	JQ866206	n/a	n/a	12362	35343	46648	neg
94	**PA1039**	09/12/06	*P. t. s.*	JQ866206	n/a	n/a	12770	38723	54476	**pos**
95	**PA1044**	09/12/06	*P. t. s.*	JQ866205	n/a	n/a	29407	12171	111292	**pos**
96	**PA1049**	09/15/06	*P. t. s.*	JQ866205	n/a	n/a	27144	18356	87174	neg
97	**PA1059**	12/02/06	*P. t. s.*	JQ866206	n/a	n/a	17838	36066	57812	**pos**
98	**PA1065**	12/06/06	*P. t. s.*	JQ866206	n/a	n/a	15533	36026	52100	neg

[a]GenBank accession numbers of mtDNA haplotypes
[b]reads remaining after filtering expected errors to <1 and removing singleton OTUs

Supplementary Table 7. African plant species reported to have potential antimalarial activity.

No.	Plant	Family	Country/Region	References
1	*Abrus precatorius*	Fabaceae	Nigeria/South Africa	Lawal et al, 2015
2	*Abuta grandifolia*	Menispermaceae	Brazil	Silva et al, 2011
3	*Acacia karroo*	Fabaceae	Mozambique	Lawal et al, 2015
4	*Acacia erioloba*	Fabaceae	South Africa	Lawal et al, 2015
5	*Acacia nilotica*	Fabaceae	South Africa/Sudan	Lawal et al, 2015
6	*Acacia tortilis*	Fabaceae	South Africa	Lawal et al, 2015
7	*Acanthospermum australe*	Asteraceae	Brazil	Silva et al, 2011
8	*Acanthospermum hispidum*	Asteraceae	Benin/Ivory Coast/Sudan	Lawal et al, 2015
9	*Anchomanes difformis*	Araceae	Cameroon/Benin	Lawal et al, 2015
10	*Achyranthes aspera*	Amaranthaceae	South Africa	Lawal et al, 2015
11	*Acokanthera oppositifolia*	Apocynaceae	Kenya	Lawal et al, 2015
12	*Acokanthera schimperi*	Apocynaceae	Kenya	Lawal et al, 2015
13	*Adenia cissampeloides*	Passifloraceae	Ghana	Lawal et al, 2015
14	*Adenia rumicifolia*	Passifloraceae	Ghana	Lawal et al, 2015
15	*Aerva javanica*	Amaranthaceae	Sudan	Lawal et al, 2015
16	*Afzelia africana*	Fabaceae	Nigeria	Lawal et al, 2015
17	*Agathosma apiculata*	Rutaceae	South Africa	Lawal et al, 2015
18	*Agathosma puberula*	Rutaceae	South Africa	Lawal et al, 2015
19	*Ageratum conyzoides*	Asteraceae	São Tomé/South Africa	Lawal et al, 2015,<br>Silva et al, 2011
20	*Alangium chinense*	Alangiaceae	Kenya	Lawal et al, 2015
21	*Albizia ferruginea*	Fabaceae	Ivory Coast	Lawal et al, 2015
22	*Albizia versicolour*	Fabaceae	South Africa	Lawal et al, 2015
23	*Albizia zygia*	Mimosaceae	Cameroon	Lawal et al, 2015
24	*Alchornea cordifolia*	Euphorbiaceae	Congo/Ivory Coast	Lawal et al, 2015
25	*Alchornea floribunda*	Euphorbiaceae	Congo	Lawal et al, 2015
26	*Alepidea amatymbica*	Apiaceae	South Africa	Lawal et al, 2015
27	*Alhagi graecorum*	Papilionaceae	Egypt	Lawal et al, 2015
28	*Aloe ferox*	Asphodelaceae	South Africa	Lawal et al, 2015
29	*Aloe maculata*	Asphodelaceae	South Africa	Lawal et al, 2015
30	*Aloe marlothii*	Asphodelaceae	South Africa	Lawal et al, 2015
31	*Aloe parvibracteata*	Aloaceae	Mozambique	Lawal et al, 2015
32	*Alstonia boonei*	Apocynaceae	Congo/Ivory Coast	Lawal et al, 2015
33	*Alternanthera pungens*	Amaranthaceae	Nigeria	Lawal et al, 2015
34	*Amaranthus lividus*	Amaranthaceae	Egypt	Lawal et al, 2015
35	*Ambrosia maritime*	Asteraceae	Sudan	Lawal et al, 2015
36	*Ampelozizyphus amazonicus*	Rhamanaceae	Brazil	Silva et al, 2011
37	*Anacardium occidentale*	Anacardiaceae	Ivory Coast	Lawal et al, 2015
38	*Anastatica hierochuntica*	Cruciferae	Egypt	Lawal et al, 2015
39	*Andira inermis*	Fabaceae	Brazil	Silva et al, 2011
40	*Anisopappus chinensis*	Asteraceae	Congo	Lawal et al, 2015
41	*Annona muricata*	Annonaceae	Cameroon	Lawal et al, 2015
42	*Annona senegalensis*	Annonaceae	South Africa	Lawal et al, 2015
43	*Anogeissus leiocarpa*	Combrataceae	Nigeria	Lawal et al, 2015
44	*Anonidium mannii*	Annonaceae	Congo	Lawal et al, 2015
45	*Anthocleista djalonensis*	Loganiaceae	Ivory Coast	Lawal et al, 2015
46	*Anthocleista grandiflora*	Gentianaceae	South Africa	Lawal et al, 2015
47	*Anthocleista nobilis*	Loganiaceae	Burkina Faso	Lawal et al, 2015
48	*Anthonotha macrophylla*	Caesalpiniaceae	Ivory Coast	Lawal et al, 2015
49	*Aristolochia bracteolata*	Aristolochiaceae	Sudan	Lawal et al, 2015
50	*Aristolochia elegans*	Aristolochiaceae	Rwanda	Lawal et al, 2015

51	*Artabotrys brachypetalus*	Annonaceae	South Africa	Lawal et al, 2015
52	*Artabotrys monteiroae*	Annonaceae	South Africa	Lawal et al, 2015
53	*Artemisia absinthium*	Asteraceae	Egypt	Lawal et al, 2015
54	*Artemisia afra*	Asteraceae	South Africa	Lawal et al, 2015
55	*Artemisia annua*	Asteraceae	Asia	Artemesinin
56	*Artemisia gorgonum*	Asteraceae	Cape Verde	Silva et al, 2011
57	*Artocarpus communis*	Moraceae	Cameroon	Lawal et al, 2015
58	*Asparagus virgatus*	Asparagaceae	South Africa	Lawal et al, 2015
59	*Aspidosperma desmanthum*	Apocynaceae	Brazil	Silva et al, 2011
60	*Aspidosperma vargasii*	Apocynaceae	Brazil	Silva et al, 2011
61	*Aspilia africana*	Asteracae	Nigeria	Lawal et al, 2015
62	*Aster squamatous*	Compositae	Egypt	Lawal et al, 2015
63	*Asystasia gangetica*	Acanthaceae	South Africa	Lawal et al, 2015
64	*Autranella congolensis*	Sapotaceae	Congo	Lawal et al, 2015
65	*Azadirachta indica*	Meliaceae	Nigeria	Lawal et al, 2015
66	*Baillonella toxisperma*	Sapotaceae	Benin	Lawal et al, 2015
67	*Balanites aegyptiaca*	Balanitaceae	Sudan/Togo	Lawal et al, 2015
68	*Barringtonia racemosa*	Lecythidaceae	South Africa	Lawal et al, 2015
69	*Bersama abyssinica*	Melianthaceae	Ivory Coast	Lawal et al, 2015
70	*Berula erecta*	Apiaceae	South Africa	Lawal et al, 2015
71	*Beta vulgaris*	Chenopodiaceae	Egypt	Lawal et al, 2015
72	*Bidens engleri*	Asteraceae	Burkina Faso	Lawal et al, 2015
73	*Bidens pilosa*	Asteraceae	South Africa/Brazil	Lawal et al, 2015; Silva et al, 2011
74	*Boscia angustifolia*	Capparaceae	Mali	Lawal et al, 2015
75	*Boswellia dalzielii*	Burceraceae	Benin/Nigeria	Lawal et al, 2015
76	*Bridelia cathartica*	Euphorbiaceae	Mozambique	Silva et al, 2011
77	*Bridelia ferruginea*	Euphorbiaceae	Angola	Silva et al, 2011
78	*Bridelia micrantha*	Euphorbiaceae	Mozambique/South Africa	Lawal et al, 2015
79	*Bridelia mollis*	Phyllanthaceae	South Africa	Lawal et al, 2015
80	*Bruguiera gymnorhiza*	Rhizophoraceae	South Africa	Lawal et al, 2015
81	*Burchellia bubalina*	Rubiaceae	South Africa	Lawal et al, 2015
82	*Byrsocarpus coccineus*	Connaraceae	Benin	Lawal et al, 2015
83	*Cadaba farinosa*	Capparaceae	Kenya	Lawal et al, 2015
84	*Caesalpinia bonduc*	Caesalpiniaceae	Ghana	Lawal et al, 2015
85	*Calycobolus sp.*	Convolvulaceae	Congo	Lawal et al, 2015
86	*Camellia sinensis*	Theaceae	Egypt	Lawal et al, 2015
87	*Capparis tomentosa*	Capparaceae	South Africa	Lawal et al, 2015
88	*Caralluma tuberculata*	Asclepiadaceae	Congo	Lawal et al, 2015
89	*Cardiospermum halicacabum*	Sapindaceae	South Africa	Lawal et al, 2015
90	*Carica papaya*	Caricaceae	Nigeria	Lawal et al, 2015
91	*Carissa edulis*	Apocynaceae	Kenya/South Africa	Lawal et al, 2015
92	*Carpolobia lutea*	Polygalaceae	Benin	Lawal et al, 2015
93	*Carapichea ipecacuanha*	Rubiaceae	Egypt	Lawal et al, 2015
94	*Casearia sylvestris*	Salicaceae	Brazil	Silva et al, 2011
95	*Cassia abbreviata*	Fabaceae	Mozambique	Lawal et al, 2015
96	*Cassia alata*	Caesalpiniaceae	Ivory Coast	Lawal et al, 2015
97	*Cassia arereh*	Fabaceae	Sudan	Lawal et al, 2015
98	*Cassia occidentalis*	Caesalpiniaceae	Congo/Ivory Coast/ Ghana/Mozambique	Lawal et al, 2015
99	*Cassia podocarpa*	Ceasalpiniaceae	Burkina Faso	Lawal et al, 2015
100	*Cassia sieberiana*	Fabaceae	Nigeria	Lawal et al, 2015
101	*Cassia singueana*	Fabaceae	Nigeria	Lawal et al, 2015

102	*Cassia tora*	Caesalpiniaceae	Sudan	Lawal et al, 2015
103	*Catha edulis*	Celastraceae	South Africa	Lawal et al, 2015
104	*Cecropia pachystachya*	Urticaceae	Brazil	Silva et al, 2011
105	*Cedrela odorata*	Meliaceae	Brazil/São Tomé	Silva et al, 2011
106	*Celtis integrifolia*	Ulmaceae	Burkina Faso	Lawal et al, 2015
107	*Centella asiatica*	Apiaceae	South Africa	Lawal et al, 2015
108	*Cephalanthus natalensis*	Rubiaceae	South Africa	Lawal et al, 2015
109	*Cestrum laevigatum*	Solanaceae	São Tomé	Silva et al, 2011
110	*Chenopodium murale*	Chenopodiaceae	Egypt	Lawal et al, 2015
111	*Cichorium endivia*	Asteraceae	Egypt	Lawal et al, 2015
112	*Cichorium intybus*	Asteraceae	Egypt	Lawal et al, 2015
113	*Cinchona calisaya*	Rubiaceae	South America	Quinine
114	*Cinnamomum cassia*	Lauraceae	Egypt	Lawal et al, 2015
115	*Cissus populnea*	Amplidaceae	Nigeria	Lawal et al, 2015
116	*Cissus quadrangularis*	Vitaceae	Mali	Lawal et al, 2015
117	*Citrullus colocynthis*	Cucurbitaceae	Sudan	Lawal et al, 2015
118	*Citrus aurantifolia*	Rutaceae	Ghana	Lawal et al, 2015
119	*Citrus limon*	Rutaceae	Nigeria	Lawal et al, 2015
120	*Citrus reticulata*	Rutaceae	Egypt	Lawal et al, 2015
121	*Clausena anisata*	Rutaceae	South Africa	Lawal et al, 2015
122	*Cleistopholis patens*	Annonaceae	Ghana	Lawal et al, 2015
123	*Clematis brachiata*	Ranunculaceae	South Africa	Lawal et al, 2015
124	*Cleome rutidosperma*	Cleomaceae	Cameroon	Lawal et al, 2015
125	*Clerodendrum glabrum*	Verbenaceae	South Africa	Lawal et al, 2015
126	*Clutia hirsuta*	Euphorbiaceae	South Africa	Lawal et al, 2015
127	*Clutia pulchella*	Euphorbiaceae	South Africa	Lawal et al, 2015
128	*Cnestis ferruginia*	Connaraceae	Ghana	Lawal et al, 2015
129	*Cochlospermum tinctorium*	Cochlospermaceae	Guinea-Bissau	Silva et al, 2011
130	*Cocos nucifera*	Arecaceae	Nigeria	Lawal et al, 2015
131	*Combretum collinum*	Combretaceae	Burkina Faso	Lawal et al, 2015
132	*Combretum glutinosum*	Combretaceae	Burkina Faso	Lawal et al, 2015
133	*Combretum molle*	Combretaceae	Burkina Faso	Lawal et al, 2015
134	*Combretum sericeum*	Combretaceae	Burkina Faso	Lawal et al, 2015
135	*Combretum zeyheri*	Combretaceae	South Africa	Lawal et al, 2015
136	*Commiphora kerstingii*	Burseraceae	Nigeria	Lawal et al, 2015
137	*Conyza aegyptiaca*	Asteraceae	Rwanda	Lawal et al, 2015
138	*Conyza albida*	Asteraceae	South Africa	Lawal et al, 2015
139	*Conyza dioscoridis*	Compositae	Egypt	Lawal et al, 2015
140	*Conyza podocephala*	Asteraceae	South Africa	Lawal et al, 2015
141	*Conyza scabrida*	Asteraceae	South Africa	Lawal et al, 2015
142	*Copaifera religiosa*	Fabaceae	Gabon	Lawal et al, 2015
143	*Corchorus olitorius*	Tiliaceae	Egypt	Lawal et al, 2015
144	*Crateva religiosa*	Capparidaceae	Benin	Lawal et al, 2015
145	*Crinum macowanii*	Amaryllidaceae	South Africa	Lawal et al, 2015
146	*Crossopteryx febrifuga*	Rubiaceae	Mozambique	Lawal et al, 2015; Silva et al, 2011
147	*Crotalaria burkeana*	Fabaceae	South Africa	Lawal et al, 2015
148	*Croton gratissimus*	Euphorbiaceae	South Africa	Lawal et al, 2015
149	*Croton menyharthii*	Euphorbiaceae	South Africa	Lawal et al, 2015
150	*Croton zambesicus*	Euphorbiaceae	Sudan	Lawal et al, 2015
151	*Cryptolepis sanguinolenta*	Apocynaceae	Guinea-Bissau	Silva et al, 2011
152	*Cucumis meohuliferus*	Curcurbitaceae	Burkina Faso	Lawal et al, 2015
153	*Cucurbita maxima*	Cucurbitaceae	Brazil	Silva et al, 2011

154	*Curcuma aromatic*	Zingebracea	Egypt	Lawal et al, 2015
155	*Cussonia spicata*	Araliaceae	South Africa	Lawal et al, 2015
156	*Cymbopogon citratus*	Poaceae	Cameroon/Nigeria	Lawal et al, 2015
157	*Cymbopogon giganteus*	Poaceae	Benin	Lawal et al, 2015
158	*Cymbopogon nardus*	Poaceae	Benin	Lawal et al, 2015
159	*Cymbopogon proximus*	Poaceae	Egypt	Lawal et al, 2015
160	*Cymbopogon schoenanthus*	Poaceae	Benin/Sudan	Lawal et al, 2015
161	*Cymbopogon validu*	Poaceae	South Africa	Lawal et al, 2015
162	*Cyperus alopecuroides*	Cyperaceae	Egypt	Lawal et al, 2015
163	*Cyperus rotundus*	Cyperacea	Egypt	Lawal et al, 2015
164	*Dalhousiea africana*	Leguminosae	Congo	Lawal et al, 2015
165	*Daniellia oliveri*	Fabaceae	Nigeria	Lawal et al, 2015
166	*Daucus carota*	Apiaceae	Egypt	Lawal et al, 2015
167	*Desmodium velutinum*	Fabaceae	Burkina Faso	Lawal et al, 2015
168	*Desmostachya bipinnata*	Poaceae	Egypt	Lawal et al, 2015
169	*Dialium guineense*	Leguminosae	Benin	Lawal et al, 2015
170	*Dicerocaryum eriocarpum*	Pedaliaceae	Namibia	Lawal et al, 2015
171	*Dichrostachys cinerea*	Fabaceae	South Africa	Lawal et al, 2015
172	*Diosma sp.*	Rutaceae	South Africa	Lawal et al, 2015
173	*Diospyros abyssinica*	Ebenaceae	Uganda	Krief et al, 2006
174	*Diospyros mespiliformis*	Ebenaceae	South Africa	Lawal et al, 2015
175	*Diplorhynchus condylocarpon*	Apocynaceae	South Africa	Lawal et al, 2015
176	*Dodonaea viscosa*	Sapindaceae	South Africa	Lawal et al, 2015
177	*Drypetes gerrardii*	Meliaceae	South Africa	Lawal et al, 2015
178	*Drypetes gossweileri*	Euphorbiaceae	Congo	Lawal et al, 2015
179	*Ekebergia capensis*	Meliaceae	South Africa	Lawal et al, 2015
180	*Elaeis guineensis*	Palmaceae	Ghana	Lawal et al, 2015
181	*Elephantorrhiza elephantina*	Fabaceae	South Africa	Lawal et al, 2015
182	*Emblica officinalis*	Phyllanthaceae	Egypt	Lawal et al, 2015
183	*Enantia chlorantha*	Annonaceae	Congo	Lawal et al, 2015
184	*Entada africana*	Fabaceae	Togo	Lawal et al, 2015
185	*Entandrophragma angolense*	Meliaceae	Cameroon	Lawal et al, 2015
186	*Entandrophragma palustre*	Meliaceae	Congo	Lawal et al, 2015
187	*Erigeron floribundus*	Asteracea	Ivory Coast	Lawal et al, 2015
188	*Eruca sativa*	Brassicaceae	Egypt	Lawal et al, 2015
189	*Erythrina senegalensis*	Fabaceae	Nigeria/Ivory Coast	Lawal et al, 2015
190	*Esenbeckia febrifuga*	Rutaceae	Brazil	Silva et al, 2011
191	*Euclea natalensis*	Ebenaceae	South Africa	Lawal et al, 2015
192	*Eucomis autumnalis*	Asparagaceae	South Africa	Lawal et al, 2015
193	*Euphorbia heterophylla*	Euphorbiaceae	South Africa	Lawal et al, 2015
194	*Euphorbia hirta*	Euphorbiaceae	Congo/Nigeria/Ivory Coast/Angola	Lawal et al, 2015
195	*Euphorbia tirucalli*	Euphorbiaceae	South Africa	Lawal et al, 2015
196	*Zanthoxylum gilletii*	Rutaceae	Ivory Coast	Lawal et al, 2015
197	*Zanthoxylum zanthoxyloides*	Rutaceae	Nigeria	Lawal et al, 2015
198	*Ficus capensis*	Moraceae	Ivory Coast	Lawal et al, 2015
199	*Ficus capreifolia*	Moraceae	Burkina Faso	Lawal et al, 2015
200	*Ficus carica*	Moraceae	Egypt	Lawal et al, 2015
201	*Ficus platyphylla*	Moraceae	Nigeria	Lawal et al, 2015
202	*Ficus thonningii*	Moraceae	Nigeria	Lawal et al, 2015
203	*Flacourtia indica*	Flacourtiaceae	South Africa	Lawal et al, 2015
204	*Flueggea virosa*	Euphorbiaceae	South Africa	Lawal et al, 2015
205	*Afrostyrax lepidophyllus*	Huaceae	Congo	Lawal et al, 2015

206	*Fuerstia africana*	Lamiaceae	Rwanda	Lawal et al, 2015
207	*Funtumia elastica*	Apocynaceae	Ivory Coast	Lawal et al, 2015
208	*Garcinia kola*	Clusiaceae	Congo	Lawal et al, 2015
209	*Garcinia punctata*	Clusiaceae	Congo	Lawal et al, 2015
210	*Gardenia jovis tonatis*	Rubiaceae	Sudan	Lawal et al, 2015
211	*Gardenia lutea*	Rubiacene	Sudan	Lawal et al, 2015
212	*Geissospermum sericeum*	Apocynaceae	Brazil	Silva et al, 2011
213	*Gloriosa superba*	Colchicaceae	South Africa	Lawal et al, 2015
214	*Glycyrrhiza glabra*	Fabaceae	Egypt	Lawal et al, 2015
215	*Gnidia cuneata*	Thymelaeaceae	South Africa	Lawal et al, 2015
216	*Gnidia kraussiana*	Thymelaeaceae	South Africa	Lawal et al, 2015
217	*Gomphocarpus fruticosus*	Apocynaceae	South Africa	Lawal et al, 2015
218	*Guiera senegalensis*	Combretaceae	Nigeria/Guinea-Bissau	Silva et al, 2011
219	*Harungana madagascariensis*	Hypencaceae	Congo/Guinea-Bissau/Nigeria	Lawal et al, 2015; Silva et al, 2011
220	*Helianthus annus*	Poaceae	Sudan	Lawal et al, 2015
221	*Helichrysum nudifolium*	Asteraceae	South Africa	Lawal et al, 2015
222	*Helichrysum pedunculatum*	Asteraceae	South Africa	Lawal et al, 2015
223	*Heliotropium indicum*	Boraginaceae	Benin	Lawal et al, 2015
224	*Hermannia depressa*	Sterculiaceae	South Africa	Lawal et al, 2015
225	*Hexalobus crispiflorus*	Annonaceae	Angola	Silva et al, 2011
226	*Hibiscus sabdariffa*	Malvaceae	Egypt	Lawal et al, 2015
227	*Hippobromus pauciflorus*	Sapindaceae	South Africa	Lawal et al, 2015
228	*Hypericum aethiopicum*	Hypericaceae	South Africa	Lawal et al, 2015
229	*Hyphaene thebaica*	Arecaceae	Egypt	Lawal et al, 2015
230	*Hypoxis colchicifolia*	Hypoxidaceae	South Africa	Lawal et al, 2015
231	*Hyptis pectinata*	Lamiaceae	South Africa	Lawal et al, 2015
232	*Hyptis spicigera*	Lamiaceae	Burkina Faso	Lawal et al, 2015
233	*Irvingia gabonensis*	Simaroubaceae	Ivory Coast	Lawal et al, 2015
234	*Isolona hexaloba*	Annonaceae	Congo	Lawal et al, 2015
235	*Jatropha curcas*	Euphorbiaceae	Congo/Nigeria	Lawal et al, 2015
236	*Jatropha tanjorensis*	Euphorbiaceae	Nigeria	Lawal et al, 2015
237	*Justicia flava*	Acanthaceae	South Africa	Lawal et al, 2015
238	*Keetia leucantha*	Rubiaceae	Benin	Lawal et al, 2015
239	*Khaya grandifoliola*	Maliaceae	Nigeria	Lawal et al, 2015
240	*Khaya senegalensis*	Maliaceae	Benin	Lawal et al, 2015
241	*Kigelia africana*	Bignoniaceae	Kenya/South Africa	Lawal et al, 2015
242	*Kirkia wilmsii*	Kirkiaceae	South Africa	Lawal et al, 2015
243	*Lannea discolour*	Anacardiaceae	South Africa	Lawal et al, 2015
244	*Lawsonia inermis*	Lythraceae	Egypt	Lawal et al, 2015
245	*Leonotis leonurus*	Lamiaceae	Mozambique/ South Africa	Lawal et al, 2015
246	*Leonotis nepetifolia*	Lamiaceae	South Africa	Lawal et al, 2015
247	*Leonotis ocymifolia*	Lamiaceae	South Africa	Lawal et al, 2015
248	*Leucas martinicensis*	Lamiaceae	South Africa	Lawal et al, 2015
249	*Lippia javanica*	Verbenaceae	Kenya/South Africa	Lawal et al, 2015
250	*Lonchocarpus cyanescens*	Fabaceae	Nigeria	Lawal et al, 2015
251	*Lophira alata*	Ochnaceae	Nigeria	Lawal et al, 2015
252	*Lophira lanceolata*	Ochnaceae	Burkina Faso	Lawal et al, 2015
253	*Lupinus termis*	Fabaceae	Egypt	Lawal et al, 2015
254	*Macrostylis squarrosa*	Rutaceae	South Africa	Lawal et al, 2015
255	*Maesa lanceolata*	Maesaceae	South Africa	Lawal et al, 2015
256	*Malva parviflora*	Malvacea	Egypt	Lawal et al, 2015

257	*Mammea africana*	Clusiaceae	Congo	Lawal et al, 2015
258	*Mangifera indica*	Anacardiaceae	Cameron/Ivory Coast	Lawal et al, 2015
259	*Manniophyton fulvum*	Euphorbiaceae	Congo	Lawal et al, 2015
260	*Mareya micrantha*	Euphorbiaceae	Ivory Coast	Lawal et al, 2015
261	*Markhamia lutea*	Bignoniaceae	Rwanda	Lawal et al, 2015
262	*Massularia acuminata*	Rubiaceae	Congo	Lawal et al, 2015
263	*Maytenus heterophylla*	Celastraceae	Kenya	Lawal et al, 2015
264	*Maytenus senegalensis*	Celastraceae	South Africa	Lawal et al, 2015
265	*Maytenus undata*	Celastraceae	South Africa	Lawal et al, 2015
266	*Melanthera scandens*	Asteracea	Ivory Coast	Lawal et al, 2015
267	*Melia azedarach*	Meliaceae	Congo	Lawal et al, 2015
268	*Mallotus oppositofolius*	Euphorbiaceae	Cameroon	Lawal et al, 2015
269	*Mentha longifolia*	Labiatae	Egypt	Lawal et al, 2015
270	*Microdesmis keayana*	Pandaceae	Ivory Coast	Lawal et al, 2015
271	*Microglossa pyrifolia*	Asteraceae	Ivory Coast/Rwanda /Kenya	Lawal et al, 2015
272	*Millettia zechiana*	Fabaceae	Ivory Coast	Lawal et al, 2015
273	*Mimusops caffra*	Sapotaceae	South Africa	Lawal et al, 2015
274	*Mimusops obtusifolia*	Sapotaceae	South Africa	Lawal et al, 2015
275	*Mitragyna stipulosa*	Rubiaceae	Nigeria	Lawal et al, 2015
276	*Mitragyna rubrostipulata*	Rubiaceae	Rwanda	Lawal et al, 2015
277	*Momordica balsamina*	Cucurbitaceae	Mozambique/Nigera/<br>South Africa	Lawal et al, 2015,<br>Silva et al, 2011
278	*Momordica cissoides*	Cucurbitaceae	Ghana	Lawal et al, 2015
279	*Morinda lucida*	Rubiaceae	Nigeria	Lawal et al, 2015
280	*Morinda morindoides*	Rubiaceae	Congo/Ghana/Ivory Coast	Lawal et al, 2015
281	*Moringa oleifera*	Moringaceae	Nigeria	Lawal et al, 2015
282	*Morus alba*	Moraceae	Egypt	Lawal et al, 2015
283	*Musanga cecropioides*	Cecropiaceae	Congo	Lawal et al, 2015
284	*Napoleona vogelii*	Lecythidaceae	Congo	Lawal et al, 2015
285	*Nauclea latifolia*	Rubiaceae	Ivory Coast/Nigeria	Lawal et al, 2015
286	*Nicolasia costata*	Asteraceae	Namibia	Lawal et al, 2015
287	*Nigella sativa*	Ranunculaceae	Sudan	Lawal et al, 2015
288	*Ocimum americanum*	Lamiaceae	South Africa	Lawal et al, 2015
289	*Ocimum gratissimum*	Lamiaceae	Congo/Brazil/<br>Nigeria/Benin	Lawal et al, 2015;<br>Silva et al, 2011
290	*Oedera genistifolia*	Asteraceae	South Africa	Lawal et al, 2015
291	*Olea europaea*	Olacaceae	South Africa	Lawal et al, 2015
292	*Oncoba spinosa*	Flacourtiaceae	Ghana	Lawal et al, 2015
293	*Opilia celtidifolia*	Opiliaceae	Burkina Faso/Togo	Lawal et al, 2015
294	*Opuntia ficus-indica*	Cactaceae	Egypt	Lawal et al, 2015
295	*Origanum majorana*	Lamiaceae	Egypt	Lawal et al, 2015
296	*Osteospermum imbricatum*	Asteraceae	South Africa	Lawal et al, 2015
297	*Ozoroa sphaerocarpa*	Anacardiaceae	South Africa	Lawal et al, 2015
298	*Pachypodantium confine*	Annonaceae	Angola	Silva et al, 2011
299	*Pappea capensis*	Sapindaceae	South Africa	Lawal et al, 2015
300	*Parinari curatellifolia*	Chrysobalanaceae	South Africa/Togo	Lawal et al, 2015
301	*Parkia biglobosa*	Leguminosae	Nigeria	Lawal et al, 2015
302	*Parkinsonia aculeata*	Fabaceae	Mozambique/<br>South Africa	Lawal et al, 2015
303	*Parquetina nigrescens*	Asclepiadaceae	Ivory Coast	Lawal et al, 2015
304	*Pavetta corymbosa*	Rubiaceae	Togo	Lawal et al, 2015
305	*Peganum harmal*	Nitrariaceae	Egypt	Lawal et al, 2015
306	*Pelargonium alchemilloides*	Gentianaceae	South Africa	Lawal et al, 2015

307	*Penianthus longifolius*	Menispermaceae	Congo	Lawal et al, 2015
308	*Pentzia globosa*	Asteraceae	South Africa	Lawal et al, 2015
309	*Periploca linearifolia*	Asclepiadacae	Kenya	Lawal et al, 2015
310	*Tabernaemontana hystrix*	Apocynaceae	Brazil	Silva et al, 2011
311	*Phaseolus vulgaris*	Papilionaceae	Egypt	Lawal et al, 2015
312	*Phragmites communis*	Poaceae	Egypt	Lawal et al, 2015
313	*Phyllanthus amarus*	Euphorbiaceae	Nigeria	Lawal et al, 2015
314	*Phyllanthus muellerianus*	Euphorbiaceae	Ivory Coast	Lawal et al, 2015
315	*Physalis angulata*	Solanaceae	Congo/Ivory Coast	Lawal et al, 2015
316	*Picralima nitida*	Apocynaceae	Congo/Ivory Coast	Lawal et al, 2015
317	*Picrolemma sprucei*	Simaroubaceae	Brazil	Silva et al, 2011
318	*Piliostigma thonningii*	Fabaceae	Nigeria/South Africa	Lawal et al, 2015
319	*Pimpinella anisum*	Umbelliferae	Egypt	Lawal et al, 2015
320	*Piper guineense*	Piperaceae	Congo	Lawal et al, 2015
321	*Piper sp.*	Piperaceae	Angola/Brazil	Silva et al, 2011
322	*Piper umbellatum*	Piperaceae	Cameroon	Lawal et al, 2015
323	*Piptadeniastrum africanum*	Leguminosae	Congo	Lawal et al, 2015
324	*Pittosporum tobira*	Pittosporaceae	Mozambique	Lawal et al, 2015
325	*Pittosporum viridiflorum*	Pittosporaceae	South Africa	Lawal et al, 2015
326	*Plantago major*	Plantaginaceae	South Africa	Lawal et al, 2015
327	*Pleiocarpa mutica*	Apocynaceae	Ghana	Lawal et al, 2015
328	*Plumbago auriculata*	Plumbaginaceae	Mozambique	Lawal et al, 2015
329	*Plumbago zeylanica*	Plumbaginaceae	South Africa	Lawal et al, 2015
330	*Polygonum glabrum*	Polgonaceae	Sudan	Lawal et al, 2015
331	*Pollichia campestris*	Illecebraceae	South Africa	Lawal et al, 2015
332	*Polyalthia oliveri*	Annonaceae	Congo	Lawal et al, 2015
333	*Polyalthia suaveolens*	Annonaceae	Congo	Lawal et al, 2015
334	*Piper peltatum*	Piperaceae	Brazil	Silva et al, 2011
335	*Prosopis africana*	Leguminaceae	Nigeria	Lawal et al, 2015
336	*Pseudarthria hookeri*	Fabaceae	South Africa	Lawal et al, 2015
337	*Psiadia punctulata*	Asteraceae	South Africa	Lawal et al, 2015
338	*Psidium guajava*	Myrtaceae	Nigeria/Egypt	Lawal et al, 2015
339	*Psoralea pinnata*	Fabaceae	South Africa	Lawal et al, 2015
340	*Ptaeroxylon obliquum*	Rutaceae	South Africa	Lawal et al, 2015
341	*Pterocarpus angolensis*	Fabaceae	South Africa	Lawal et al, 2015
342	*Pulicaria crispa*	Asteraceae	Sudan	Lawal et al, 2015
343	*Punica granatum*	Lythraceae	Egypt	Lawal et al, 2015
344	*Pupalia lappacea*	Amaranthaceae	Benin	Lawal et al, 2015
345	*Pycnanthus angolensis*	Myristicaceae	Ivory Coast/São Tomé	Lawal et al, 2015,<br>Silva et al, 2011
346	*Pyrenacantha grandifloria*	Icacinaceae	South Africa	Lawal et al, 2015
347	*Pyrenacantha klaineana*	Cacinaceae	Congo	Lawal et al, 2015
348	*Quassia africana*	Simaroubaceae	Congo	Lawal et al, 2015
349	*Quassia amara*	Simaroubaceae	Nigeria	Lawal et al, 2015
350	*Quercus infectoria*	Fagaceae	Egypt	Lawal et al, 2015
351	*Ranunculus multifidus*	Ranunculaceae	South Africa	Lawal et al, 2015
352	*Rapanea melanophloeos*	Myrtaceae	South Africa	Lawal et al, 2015
353	*Rauvolfia caffra*	Apocynaceae	South Africa	Lawal et al, 2015
354	*Rauvolfia vomitoria*	Apocynaceae	Ivory Coast	Lawal et al, 2015
355	*Remijia ferruginea*	Rubiaceae	Brazil	Silva et al, 2011
356	*Rhigiocarya racemifera*	Menispermaceae	Ivory Coast	Lawal et al, 2015
357	*Rhizophora mucronata*	Rhizophoraceae	South Africa	Lawal et al, 2015
358	*Ricinus communis*	Euphorbiaceae	Egypt/South Africa	Lawal et al, 2015

359	*Rothmannia longiflora*	Rubiaceae	Ghana	Lawal et al, 2015
360	*Rourea coccinea*	Connaraceae	Benin	Lawal et al, 2015
361	*Rumex abyssinicus*	Polygonaceae	Rwanda	Lawal et al, 2015
362	*Rumex bequaertii*	Polygonaceae	Rwanda	Lawal et al, 2015
363	*Rumex crispus*	Polygonaceae	South Africa	Lawal et al, 2015
364	*Rumex sagittatus*	Poaceae	South Africa	Lawal et al, 2015
365	*Agathosma*	Rutaceae	South Africa	Lawal et al, 2015
366	*Salix subserrata*	Salicaceae	Egypt	Lawal et al, 2015
367	*Salvia repens*	Lamiaceae	South Africa	Lawal et al, 2015
368	*Sansevieria liberica*	Dracaenaceae	Benin	Lawal et al, 2015
369	*Sarcocephalus latifolius*	Rubiaceae	Guinea-Bissau	Silva et al, 2011
370	*Scaevola plumieri*	Goodeniaceae	South Africa	Lawal et al, 2015
371	*Schefflera actinophylla*	Araliaceae	Mozambique	Lawal et al, 2015
372	*Schefflera umbellifera*	Araliaceae	South Africa	Lawal et al, 2015
373	*Schizozygia coffaeoides*	Apocynaceae	Kenya	Lawal et al, 2015
374	*Schkuhria pinnata*	Asteraceae	South Africa	Lawal et al, 2015
375	*Schrankia leptocarpa*	Mimosaceae	Benin	Lawal et al, 2015
376	*Schumanniophyton magnificum*	Rubiaceae	Cameroon	Lawal et al, 2015
377	*Scolopia zeyheri*	Flacourtiaceae	Kenya	Lawal et al, 2015
378	*Scoparia dulcis*	Scrophulariaceae	Brazil	Silva et al, 2011
379	*Scorodophloeus zenkeri*	Leguminosae	Congo	Lawal et al, 2015
380	*Securidaca longipedunculata*	Polygalaceae	Mali	Lawal et al, 2015
381	*Securinega virosa*	Euphorbiaceae	Burkina Faso	Lawal et al, 2015
382	*Senecio oxyriifolius*	Asteraceae	South Africa	Lawal et al, 2015
383	*Senna abbreviata*	Fabaceae	Mozambique	Silva et al, 2011
384	*Senna alexandrina*	Fabaceae	Sudan	Lawal et al, 2015
385	*Senna didymobotrya*	Fabaceae	Mozambique/<br>South Africa	Lawal et al, 2015
386	*Senna occidentalis*	Fabaceae	Brazil/Mozambique	Silva et al, 2011
387	*Senna petersiana*	Fabaceae	South Africa	Lawal et al, 2015
388	*Sesamum indicum*	Pedaliaceae	Egypt	Lawal et al, 2015
389	*Sesbania sesban*	Leguminosae	Egypt	Lawal et al, 2015
390	*Setaria megaphylla*	Poaceae	South Africa	Lawal et al, 2015
391	*Sida acuta*	Malvaceae	Nigeria	Lawal et al, 2015
392	*Sisymbrium irio*	Brassicaceae	Egypt	Lawal et al, 2015
393	*Solanecio mannii*	Asteraceae	Rwanda	Lawal et al, 2015
394	*Solanum indicum*	Olanaceae	Ivory Coast	Lawal et al, 2015
395	*Solanum nigrum*	Olanaceae	Ivory Coast	Lawal et al, 2015
396	*Solenostemma argel*	Apocynaceae	Egypt/Sudan	Lawal et al, 2015
397	*Sonchous cornatus*	Asteraceae	Sudan	Lawal et al, 2015
398	*Spilanthes mauritiana*	Asteraceae	South Africa	Lawal et al, 2015
399	*Spinacia oleracea*	Chenopodiaceae	Egypt	Lawal et al, 2015
400	*Staudtia kamerunensis*	Myristicaceae	Congo	Lawal et al, 2015
401	*Striga hermonthica*	Orobanchaceae	Nigeria	Lawal et al, 2015
402	*Struchium sparganophorum*	Asteraceae	São Tomé	Silva et al, 2011
403	*Strychnos henningsii*	Strychnaceae	Kenya	Lawal et al, 2015
404	*Strychnos icaja*	Loganiaceae	Congo	Lawal et al, 2015
405	*Strychnos madagascariensis*	Strychnaceae	South Africa	Lawal et al, 2015
406	*Strychnos potatorum*	Strychnaceae	South Africa	Lawal et al, 2015
407	*Strychnos pungens*	Strychnaceae	South Africa	Lawal et al, 2015
408	*Strychnos spinosa*	Loganiaceae	Benin/Ivory Coast	Lawal et al, 2015
409	*Strychnos usambarensis*	Strychnaceae	Kenya	Lawal et al, 2015
410	*Stylosanthes erecta*	Fabaceae	Mali	Lawal et al, 2015

411	*Swartzia madagascariensis*	Leguminosae	Burkina Faso	Lawal et al, 2015
412	*Symphonia globulifera*	Clusiaceae	Congo	Lawal et al, 2015
413	*Syzygium cordatum*	Myrtaceae	South Africa	Lawal et al, 2015
414	*Tabernaemontana elegans*	Apocynaceae	South Africa /Mozambique	Lawal et al, 2015
415	*Tamarindus indica*	Fabaceae	Egypt/Togo	Lawal et al, 2015
416	*Tamarix nilotica*	Tamaricaceae	Egypt	Lawal et al, 2015
417	*Tapinanthus dodoneifolius*	Euphorbiaceae	Burkina Faso	Lawal et al, 2015
418	*Tapinanthus sessilifolius*	Lorantheciae	nigeria	Lawal et al, 2015
419	*Tarchonanthus camphoratus*	Asteraceae	South Africa	Lawal et al, 2015
420	*Tecomaria capensis*	Bignoniaceae	South Africa	Lawal et al, 2015
421	*Tefracera pogge*	Dilleniaceae	Congo	Lawal et al, 2015
422	*Terminalia avicennioides*	Combretaceae	Burkina, Nigeria	Lawal et al, 2015
423	*Terminalia catappa*	Combretaceae	Nigeria	Lawal et al, 2015
424	*Terminalia ivorensis*	Combretaceae	Ghana	Lawal et al, 2015
425	*Terminalia mollis*	Combretaceae	Rwanda	Lawal et al, 2015
426	*Tetradenia riparia*	Lamiaceae	South Africa	Lawal et al, 2015
427	*Tetrapleura tetraptera*	Fabaceae	Congo	Lawal et al, 2015
428	*Thomandersia hensii*	Acanthaceae	Congo	Lawal et al, 2015
429	*Thymus vulgaris*	Lamiaceae	Egypt	Lawal et al, 2015
430	*Tilia cordata*	Tiliaceae	Egypt	Lawal et al, 2015
431	*Tinospora bakis*	Menispermaceae	Burkina Faso/Sudan	Lawal et al, 2015
432	*Tithonia diversifolia*	Asteraceae	Nigeria/Rwanda/ São Tomé	Lawal et al, 2015; Silva et al, 2011
433	*Toddalia asiatica*	Rutaceae	Kenya	Lawal et al, 2015
434	*Trema orientalis*	Ulmaceae	Nigeria	Lawal et al, 2015
435	*Trichilia emetica*	Meliaceae	Benin/Mali/Mozambique	Lawal et al, 2015
436	*Trichilia rubescens*	Meliaceae	Uganda	Krief et al, 2006
437	*Triclisia dictyophylla*	Menispermaceae	Congo	Lawal et al, 2015
438	*Tridax procumbens*	Asteraceae	South Africa	Lawal et al, 2015
439	*Trifolium alexandrinum*	Leguminosae	Egypt	Lawal et al, 2015
440	*Trimeria grandifolia*	Flacourtiaceae	Rwanda	Lawal et al, 2015
441	*Triumfetta welwitschii*	Tiliaceae	South Africa	Lawal et al, 2015
442	*Turraea floribunda*	Meliaceae	South Africa	Lawal et al, 2015
443	*Turraea heterophylla*	Meliaceae	Ghana	Lawal et al, 2015
444	*Uvaria chamae*	Annonaceae	Ghana	Lawal et al, 2015
445	*Uvariopsis congensis*	Annonaceae	Uganda	Krief et al, 2006
446	*Vahlia capensis*	Vahilaceae	Namibia	Lawal et al, 2015
447	*Vangueria infausta*	Rubiaceae	South Africa	Lawal et al, 2015
448	*Vernonia amygdalina*	Asteraceae	Angola/Nigeria /São Tomé	Lawal et al, 2015; Silva et al, 2011
449	*Vernonia brasiliana*	Asteraceae	Brazil	Silva et al, 2011
450	*Vernonia colourata*	Asteraceae	Ghana/South Africa	Lawal et al, 2015
451	*Vernonia fastigiata*	Asteraceae	South Africa	Lawal et al, 2015
452	*Vernonia hirsute*	Asteraceae	South Africa	Lawal et al, 2015
453	*Vernonia mespilifolia*	Asteraceae	South Africa	Lawal et al, 2015
454	*Vernonia myriantha*	Asteraceae	South Africa	Lawal et al, 2015
455	*Vernonia natalensis*	Asteraceae	South Africa	Lawal et al, 2015
456	*Vernonia oligocephala*	Asteraceae	South Africa	Lawal et al, 2015
457	*Virola surinamensis*	Myristicaceae	Brazil	Silva et al, 2011
458	*Vitex doniana*	Verbenaceae	Nigeria	Lawal et al, 2015
459	*Withania somnifera*	Solanaceae	Egypt	Lawal et al, 2015
460	*Ximenia americana*	Olacaceae	South Africa	Lawal et al, 2015
461	*Ximenia caffra*	Olacaceae	South Africa	Lawal et al, 2015

462	*Xylopia parviflora*	Annonaceae	South Africa	Lawal et al, 2015
463	*Xysmalobium undulatum*	Araliaceae	South Africa	Lawal et al, 2015
464	*Zanthoxylum chalybeum*	Rutaceae	Rwanda	Lawal et al, 2015
465	*Zehneria scabra*	Cucurbitaceae	South Africa	Lawal et al, 2015
466	*Zingiber officinale*	Zingebracea	Egypt	Lawal et al, 2015
467	*Ziziphus mucronata*	Rhamnaceae	South Africa	Lawal et al, 2015
468	*Ziziphus spina-christi*	Rhamnaccae	Egypt	Lawal et al, 2015

Artemisia annua (Artemesinin) and *Cinchona calisaya* (Quinine) are not African plants, but were included in the analysis as two known antimalarial plants so as to not overlook potential African relatives; however, none of the OTUs from ape faecal samples matched these two taxa. All other listed plant species are from Africa and have previously been reported to have potential anti-malaria activity[16-18].

Supplementary References

1. Loy, D. E. *et al.* Out of Africa: origins and evolution of the human malaria parasites *Plasmodium falciparum* and *Plasmodium vivax*. *Int. J. Parasitol.* **47**, 87-97 (2017).
2. Guindon, S. *et al.* New algorithms and methods to estimate maximum-likelihood phylogenies: assessing the performance of PhyML 3.0. *Syst. Biol.* **59**, 307-321 (2010).
3. Eriksson, J., Hohmann, G., Boesch, C. & Vigilant, L. Rivers influence the population genetic structure of bonobos (*Pan paniscus*). *Mol. Ecol.* **13**, 3425-3435 (2004).
4. Kawamoto, Y. *et al.* Genetic structure of wild bonobo populations: diversity of mitochondrial DNA and geographical distribution. *PLoS One* **8**, e59660 (2013).
5. Li, Y. *et al.* Eastern chimpanzees, but not bonobos, represent a simian immunodeficiency virus reservoir. *J. Virol.* **86**, 10776-10791 (2012).
6. Perelman, P. *et al.* A molecular phylogeny of living primates. *PLoS Genet.* **7**, e1001342 (2011).
7. Liu, W. *et al.* Multigenomic delineation of *Plasmodium* species of the *Laverania* subgenus infecting wild-living chimpanzees and gorillas. *Genome Biol. Evol.* **8**, 1929-1939 (2016).
8. Duchene, S., Holmes, E. C. & Ho, S. Y. Analyses of evolutionary dynamics in viruses are hindered by a time-dependent bias in rate estimates. *Proc. R. Soc. B* **281,** 20140732 (2014).
9. Liu, W. *et al.* African origin of the malaria parasite *Plasmodium vivax*. *Nat. Commun.* **5**, 3346 (2014).
10. Liu, W. *et al.* Origin of the human malaria parasite *Plasmodium falciparum* in gorillas. *Nature* **467**, 420-425 (2010).
11. National Aeronautics and Space Administration: MODIS. Available at: http://modis.gsfc.nasa.gov/ (accessed 1st May 2016).
12. Tatem, A. J., Goetz, S. J. & Hay, S. I. Terra and Aqua: new data for epidemiology and public health. *Int J. Appl. Earth Obs. Geoinf.* **6**, 33-46 (2004).
13. Weiss, D. J. *et al.* Air temperature suitability for *Plasmodium falciparum* malaria transmission in Africa 2000-2012: a high-resolution spatiotemporal prediction. *Malar. J.* **13**, 171 (2014).
14. Hansen, M. C. *et al.* High-resolution global maps of 21st-century forest cover change. *Science* **342**, 850-853 (2013).

15. Adler, R. F. *et al.* The version 2 Global Precipitation Climatology Project (GPCP) monthly precipitation analysis (1979-Present). *J. Hydrometeor.* **4**, 1147-1167 (2003).

16. Krief, S. *et al.* Bioactive properties of plant species ingested by chimpanzees (*Pan troglodytes schweinfurthii*) in the Kibale National Park, Uganda. *Am. J. Primatol.* **68**, 51-71 (2006).

17. Lawal, B. *et al.* Potential antimalarials from African natural products: A review. *J. Intercult. Ethnopharmacol.* **4**, 318-343 (2015).

18. Silva, J. R. *et al.* A review of antimalarial plants used in traditional medicine in communities in Portuguese-speaking countries: Brazil, Mozambique, Cape Verde, Guinea-Bissau, Sao Tome and Principe and Angola. *Mem. Inst. Oswaldo Cruz* **106 Suppl 1**, 142-158 (2011).
