# Supplementary material for: Wild bonobos host geographically restricted malaria parasites including a putative new Laverania species
Source: Nat Commun. 2017 Nov 21;8:1635. doi: 10.1038/s41467-017-01798-5 (PMC5696340; doi:10.1038/s41467-017-01798-5)
Supplement: Supplementary file 2 — Description of Additional Supplementary Files [file 41467_2017_1798_MOESM2_ESM.pdf]

Description of Supplementary Files

File Name: Supplementary Data 1
Description: GenBank accession numbers of ape *Plasmodium* sequences.

File Name: Supplementary Data 2
Description: Bonobo mitochondrial haplotypes derived from different collection sites.
